# Comparison and evaluation of methods for generating differentially expressed gene lists from microarray data

**DOI:** 10.1186/1471-2105-7-359

**Published:** 2006-07-26

**Authors:** Ian B Jeffery, Desmond G Higgins, Aedín C Culhane

**Affiliations:** 1 Bioinformatics, Conway Institute, University College Dublin, Belfield, Dublin 4, Ireland; 2 Department of Biostatistics and Computational Biology, Dana-Farber Cancer Institute, Mayer 232, 44 Binney Street, Boston, MA 02115, USA

## Abstract

**Background:**

Numerous feature selection methods have been applied to the identification of differentially expressed genes in microarray data. These include simple fold change, classical t-statistic and moderated t-statistics. Even though these methods return gene lists that are often dissimilar, few direct comparisons of these exist. We present an empirical study in which we compare some of the most commonly used feature selection methods. We apply these to 9 publicly available datasets, and compare, both the gene lists produced and how these perform in class prediction of test datasets.

**Results:**

In this study, we compared the efficiency of the feature selection methods; significance analysis of microarrays (SAM), analysis of variance (ANOVA), empirical bayes t-statistic, template matching, maxT, between group analysis (BGA), Area under the receiver operating characteristic (ROC) curve, the Welch t-statistic, fold change, rank products, and sets of randomly selected genes. In each case these methods were applied to 9 different binary (two class) microarray datasets. Firstly we found little agreement in gene lists produced by the different methods. Only 8 to 21% of genes were in common across all 10 feature selection methods. Secondly, we evaluated the class prediction efficiency of each gene list in training and test cross-validation using four supervised classifiers.

**Conclusion:**

We report that the choice of feature selection method, the number of genes in the genelist, the number of cases (samples) and the noise in the dataset, substantially influence classification success. Recommendations are made for choice of feature selection. Area under a ROC curve performed well with datasets that had low levels of noise and large sample size. Rank products performs well when datasets had low numbers of samples or high levels of noise. The Empirical bayes t-statistic performed well across a range of sample sizes.

## Background

Microarrays enable the simultaneous measurement of the expression levels of tens of thousands of genes and have found widespread application in biological and biomedical research. The use of microarrays to discover genes, which are differentially expressed between two or more groups of patients has many applications. These include the identification of disease biomarkers that may be important in the diagnoses of the different types and subtypes of diseases [1]. Although increasing numbers of multi-class microarray studies are performed, the vast majority continue to be two class (binary) studies, for example where a control and a treatment are examined. In this case, the object of the study, is to determine the genes that are differentially expressed between the two classes. The number of gene probes represented on microarray chips may exceed 50,000 and the number of cases (samples) in microarray studies is frequently limited. This presents a considerable dimensionality issue, which together with the noise inherent in microarray data is a significant challenge to any feature selection approach.

Numerous feature selection methods have been applied to the detection of differentially expressed genes on microarrays. Different methods produce gene lists that are strikingly different [2], yet few studies have compared methods. This is largely due to the lack of benchmark datasets that contain sufficient numbers of known true positive and true negative expressed genes. Studies have verified the expression of detected genes using experimental techniques like RT-PCR [3,4]. However, though RT-PCR verifies the prediction success of a subset of true positives, it provides no indication of the number of true positives or negatives falsely predicted.

Several studies have examined feature selection by investigating the consistency between gene lists from small subsets of samples and those from the full dataset [5], or using a bootstrap method to generate simulated datasets from real datasets [6]. This approach is limited in that it assumes that gene lists generated on the full dataset are correct. A number of studies have used simulated data where the truly regulated genes are known [7-10]. Although simulated data sidesteps many problems, it is unclear whether these simulated datasets realistically reflect the noise inherent in real microarray data. To address these issues, Choe et al (2005) [11] generated binary (two class) microarray dataset with artificial cRNA samples which contain known quantities of "spike in" targets, of which approximately one third were spiked-in differentially. The differentially spike-in targets provided genes with known differentially "expression" ranging from 1.2 – 4-fold between the two classes [16]. These data provide a substantial resource, but contain only six samples. It would be difficult for these 6 cases to represent the complete biological and technical noise inherent in a typical microarray experiment. Due to these limitations, in this study, we apply feature selection methods to 9 real binary (two class) microarray datasets. These datasets include the well-known publicly available colon [12], lymphoma [13] and leukaemia datasets [14,15]. We applied 10 commonly used feature selection methods to these datasets.

The gene lists produced were evaluated using two criteria. The first was the similarity in content between gene lists derived using the different methods. The second was the effectiveness of each gene list to form a gene classifier which could predict the class of a test sample. In using classification to rank feature selection methods, we are assuming that a better gene list should discriminate classes in the data more effectively. A better gene list should provide better input information which will produce a more effective classifier. Therefore it is possible to train a classification model using a particular set of genes, and test how well this model discriminates between classes when applied to a separate blind test dataset. The test dataset can not be used for feature selection or classifier training. The prediction strength of the model is a measure of the power of the input gene list. Therefore it is possible to rank gene lists and assesses the performance feature selection methods.

We also examine the impact that a reduction in sample number has on the performance of feature selection methods. The problem of too few cases is a considerable practical obstacle faced in most microarray data analyses. Typically, the number of samples in a microarray study is limited by cost and/or the availability of sufficient biological material. We make recommendations of feature selection approaches which are most suited to different data structures.

## Results

### Similarity of gene lists

We assessed the overlap between gene lists produced by different feature selection methods. The 10 feature selection methods were applied to the full dataset, 50 percent of samples in the dataset, and to subsets of size 15, 10 and 5 samples per class. To limit sampling bias sample subsets were randomly selected 10 times. Ranked lists of differentially expressed genes were produced using each of the 11 feature selection approaches (10 methods and random). We examined the top 50, 100 and 200 mostly highly ranked genes and recorded the proportion of genes that were different between gene lists. Results were obtained for all 9 datasets (Table 1). A comparison of the overlap between these ranked gene lists are shown as dendrograms in figure 2.

The clusters of methods were consistent when gene lists of the top genes 50, 100, or 200 were compared. Figure 2 shows representative dendrograms comparing the overlap of the 100 most highly ranked genes averaged over all 9 datasets. The individual dendrograms followed by their corresponding percentage matices, for each of the datasets, can be found in additional files 1, 2, 3, 4. Interestingly, only 21.6% of the top 100 genes are present in all 10 gene lists when the full datasets are examined (figure 2A). The set of randomly selected genes did not cluster with any of the 10 feature selection methods and was an outlier.

It can be seen from the topology of the dendrograms in figure 2 that there are two main clusters. The first cluster, consisting of fold change methods (BGA, fold change, rank products) had ~58% identical gene lists. The second cluster contained two subgroups. Gene lists in first subgroup were obtained using the Welch t-statistics methods (Welch t-statistic and maxT) and SAM, and were 87.4% identical when produced from full datasets (figure 2A). The second subgroup consisted of ANOVA, template matching, and the Bayesian t-statistic. ANOVA and template matching produced gene lists which were identical in content. Gene lists produced using the Bayesian t-statistic was very similar to these with 97.2% overlap in gene content. Although ROC falls in neither subgroup, its gene lists shares 74.9% of genes with ANOVA, template matching and the Bayesian t-statistic, and 69% identity with the Welch t-statistic, maxT and SAM subgroup. This topology was consistent when gene lists were produced using feature selection methods applied to 50% of the data (figure 2B).

However as the number of samples is reduced, the challenge of estimating gene variance is increased. When sample size is reduced further to 10 samples per class, the topology of second cluster changes dramatically (figure 2C). The distance between the Welch t-statistic and maxT is reduced (figure 2C), as there is less information available when sample permutation is performed. There is greater difference in gene content between gene lists produced by the two modified t-statistics (82.7% similarity) and the other t-statistic methods (89.5% similarity).

When the sample size is reduced even further to 5 samples per class, we observed that the overlap in genes lists between all methods drops to only 8.6% (figure 2D). The distinction between the modified t-statistics and the other methods is even more apparent (figure 2D). Interestingly, the ROC method is most affected by the reduction in samples size and appears as an outlier of the second group when the sample size falls below 15 samples per class (figure 2C, D). In contrast, the first cluster (BGA, fold change, rank products) was not affected to this same extent when sample size was reduced.

These analyses show that sample size clearly affects ranked gene lists produced by different feature selection methods, and that different methods are more robust to a reduction in sample size.

### Gene lists as classifiers

**Figure 1:**
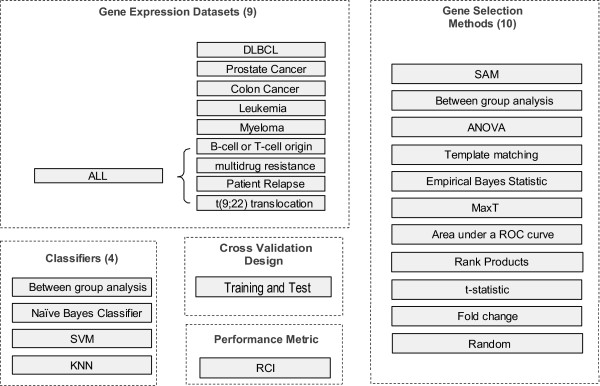
**Experimental design used to study the classifier power of genes lists from different feature selection methods**. The most highly ranked genes were selected from 9 gene expression datasets using 11 feature selection approaches (10 methods and random). The power of these gene lists (of length between 2 and 100 genes) to form classifiers was assessed using four supervised classification methods. In each case genes were selected and classifiers trained using a training dataset. They were tested using training and test cross validation. The cumulative relative classifier information (RCI) score was recorded for each classification.

Gene lists were assessed by comparing the success of each gene list as a classifier (figure 1). All ranked gene lists of length between 2 and 100 were compared. The success of each feature selection approach is represented as an accumulated RCI score (figure 3A). RCI scores were accumulated over 9 different datasets using all 4 classification methods. It is clear that all methods easily out performed random feature selection. However random feature selection does perform better with increasing numbers of genes.

**Figure 2:**
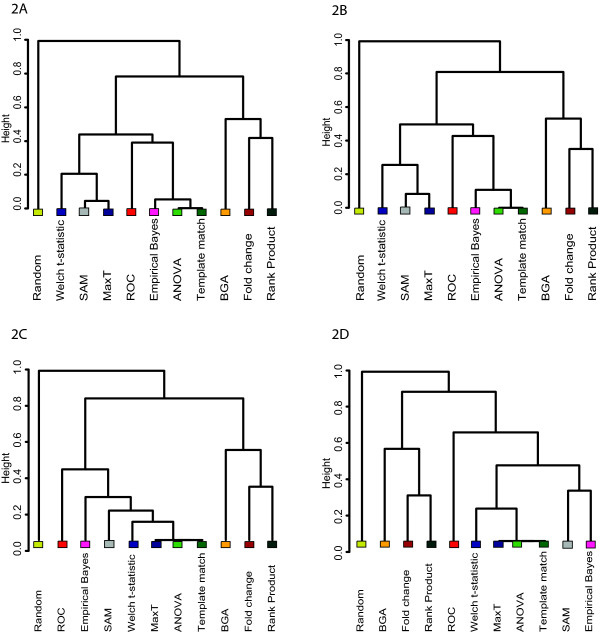
**Overlap in gene lists produced by different feature selection methods**. Each feature selection method was applied to datasets containing A) all samples, B) 50% samples, C) 10 samples per class, or D) 5 samples per class. The overlap of genes ranked in the top 100 by each method was compared using a binary distance metric. Dendrograms show the results of average linkage hierarchical cluster analysis of these scores which were accumulated over all 9 datasets.

**Figure 3:**
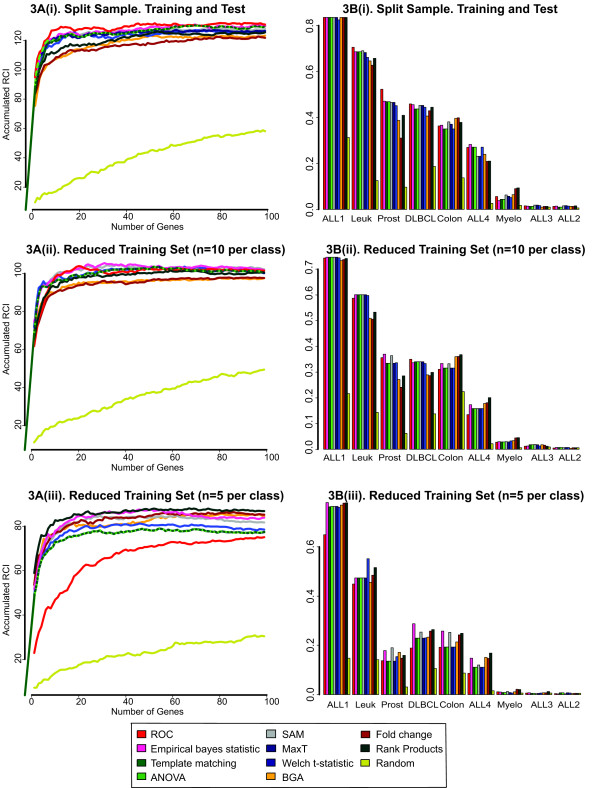
**Gene lists are input to classifiers: training and test cross validation**. Each feature selection method was applied to training datasets that contained i) 50% of samples, ii) 20 samples (10 from each class) or iii) 10 samples (5 from each class), and the most highly ranked genes were selected to generate gene lists of length between 2 and 100 genes. The ability of these gene lists to form successful classifiers was evaluated. The graphs (A) show the prediction success (cumulative RCI values) of these when applied to all 9 datasets and evaluated using four classification tools. Note that the scale of Y-axis (cumulative RCI value) is different between plots. The bar plots (B) show average RCI values showing the success of the top 40 genes, selected by 10 feature selection methods, to form classifiers which can predict the class of blind test data for each of the 9 datasets.

When the datasets were split so as to have the same number of samples per class in the training and test datasets (figure 3A(i)), we observed that the fold methods performed weakly. Fold methods received lower accumulated RCI values than the other methods, over the full range of gene lists lengths (between 2 and 100 genes). Classification performance of classifiers trained with genes lists produced by rank products were better than BGA and fold change but poorer than the other methods. Performance of gene lists from ANOVA and Template matching methods are nearly indistinguishable as shown in figure 3A(i). This is not surprising given that these produced highly overlapping gene lists (figure 2).

Although ANOVA and Template matching has almost identical gene lists, the most highly ranked genes were different when compared to ANOVA. In particular, Template matching had problems with the ALL.4 t(9;22) dataset when the number of genes was below 10. The effect of the variance structure of each of the 9 datasets assessed in figure 3A(i) is shown in figure 3B(i). Figure 3B shows the classification success (average RCI) of gene lists from each datatset, when the top 40 genes are used to build the classifier. Further figures are provided as additional files showing the classification success of the gene lists for each classifier, for each dataset, for the top 20, 40 and 80 genes (additional files 5, 6, 7, 8, 9, 10, 11, 12, 13). The corresponding classification accuracy for each classifier, for each dataset, for the top 20, 40 and 80 genes are provided in additional files 14, 15,16, 17, 18, 19, 20, 21, 22.

The feature selection approaches that perform best on the large sample size datasets were Area under the ROC curve and naïve bayes (figure 3A(i)). However the performance of naïve bayes was only marginally better then the other methods in this training and test cross validation.

The performance of many feature selection approaches was dependent on the variance structure of the dataset (Table 1). It can be seen from figure 3B(i) that the datasets that contribute most to the success of the ROC method are the leukaemia, prostate and DLBCL datasets. The ROC methods performance is as good as any other method in the remaining datasets, excluding colon and myeloma. These two datasets are the noisiest datasets with pooled variances of 0.528 and 0.841 respectively (table 1). The methods that performed the best on these two noisy datasets are the fold change methods. Interestingly, the ROC method performs well on the leukaemia dataset that has the third highest pooled variance of 0.458, and the fold methods performed poorly.

### The effect of reduced numbers of samples per class

In figure 3A(i), large numbers of samples were available in the training datasets. Such large numbers of cases are rare in most microarray studies where replicates are frequently limited. To examine the effect of small sample size, we generated training datasets with fewer samples; only 15, 10 or 5 samples per class. The remainder of the data were used as a blind test set, and the class prediction strength of the training gene lists were assessed using the classification methods, support vector machines [SVM, [16]], BGA [17,18], naïve bayes classification [19], and K-nearest neighbours [KNN, [20]]. When we investigated the training with 15 cases per class (results not shown), we found that the results were similar to figure 3A(ii). That is, fold change methods were still the worst, followed by the t-statistic, but there was less of a difference in the performance between the methods in cluster 2 (dendrograms in figure 2).

As the training set size is reduced further (figures 3A(ii), 3A(iii)) to 10 or 5 samples per class, lower cumulative RCI scores are observed when compared to figure 3A(i), indicating that classifier accuracy is affected by sample size. Given fewer samples, there is less information to determine the usefulness of each gene and there is a greater chance of false positives in a feature selection. Also there is a loss of classification power during the generation of the classification models. A classification model trained on a smaller training dataset is less likely to calculate realistic values for the significance of the genes.

The ranking of feature selection methods is different when the number of samples in the training dataset is reduced. Feature selection methods, such as Area under the ROC curve and maxT that were suited to large numbers of samples (figure 3A(i)) have reduced performance with smaller class sizes (figure 3A(ii), 3A(iii)). In fact, ROC is very sensitive to low sample size and performs poorly compared to the other methods when the number of samples per class is 5 (figure 3A(iii)). This is consistent with the observation that the content of gene lists produced by the ROC method were dramatically affected by low sample size (figure 2D).

In contrast to the large sample size study where all t-statistic methods perform comparably (figure 3A(i)), the modified t-statistic methods (SAM and empirical Bayes) outperform the other t-statistic methods when the sample size is reduced (figures 3A(ii)). MaxT, ANOVA and Template Matching lose power at lower numbers of samples. This maybe attributed to the reduction in information that can be used to calculate the variance obtained from the reduced number of samples. This is supported by the change in the rankings of the t-statistic methods as the number of samples change. When the results from each of the datasets are examined (figure 3B(ii)), the empirical Bayes method and SAM perform comparably to other methods in most of the datasets. But in the prostate, colon, and ALL4 datasets, empirical Bayes method does better then the other t-statistic methods, although in the latter two, empirical bayes method is beaten by the fold methods. When the two datasets with the greatest pooled variance (colon and myeloma) are looked at, we see that the fold change methods especially rank products do well. The fold methods are beaten by other methods in datasets with low variance (Table 1).

When the number of samples is reduced further to 5 samples per class (figure 3A(iii)), the gap between the modified t-statistics and the other t-statistic methods is increased. This is consistent with the separation of these two subgroups in figure 2D. The empiricial bayes statistic is now ranked second, below rank products. Despite being ranked first the rank products method only gets the highest RCI value in two of the datasets. This is because rank products, and to a lesser extent the empirical bayes statistic, was ranked consistently high across the datasets, while the rank of other methods varied.

Overall the empirical bayes t-statistic was most robust. It performed comparably well with any number of cases, but it was outperformed by the ROC method when the number of samples in the training dataset was large, and the rank products method when the number of samples was limiting or when the dataset has a high pooled variance.

## Discussion

Different feature selection methods produce dissimilar gene lists, which can produce dramatically different discrimination performance when trained as gene classifiers. The gene lists produced by the feature selection methods can be grouped broadly according to the manner in which they treat gene variance.

The BGA, fold change and rank products cluster consists of methods that do not model the variance when ranking genes. Although fold change continues to be widely utilised in many studies, this early approach to ranking differentially expressed genes is not optimal. This is because fold change and BGA do not control the variance and so are susceptible to outliers. This is different to rank products, which assumes constant variance across all samples. Rank products compared the product of the ranks of genes in a class with the product of the ranks of genes in the second class. For each gene in the dataset, rank products sorts the genes according to the likelihood of observing their ranked positions on the lists of differentially expressed genes just by chance. Our study has shown that this method performs well with limited numbers of samples and with noisy datasets which agrees with a recent study [21].

In this study the t-statistic methods performed relatively poorly. Given the high levels of noise in microarray data, together with the low samples sizes, computing a t-statistic can be problematic, because the variance estimate (denominator of the t-statistic) can be skewed by the genes which have a low variance. Due to the large numbers of genes studied in microarray datasets, there will always be some genes which have a low standard deviation by chance. Thus, these genes will have a large t-statistic and will be falsely predicted to be differentially expressed.

Classifiers built using gene lists from the ROC method outperformed all other methods when applied to large datasets. High RCI scores were observed even when only a few of the most highly ranked genes were examined. These high RCI scores were maintained when the number of genes examined was increased. It is possible to obtain p-values using this method [22]. However our analysis showed that ROC, like the t-statistic methods, loses power when the number of samples is reduced. ROC ranks a gene based on its power to discriminate between the groups given a threshold false positive rate. This means that it ignores the level of expression of the gene in the two groups. Therefore as the training size decreases, the likelihood of a gene with low variance and no biological meaning being a good discriminator by chance increases. Our results suggest that ROC is an unsuitable method when the sample size is below 30 (class size of 15). This agrees with a previous study which noted the drop in reproducibility of results when the sample size was reduced from n = 70 to n = 30 [6].

When the number of replicates is small, variance estimation is much more challenging. We observed that gene rankings based on most statistics were poor. At low numbers of samples this study finds it difficult to report any differences between methods such as BGA and fold which do not model the variance, and SAM which attempts to model the variance. Equally, in data sets with high variance, fold or non-parametric methods were more powerful than parametric methods. We observed that gene lists from fold change or BGA produced formed comparable or better classifiers to those generated with gene lists from the Welch t-statistic, ANOVA, maxT or template matching. Small noisy datasets are very common in practise, and in these cases rank products can be recommended.

Several modified t-statistics have been proposed to address this problem, of which SAM [3] is arguably the most popular. In this study SAM performed moderately well across most analyses, except when applied to data with low sample size, where it did not outperform the classic fold change. SAM also performs poorly when applied to the noisy datasets. SAM uses a moderated t-statistic, whereby a constant is added to the denominator of the t-statistic. The addition of this constant reduces the chance of detecting genes which have a low standard deviation by chance. The constant is estimated from the sum of the global standard error of the genes. It is reported that the SAM algorithm favours using a large value denominator constant factor, which in turn means the t-statistic depends more on the fold change value [11]. Therefore at low samples sizes it may provide a less reliable estimate of variance, which may explain why simple fold change or non-parametric methods outperform SAM on these types of data. This has also been reported in a number of recent studies [8-10,23].

Although both SAM and the empirical bayes method are moderated t-statistics, the empirical bayes method provides a more complex model of the gene variance. The gene standard error is estimated as a representative value of the variance of the genes at the same level of expression as the gene of interest [24]. We report that in training sets with a large number of cases, the empirical bayes method performed comparably with ANOVA and template matching, although the genes selected by these methods varied slightly. Importantly, unlike most other methods the empirical bayes t-statistic proved equally robust with low numbers of cases. We observed that when the number of cases was small, gene rankings based on the empirical bayes t-statistic proved to be much more reliable than other methods examined in this study. The Bayesian statistic also provides p-values and, has the advantage that it can be expanded to deal with datasets that have more then two classes.

## Conclusion

This study used an indirect method of testing the feature selection methods by using classification models. Using this method we have demonstrated that the empirical bayes statistic, the Area under the ROC curve method and rank products are accurate ways to identify differentially regulated genes in a microarray dataset and that these can produce robust classifiers. The empirical bayes statistic was the most robust method across all sample sizes. When dealing with datasets that have a low pooled variance that contain 15 or more samples, the ROC method is proved to be the most accurate. For datasets that have a high pooled variance or a low number of samples, the rank products method proved useful.

## Methods

All computations were performed using the statistical language R and Bioconductor [25]. The R code is available on request.

### Datasets

We applied feature selection methods to 9 datasets (figure 1). Each dataset is publicly available and data were downloaded from microarray repositories or from the authors' web sites. The post-processed datasets used in this study are available online [26].

#### • DLBCL

The diffuse large B-cell lymphoma (DLBCL) dataset contains 77 samples, 58 of which came from DLBCL patients and 19 follicular lymphoma from a related germinal centre B-cell lymphoma, [13]. The gene expression data were obtained on Affymetrix human 6800 oligonucleotide arrays. The data are available from the Broad Institute website [27].

#### • Prostate

102 samples, 50 of which were non-tumor prostate samples and 52 of which were prostate tumours [28]. The experiments were run on Affymetrix human 95Av2 arrays and the data are available from the Broad Institute website [29].

#### • Colon

The colon cancer dataset consists of 62 samples, 40 tumour samples and 22 normal controls [12]. The gene expression data were obtained on Affymetrix human 6000 arrays and the data are available in the *colonCA *library in Bioconductor [30].

#### • Leukaemia

Gene expression profiles of two types of leukaemia [15]. Samples were derived from 47 patients with acute lymphoblastic leukaemia (ALL) and 25 patients with acute myeloblastic leukaemia. Data were generated on Affymetrix human 6800 arrays and are available in the *golubEsets *library in Bioconductor [30].

#### • Myeloma

Multiple myeloma samples from Tian et al [31] were split into two groups based on the presence or absence of focal lesions of bone. There were 36 patients without and 137 patients with bone lytic lesions. The original paper also used a group of 45 controls. The data were generated using Affymetrix human U95A and were downloaded from Gene Expression Omnibus [32] (accession number: GDS531).

#### • ALL

Gene expression profiles of 128 different individuals with acute lymphoblastic leukaemia [14]. From the annotation available, the samples in this dataset could be split in different ways. We examined four of these splits. These were ALL gene expression profiles with

• **ALL.1**. B-cell (n = 95) or T-cell (n = 33) origin

• **ALL.2**. With (n = 24) and without (n = 101) multidrug resistance (MDR)

• **ALL.3**. Patients that did (n = 65) and did not relapse (n = 35)

• **ALL.4**. From patients with (n = 26) and without (n = 67) the t(9;22) chromosome translocation

The data were generated using Affymetrix human 95Av2 arrays and are available in the *ALL *library in Bioconductor [30].

### Pre-processing of data

The leukaemia, colon and ALL datasets were available from the Bioconductor libraries as mentioned above. The colon data were further processed using quantile normalisation. The leukaemia data was processed by making the min expression value 100 and the max expression value 16000, The data was then logged (base 2). The data for the other datasets were downloaded as raw data files (.cel files) and gene expression values were called using the robust multichip average method [RMA, [33]] and data were quantile normalised using the Bioconductor package, affy. The pooled variance of the datasets were then calculated and the results are shown in table 1.

### Implementation of feature selection methods

10 feature selection methods were applied to each of the datasets (figure 1). These methods were used to rank the genes. We ignored cut-off values such as p-values, that give a probability of a score compared to a null hypothesis.

#### • Fold change

Fold change is a simple ad hoc method. It is often the first method used in microarray analysis. The expression values for each probe are averaged across the samples in each group and the ratio of these averaged values are calculated. The genes are then ranked by this ratio.

#### • ANOVA (t-statistic)

The formula for the t-statistic is the difference in the means over the standard deviation. For 2 groups, this is the equivalent of a 1 way analysis of variance. [34]

#### • Welch t-statistic

The t-statistic assumes that there is an equal variance across each of the groups. This is not always the case, the welch t-statistic does not assume equal variance. For each gene g, the test statistic is

tg=X¯gA−X¯gBSgA2/NA+SgB2/NB
 MathType@MTEF@5@5@+=feaafiart1ev1aaatCvAUfKttLearuWrP9MDH5MBPbIqV92AaeXatLxBI9gBaebbnrfifHhDYfgasaacH8akY=wiFfYdH8Gipec8Eeeu0xXdbba9frFj0=OqFfea0dXdd9vqai=hGuQ8kuc9pgc9s8qqaq=dirpe0xb9q8qiLsFr0=vr0=vr0dc8meaabaqaciaacaGaaeqabaqabeGadaaakeaacqWG0baDdaWgaaWcbaGaem4zaCgabeaakiabg2da9maalaaabaGafmiwaGLbaebadaWgaaWcbaGaem4zaCMaemyqaeeabeaakiabgkHiTiqbdIfayzaaraWaaSbaaSqaaiabdEgaNjabdkeacbqabaaakeaadaGcaaqaamaalyaabaGaem4uam1aa0baaSqaaiabdEgaNjabdgeabbqaaiabikdaYaaaaOqaaiabd6eaonaaBaaaleaacqWGbbqqaeqaaaaakiabgUcaRmaalyaabaGaem4uam1aa0baaSqaaiabdEgaNjabdkeacbqaaiabikdaYaaaaOqaaiabd6eaonaaBaaaleaacqWGcbGqaeqaaaaaaeqaaaaaaaa@48D9@, where X¯gA
 MathType@MTEF@5@5@+=feaafiart1ev1aaatCvAUfKttLearuWrP9MDH5MBPbIqV92AaeXatLxBI9gBaebbnrfifHhDYfgasaacH8akY=wiFfYdH8Gipec8Eeeu0xXdbba9frFj0=OqFfea0dXdd9vqai=hGuQ8kuc9pgc9s8qqaq=dirpe0xb9q8qiLsFr0=vr0=vr0dc8meaabaqaciaacaGaaeqabaqabeGadaaakeaacuWGybawgaqeamaaBaaaleaacqWGNbWzcqWGbbqqaeqaaaaa@308B@ and X¯gB
 MathType@MTEF@5@5@+=feaafiart1ev1aaatCvAUfKttLearuWrP9MDH5MBPbIqV92AaeXatLxBI9gBaebbnrfifHhDYfgasaacH8akY=wiFfYdH8Gipec8Eeeu0xXdbba9frFj0=OqFfea0dXdd9vqai=hGuQ8kuc9pgc9s8qqaq=dirpe0xb9q8qiLsFr0=vr0=vr0dc8meaabaqaciaacaGaaeqabaqabeGadaaakeaacuWGybawgaqeamaaBaaaleaacqWGNbWzcqWGcbGqaeqaaaaa@308D@ denote the sample average intensities in groups A and B, and *S*^2^_*gA *_and *S*^2^_*gB *_denote the sample variances for each group.

#### • MaxT

MaxT was computed using the mt.MaxT function in the Multtest package for Bioconductor in R [35]. It determines the family-wise error rate-adjusted *P *values using the Welch t-statistic. To do this the class labels are permuted, and the Welch t-statistic for each gene is calculated. The maximum Welch t-statistic is recorded for 10,000 random permutations, the distribution of maximum t-statistics is compared with observed values for the statistic, and the *P *for each gene is estimated as the proportion of the maximum permutation-based t-statistics that are greater than the observed value.

#### • SAM

When using the t-statistic it is often the case that small per-gene variances can make small fold-changes statistically significant. Tusher et al. 2001 [3] proposed the SAM (Significance Analysis of Microarrays) method to deal with this problem.

It works by adding a small "fudge factor" to the denominator of the test statistic. This fudge factor is calculated from the distribution of gene-specific standard errors. Thereby eliminating the small variances. SAM was applied using the siggenes package for Bioconductor in R

#### • Empirical bayes statistic

The Empiricial bayes statistic [24] is described as equivalent to shrinkage of the estimated sample variances towards a pooled estimate, resulting in far more stable inference when the number of arrays is small. It returns the log-odds that a gene is differntialy expressed. The higher the score, the more significant the result. The empirical bayes statistic was applied using the the LIMMA package for Bioconductor in R.

#### • Template matching

This is a simple and flexible method to investigate microarray data. A template, or profile, of gene expression, is defined by the experimenter. Genes which match the template, as measured using correlation, are identified as biologically interesting. It has the advantage that it can be used with any number of groups and templates. This means it can be used to find specific biological expression profiles that are of interest to the researcher in multigroup microarray datasets. Template matching were executed as in Pavlidis and Noble [36].

#### • Area under the Roc curve

ROC analysis displays the relationship between the proportion of true positives (sensitivity) and false positives (1-specificity) resulting from each possible decision threshold value in a two-class classification problem. Where classification has occurred, the graph of the output from the ROC analysis forms a curve. The area under this curve can be used as a measure of the accuracy of the test.

This method can be applied to the expression values of a gene belonging to a number of samples belonging to two groups. The area under the ROC curve provides an estimate of the probability that a gene is regulated between the two groups [37].

This method was performed using functions from the ROC library.

#### • Between Group Analysis (BGA)

BGA is a multiple discriminant analysis approach, which uses a dimension reduction technique such as correspondence analysis (COA) or principal component analysis[18]. Instead of dimension reduction of the individual samples as is done in these classical ordination techniques, BGA ordinates the groups. It finds the eigenvectors or axes that discriminate the groups so as to maximise the between group variances. When used with COA, BGA also ordinates the genes, in a way that the most discriminating genes are at the end of the axes. In this way the genes associated with each group are established. This analysis was performed using the ade4 library in R.

#### • Rank Product

The Rank Products method was developed for identifying differentially expressed genes in cDNA expression data [21,23]. It is based on the argument that a gene in an experiment examining *n *genes in *k *replicates, has a probability of being ranked first (rank 1) of 1/*n*^*k *^if the lists were entirely random. Therefore, it is unlikely for a single gene to be in the top position in all replicates if this gene was not differentially expressed. More generally, for each gene *g *in *k *replicates *i*, each examining *n*_*i *_genes, one can calculate the corresponding combined probability as a rank product

RPgup=∏i=1k(ri,gup/ni)
MathType@MTEF@5@5@+=feaafiart1ev1aaatCvAUfKttLearuWrP9MDH5MBPbIqV92AaeXatLxBI9gBaebbnrfifHhDYfgasaacH8akY=wiFfYdH8Gipec8Eeeu0xXdbba9frFj0=OqFfea0dXdd9vqai=hGuQ8kuc9pgc9s8qqaq=dirpe0xb9q8qiLsFr0=vr0=vr0dc8meaabaqaciaacaGaaeqabaqabeGadaaakeaacqWGsbGucqWGqbaudaqhaaWcbaGaem4zaCgabaGaemyDauNaemiCaahaaOGaeyypa0ZaaebmaeaadaqadiqaamaalyaabaGaemOCai3aa0baaSqaaiabdMgaPjabcYcaSiabdEgaNbqaaiabdwha1jabdchaWbaaaOqaaiabd6gaUnaaBaaaleaacqWGPbqAaeqaaaaaaOGaayjkaiaawMcaaaWcbaGaemyAaKMaeyypa0JaeGymaedabaGaem4AaSganiabg+Givdaaaa@47BC@

where *r*_*i*,*g*_^up ^is the position of gene *g *in the list of genes in the *i*th replicate sorted by decreasing fold change, i.e. *r*^up ^= 1 for the most strongly upregulated gene, etc. The genes can then be sorted according to the likelihood of observing their RP value at or above a certain position on the list.

In addition to these methods, a set of random genes were selected from each dataset. This gave a total of 11 methods, which were compared. R scripts to perform these methods are available on request.

### Investigating the overlap in content of feature lists

Each of the 11 feature selection methods were applied to each of the 9 datasets. Each method was applied to all data samples (cases) and to four subsets of each dataset which contained fewer samples. These subsets were 50 percent of the samples, and datasets of 5, 10, or 15 samples per class. 10 random selections of each of these four sample subsets were generated. The overlap of features (genes) in the top 50, 100 and 200 highly ranked genes were counted using the binary distance metric as implemented in the *stats *library in R. This gives the proportion of genes between two lists that are different, ignoring genes that are absent from both. In order to visualise these results, hierarchical clustering was performed using UPGMA/average linkage clustering [38].

### Class prediction success of each feature list

Datasets were divided into training and test datasets. Feature selection and training of classifiers was performed on the training dataset only. The success of each gene list as a classifier was measured using the test dataset. Importantly test datasets were never used in either feature selection or classifier training.

To compare the success of each gene list as a classifier, a classification method was required. It is known that different types of classifiers can respond differently to the same input data. Therefore it was decided to use a number of classification tools: between group analysis [BGA, [17,18]], naïve bayes classification [19], support vector machines [SVM, [16]] and K-nearest neighbours [KNN, [20]].

BGA is a multiple discriminant analysis approach, which uses a dimension reduction technique such as correspondence analysis (COA) or principal component analysis. Instead of dimension reduction of the individual samples as is done in these classical ordination techniques, BGA ordinates the groups. It finds the eigenvectors that separate the groups so as to maximise the between group variances. New samples can then be projected on to these eigenvectors and classified according to their proximity to the centroids of the groups. In this study BGA was implemented using COA. BGA is available in the R library *ade4 *[17], and its extension package *made4 *[39] in Bioconductor.

Naïve bayes simplifies the classification process using the assumption that all features are independent given the class. Although it is generally agreed that this is a poor assumption, the technique has proved robust over a wide range of classification problems. The algorithm estimates the conditional probabilities of an observation belonging to each class by using the joint probabilities of sample observations (genes) and classes. Naïve bayes was implemented using the *limma *library [24] in Bioconductor.

SVM has been applied to the classification of microarray data in a number of studies [40,41]. Binary SVM's look for the maximally separating hyperplane between the closest points of the two classes. In this study we used a linear kernel, and SVM was applied using the *e1071 *library in R.

KNN has been widely used in microarray classification [28,42,43]. When KNN is presented with a test case, it uses Euclidean distances to find a number, *K *of the nearest cases from the training set which have known classes. It then applies a weight to these K nearest cases that is inversely proportional to the distance from the test sample. The predicted class of the sample is then determined by taking the sum of the K weighted samples. KNN with K = 11, was applied using the *class *library in R.

### Cross validation

In each cross validation, the 10 feature selection methods were applied to the data to produce 10 lists of ranked genes. The top *n *genes were selected. The number of genes, *n*, ranged from 2 to 100 inclusive. Thus 990 gene lists were produced from each training dataset. These gene selections were used to train classifier models.

The cross-validation of classifiers was performed using full training and test cross-validation. For 50% sample analysis (figure 3A), data were randomly split into two equal groups. The first group was used as a training dataset for feature selection and classifier training. In training and test cross-validation, all four classification methods, BGA, SVM, KNN and naïve bayes classification were applied. The prediction success of each model was assessed using the blind test dataset. Importantly full cross-validation was performed; the test data were not used for feature selection of gene lists or training of classifiers. The whole process was repeated 10 times to ensure there was no sampling bias in the training or test datasets.

### Examining the effect of sample size

We also examined the efficiency of training datasets with reduced numbers of samples (figure 3B–C). To do this, we created training datasets with only 5, 10 and 15 samples per class. The remainder of the data were used as the blind test set. Again the power of gene lists (length *n *= 2:100) to classify samples in the blind test dataset were recorded. The whole process was repeated 10 times as in the first training and test cross-validation. All four classification methods, BGA, SVM, KNN and naïve bayes classification, were applied to gene lists from each of the 9 datasets.

### The Relative Classifier Information metric

The numbers of correctly predicted cases were counted for each cross validation. Although many studies present these results in terms of the percentage accuracy, this unfortunately does not take into account bias in the number of samples in each class in the dataset being tested. For example, if a dataset with a 100 samples contained 95 normal and 5 diseased, a classifier where all the samples were predicted to be normal would be 95% accurate. This is misleading. Therefore we present the number of correctly classified samples using the relative classifier information metric [RCI, [44]]. The RCI metric is an entropy-based measure that corrects for differences in prior probabilities caused by unequal class size. By taking into account this prior probability, a better measure of classification power is obtained [44].

Given a classifier's performance on a test set, the RCI measure may be derived as follows; Let *Q *be a confusion matrix, so that *q*_*ij *_is the number of times an input (I) whose actual label is *C*_*i *_is labelled *C*_*j*_. *C*_*i *_are the true labels and *C*_*j *_are the labels predicted by a classification model. The probability that I has a true label *C*_*i *_is given by:

P(I∈Ci)=∑jqij∑ijqij
 MathType@MTEF@5@5@+=feaafiart1ev1aaatCvAUfKttLearuWrP9MDH5MBPbIqV92AaeXatLxBI9gBaebbnrfifHhDYfgasaacH8akY=wiFfYdH8Gipec8Eeeu0xXdbba9frFj0=OqFfea0dXdd9vqai=hGuQ8kuc9pgc9s8qqaq=dirpe0xb9q8qiLsFr0=vr0=vr0dc8meaabaqaciaacaGaaeqabaqabeGadaaakeaacqWGqbaucqGGOaakcqWGjbqscqGHiiIZcqWGdbWqdaWgaaWcbaGaemyAaKgabeaakiabcMcaPiabg2da9maalaaabaWaaabeaeaacqWGXbqCdaWgaaWcbaGaemyAaKMaemOAaOgabeaaaeaacqWGQbGAaeqaniabggHiLdaakeaadaaeqaqaaiabdghaXnaaBaaaleaacqWGPbqAcqWGQbGAaeqaaaqaaiabdMgaPjabdQgaQbqab0GaeyyeIuoaaaaaaa@4649@

If an external user was to have knowledge of the distribution of the test-set sample over the classes, they would have some knowledge of the chance of a random sample belonging to each of the classes. Therefore this distribution may be used as a measure of the difficulty of a decision problem. The entropy of the data set before classification can be used to measure the uncertainty associated with a test set before a classification model has been applied and is calculated as:

Hd(I)=∑i−P(I∈Ci)log⁡P(I∈Ci))
 MathType@MTEF@5@5@+=feaafiart1ev1aaatCvAUfKttLearuWrP9MDH5MBPbIqV92AaeXatLxBI9gBaebbnrfifHhDYfgasaacH8akY=wiFfYdH8Gipec8Eeeu0xXdbba9frFj0=OqFfea0dXdd9vqai=hGuQ8kuc9pgc9s8qqaq=dirpe0xb9q8qiLsFr0=vr0=vr0dc8meaabaqaciaacaGaaeqabaqabeGadaaakeaacqWGibasdaWgaaWcbaGaemizaqgabeaakiabcIcaOiabdMeajjabcMcaPiabg2da9maaqafabaGaeyOeI0IaemiuaaLaeiikaGIaemysaKKaeyicI4Saem4qam0aaSbaaSqaaiabdMgaPbqabaGccqGGPaqkcyGGSbaBcqGGVbWBcqGGNbWzcqWGqbaucqGGOaakcqWGjbqscqGHiiIZcqWGdbWqdaWgaaWcbaGaemyAaKgabeaaaeaacqWGPbqAaeqaniabggHiLdGccqGGPaqkcqGGPaqkaaa@4CAB@

The probability that the classifier output (O) will predict a sample as belonging to class *C*_*j *_is;

P(O∈Cj)=∑iqij∑ijqij
 MathType@MTEF@5@5@+=feaafiart1ev1aaatCvAUfKttLearuWrP9MDH5MBPbIqV92AaeXatLxBI9gBaebbnrfifHhDYfgasaacH8akY=wiFfYdH8Gipec8Eeeu0xXdbba9frFj0=OqFfea0dXdd9vqai=hGuQ8kuc9pgc9s8qqaq=dirpe0xb9q8qiLsFr0=vr0=vr0dc8meaabaqaciaacaGaaeqabaqabeGadaaakeaacqWGqbaucqGGOaakcqWGpbWtcqGHiiIZcqWGdbWqdaWgaaWcbaGaemOAaOgabeaakiabcMcaPiabg2da9maalaaabaWaaabeaeaacqWGXbqCdaWgaaWcbaGaemyAaKMaemOAaOgabeaaaeaacqWGPbqAaeqaniabggHiLdaakeaadaaeqaqaaiabdghaXnaaBaaaleaacqWGPbqAcqWGQbGAaeqaaaqaaiabdMgaPjabdQgaQbqab0GaeyyeIuoaaaaaaa@4655@

The probability that a sample belonging to *C*_*i *_is labelled as *C*_*j *_by the classifier is;

P(I∈Ci|O∈Cj)=pij=qij∑iqij
 MathType@MTEF@5@5@+=feaafiart1ev1aaatCvAUfKttLearuWrP9MDH5MBPbIqV92AaeXatLxBI9gBaebbnrfifHhDYfgasaacH8akY=wiFfYdH8Gipec8Eeeu0xXdbba9frFj0=OqFfea0dXdd9vqai=hGuQ8kuc9pgc9s8qqaq=dirpe0xb9q8qiLsFr0=vr0=vr0dc8meaabaqaciaacaGaaeqabaqabeGadaaakeaacqWGqbaucqGGOaakcqWGjbqscqGHiiIZcqWGdbWqdaWgaaWcbaGaemyAaKgabeaakiabcYha8jabd+eapjabgIGiolabdoeadnaaBaaaleaacqWGQbGAaeqaaOGaeiykaKIaeyypa0JaemiCaa3aaSbaaSqaaiabdMgaPjabdQgaQbqabaGccqGH9aqpdaWcaaqaaiabdghaXnaaBaaaleaacqWGPbqAcqWGQbGAaeqaaaGcbaWaaabeaeaacqWGXbqCdaWgaaWcbaGaemyAaKMaemOAaOgabeaaaeaacqWGPbqAaeqaniabggHiLdaaaaaa@4DE1@

Therefore the uncertainty for a sample after classification has occurred is;

HOj(I|O∈Cj)=pjout=∑i−pijlog⁡pij
 MathType@MTEF@5@5@+=feaafiart1ev1aaatCvAUfKttLearuWrP9MDH5MBPbIqV92AaeXatLxBI9gBaebbnrfifHhDYfgasaacH8akY=wiFfYdH8Gipec8Eeeu0xXdbba9frFj0=OqFfea0dXdd9vqai=hGuQ8kuc9pgc9s8qqaq=dirpe0xb9q8qiLsFr0=vr0=vr0dc8meaabaqaciaacaGaaeqabaqabeGadaaakeaacqWGibasdaWgaaWcbaGaem4ta80aaSbaaWqaaiabdQgaQbqabaaaleqaaOGaeiikaGIaemysaKKaeiiFaWNaem4ta8KaeyicI4Saem4qam0aaSbaaSqaaiabdQgaQbqabaGccqGGPaqkcqGH9aqpcqWGWbaCdaqhaaWcbaGaemOAaOgabaGaem4Ba8MaemyDauNaemiDaqhaaOGaeyypa0ZaaabuaeaacqGHsislcqWGWbaCdaWgaaWcbaGaemyAaKMaemOAaOgabeaakiGbcYgaSjabc+gaVjabcEgaNjabdchaWnaaBaaaleaacqWGPbqAcqWGQbGAaeqaaaqaaiabdMgaPbqab0GaeyyeIuoaaaa@54C7@

And the overall uncertainty after classification is;

HO(I|O)=∑jP(O∈Cj)⋅HOj(I|O=Cj)
 MathType@MTEF@5@5@+=feaafiart1ev1aaatCvAUfKttLearuWrP9MDH5MBPbIqV92AaeXatLxBI9gBaebbnrfifHhDYfgasaacH8akY=wiFfYdH8Gipec8Eeeu0xXdbba9frFj0=OqFfea0dXdd9vqai=hGuQ8kuc9pgc9s8qqaq=dirpe0xb9q8qiLsFr0=vr0=vr0dc8meaabaqaciaacaGaaeqabaqabeGadaaakeaacqWGibasdaWgaaWcbaGaem4ta8eabeaakiabcIcaOiabdMeajjabcYha8jabd+eapjabcMcaPiabg2da9maaqafabaGaemiuaaLaeiikaGIaem4ta8KaeyicI4Saem4qam0aaSbaaSqaaiabdQgaQbqabaaabaGaemOAaOgabeqdcqGHris5aOGaeiykaKIaeyyXICTaemisaG0aaSbaaSqaaiabd+eapnaaBaaameaacqWGQbGAaeqaaaWcbeaakiabcIcaOiabdMeajjabcYha8jabd+eapjabg2da9iabdoeadnaaBaaaleaacqWGQbGAaeqaaOGaeiykaKcaaa@50AA@

The reduction in uncertainty due to the classifier is used as the RCI score:

RCI score = *H*_*d *_- *H*_*o*_

A higher RCI score indicates an improvement in classification power. If a dataset has an equal number of samples in each class, the RCI will be 1 if all samples are predicted with perfect accuracy. If the class sizes are unequal the maximum score is <1. In this study, all classification results are presented using the RCI metric. The RCI values are summed across classifiers for each dataset, and thus results are shown as cumulative RCI scores. The above calculations were performed using an R script.

## Authors' contributions

I.B.J procured the necessary data and software, carried out the analyses, analyzed the results and drafted the manuscript. D.G.H and A.C.C. conceived the project, assisted in the design of the study and in drafting of the manuscript. All authors read and approved the final manuscript.

## Supplementary Material

Additional File 1 **Overlap in gene lists produced by different feature selection methods where n = 5 samples per class**. Each feature selection method was applied to datasets containing 5 samples per class. The overlap of genes ranked in the top 100 by each method was compared using a binary distance metric. Dendrograms show the results of average linkage hierarchical cluster analysis of these scores for each dataset. Percentage matricies below each of the dendrograms show the percentage similarity between each of the feature selection methods. Click here for file

Additional File 2 **Overlap in gene lists produced by different feature selection methods where n = 10 samples per class**. Each feature selection method was applied to datasets containing 10 samples per class. The overlap of genes ranked in the top 100 by each method was compared using a binary distance metric. Dendrograms show the results of average linkage hierarchical cluster analysis of these scores for each dataset. Percentage matricies below each of the dendrograms show the percentage similarity between each of the feature selection methods. Click here for file

Additional File 3 **Overlap in gene lists produced by different feature selection methods where n = 50% of the samples per class**. Each feature selection method was applied to datasets containing 50% of the samples per class. The overlap of genes ranked in the top 100 by each method was compared using a binary distance metric. Dendrograms show the results of average linkage hierarchical cluster analysis of these scores for each dataset. Percentage matricies below each of the dendrograms show the percentage similarity between each of the feature selection methods. Click here for file

Additional File 4 **Overlap in gene lists produced by different feature selection methods when applied to each dataset**. Each feature selection method was applied to each of the full datasets. The overlap of genes ranked in the top 100 by each method was compared using a binary distance metric. Dendrograms show the results of average linkage hierarchical cluster analysis of these scores for each dataset. Percentage matricies below each of the dendrograms show the percentage similarity between each of the feature selection methods. Click here for file

Additional File 5 **The RCI scores for each of the individual datasets and individual classification methods where the top 80 genes are used and n = 5 samples per class**. RCI values showing the success of the top 80 genes, selected by the feature selection methods, to form classifiers which can predict the class of blind test data for each of the 9 datasets. These figures show the results for each of the classification methods when a reduced training set of 10 (5 from each class) is used. Click here for file

Additional File 6 **The RCI scores for each of the individual datasets and individual classification methods where the top 80 genes are used and n = 10 samples per class**. RCI values showing the success of the top 80 genes, selected by the feature selection methods, to form classifiers which can predict the class of blind test data for each of the 9 datasets. These figures show the results for each of the classification methods when a reduced training set of 20 (10 from each class) is used. Click here for file

Additional File 7 **The RCI scores for each of the individual datasets and individual classification methods where the top 80 genes are used and n = 50% of the samples per class**. RCI values showing the success of the top 80 genes, selected by the feature selection methods, to form classifiers which can predict the class of blind test data for each of the 9 datasets. These figures show the results for each of the classification methods when a datasets split equally into training and test sets is used. Click here for file

Additional File 8 **The RCI scores for each of the individual datasets and individual classification methods where the top 40 genes are used and n = 5 samples per class**. RCI values showing the success of the top 40 genes, selected by the feature selection methods, to form classifiers which can predict the class of blind test data for each of the 9 datasets. These figures show the results for each of the classification methods when a reduced training set of 10 (5 from each class) is used. Click here for file

Additional File 9 **The RCI scores for each of the individual datasets and individual classification methods where the top 40 genes are used and n = 10 samples per class**. RCI values showing the success of the top 40 genes, selected by the feature selection methods, to form classifiers which can predict the class of blind test data for each of the 9 datasets. These figures show the results for each of the classification methods when a reduced training set of 20 (10 from each class) is used. Click here for file

Additional File 10 **The RCI scores for each of the individual datasets and individual classification methods where the top 40 genes are used and n = 50% of the samples per class**. RCI values showing the success of the top 40 genes, selected by the feature selection methods, to form classifiers which can predict the class of blind test data for each of the 9 datasets. These figures show the results for each of the classification methods when a datasets split equally into training and test sets is used. Click here for file

Additional File 11 **The RCI scores for each of the individual datasets and individual classification methods where the top 20 genes are used and n = 5 samples per class**. RCI values showing the success of the top 20 genes, selected by the feature selection methods, to form classifiers which can predict the class of blind test data for each of the 9 datasets. These figures show the results for each of the classification methods when a reduced training set of 10 (5 from each class) is used. Click here for file

Additional File 12 **The RCI scores for each of the individual datasets and individual classification methods where the top 20 genes are used and n = 10 samples per class**. RCI values showing the success of the top 20 genes, selected by the feature selection methods, to form classifiers which can predict the class of blind test data for each of the 9 datasets. These figures show the results for each of the classification methods when a reduced training set of 20 (10 from each class) is used. Click here for file

Additional File 13 **The RCI scores for each of the individual datasets and individual classification methods where the top 20 genes are used and n = 50% of the samples per class**. RCI values showing the success of the top 20 genes, selected by the feature selection methods, to form classifiers which can predict the class of blind test data for each of the 9 datasets. These figures show the results for each of the classification methods when a datasets split equally into training and test sets is used. Click here for file

Additional File 14 **The percentage accuracy scores for each of the individual datasets and individual classification methods where the top 80 genes are used and n = 5 samples per class**. The percentage accuracy of the top 80 genes, selected by the feature selection methods, to form classifiers which can predict the class of blind test data for each of the 9 datasets. These figures show the results for each of the classification methods when a reduced training set of 10 (5 from each class) is used. Click here for file

Additional File 15 **The percentage accuracy scores for each of the individual datasets and individual classification methods where the top 80 genes are used and n = 10 samples per class**. The percentage accuracy of the top 80 genes, selected by the feature selection methods, to form classifiers which can predict the class of blind test data for each of the 9 datasets. These figures show the results for each of the classification methods when a reduced training set of 20 (10 from each class) is used. Click here for file

Additional File 16 **The percentage accuracy scores for each of the individual datasets and individual classification methods where the top 80 genes are used and n = 50% of the samples per class**. The percentage accuracy of the top 80 genes, selected by the feature selection methods, to form classifiers which can predict the class of blind test data for each of the 9 datasets. These figures show the results for each of the classification methods when a datasets split equally into training and test sets is used. Click here for file

Additional File 17 **The percentage accuracy scores for each of the individual datasets and individual classification methods where the top 40 genes are used and n = 5 samples per class**. The percentage accuracy of the top 40 genes, selected by the feature selection methods, to form classifiers which can predict the class of blind test data for each of the 9 datasets. These figures show the results for each of the classification methods when a reduced training set of 10 (5 from each class) is used. Click here for file

Additional File 18 **The percentage accuracy scores for each of the individual datasets and individual classification methods where the top 40 genes are used and n = 10 samples per class**. The percentage accuracy of the top 40 genes, selected by the feature selection methods, to form classifiers which can predict the class of blind test data for each of the 9 datasets. These figures show the results for each of the classification methods when a reduced training set of 20 (10 from each class) is used. Click here for file

Additional File 19 **The percentage accuracy scores for each of the individual datasets and individual classification methods where the top 40 genes are used and n = 50% of the samples per class**. The percentage accuracy of the top 40 genes, selected by the feature selection methods, to form classifiers which can predict the class of blind test data for each of the 9 datasets. These figures show the results for each of the classification methods when a datasets split equally into training and test sets is used. Click here for file

Additional File 20 **The percentage accuracy scores for each of the individual datasets and individual classification methods where the top 20 genes are used and n = 5 samples per class**. The percentage accuracy of the top 20 genes, selected by the feature selection methods, to form classifiers which can predict the class of blind test data for each of the 9 datasets. These figures show the results for each of the classification methods when a reduced training set of 10 (5 from each class) is used. Click here for file

Additional File 21 **The percentage accuracy scores for each of the individual datasets and individual classification methods where the top 20 genes are used and n = 10 samples per class**. The percentage accuracy of the top 20 genes, selected by the feature selection methods, to form classifiers which can predict the class of blind test data for each of the 9 datasets. These figures show the results for each of the classification methods when a reduced training set of 20 (10 from each class) is used. Click here for file

Additional File 22 **The percentage accuracy scores for each of the individual datasets and individual classification methods where the top 20 genes are used and n = 50% of the samples per class**. The percentage accuracy of the top 20 genes, selected by the feature selection methods, to form classifiers which can predict the class of blind test data for each of the 9 datasets. These figures show the results for each of the classification methods when a datasets split equally into training and test sets is used. Click here for file

## Figures and Tables

**Table 1:** Variance Structure of the 9 datasets

**Dataset**	**Reference**	**Classes**	**Pooled Variance**
DLBCL	Shipp et al., 2002	Follicular or Germinal	0.147
Prostate	Singh et al., 2002	Prostate or non-Prostate	0.182
Colon	Alon et al., 1999	Tumour or Normal	0.528
Leukaemia	Golub et al., 1999	ALL or AML	0.458
Myeloma	Tian et al., 2003	Presence or Absence of focal lesions of bone	0.841
ALL.1	Chiaretti et al., 2004	B-cell or T-cell origin	0.204
ALL.2	Chiaretti et al., 2004	With or without MDR	0.221
ALL.3	Chiaretti et al., 2004	Did or did not relapse	0.178
ALL.4	Chiaretti et al., 2004	With or without t(9;22) chromosome translocation	0.159
