# Supplementary material for: Comparison and evaluation of methods for generating differentially expressed gene lists from microarray data
Source: BMC Bioinformatics. 2006 Jul 26;7:359. doi: 10.1186/1471-2105-7-359 (PMC1544358; doi:10.1186/1471-2105-7-359)
Supplement: Additional File 1 — Overlap in gene lists produced by different feature selection methods where n = 5 samples per class. Each feature selection method was applied to datasets containing 5 samples per class. The overlap of genes ranked in the top 100 by each method was compared using a binary distance metric. Dendrograms show the results of average linkage hierarchical cluster analysis of these scores for each dataset. Percentage matricies below each of the dendrograms show the percentage similarity between each of the feature selection methods. [file 1471-2105-7-359-S1.pdf]

ALL.1, where n = 5

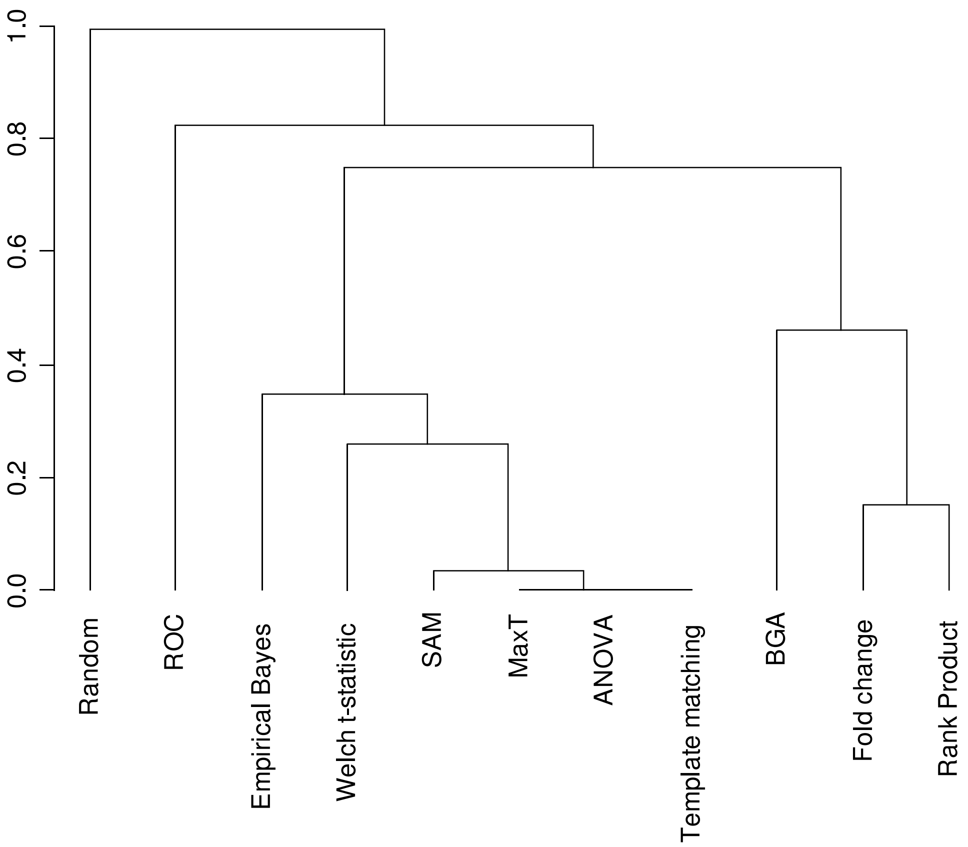

	BGA	SAM	ANOVA	Template matching	Welch t-statistic	Fold change	Empirical Bayes	MaxT	ROC	Rank Product
BGA	/	36	35.1	35.1	31.5	71	42.7	35.1	18.1	68.8
SAM	36	/	98.2	98.2	84.8	40.4	81.3	98.2	35	43.9
ANOVA	35.1	98.2	/	100	85.3	39.3	79.6	100	35.1	42.8
Template matching	35.1	98.2	100	/	85.3	39.3	79.6	100	35.1	42.8
Welch t-statistic	31.5	84.8	85.3	85.3	/	36.1	74.3	85.3	34.7	38.9
Fold change	71	40.4	39.3	39.3	36.1	/	48.3	39.3	19.9	91.9
Empirical Bayes	42.7	81.3	79.6	79.6	74.3	48.3	/	79.6	34	52.8
MaxT	35.1	98.2	100	100	85.3	39.3	79.6	/	35.1	42.8
ROC	18.1	35	35.1	35.1	34.7	19.9	34	35.1	/	21.2
Rank Product	68.8	43.9	42.8	42.8	38.9	91.9	52.8	42.8	21.2	/
Random	0.6	1.4	1.4	1.4	1.3	0.5	1.1	1.4	0.8	0.7

ALL.2, where n = 5

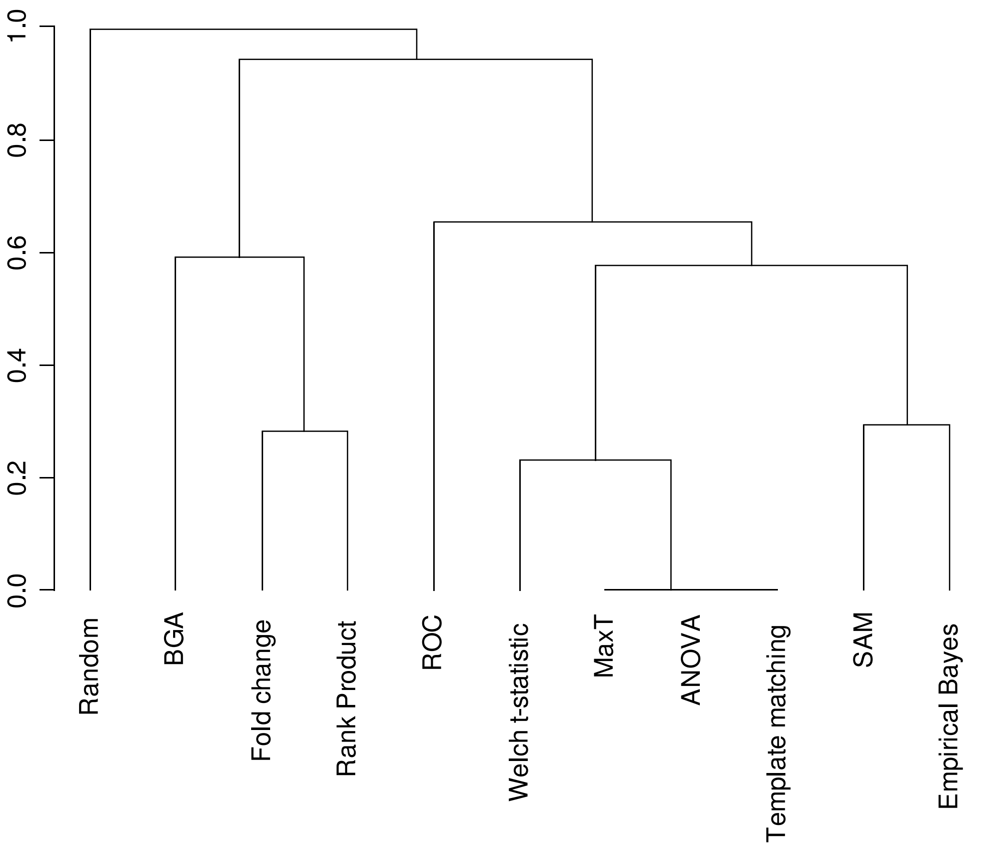

	BGA	SAM	ANOVA	Template matching	Welch t-statistic	Fold change	Empirical Bayes	MaxT	ROC	Rank Product
BGA	/	19.4	8.2	8.2	6.5	60.7	17.6	8.2	8.2	55.2
SAM	19.4	/	63	63	60.5	19.8	82.7	63	45.5	20.1
ANOVA	8.2	63	/	100	87	7.9	57.2	100	55.5	8.1
Template matching	8.2	63	100	/	87	7.9	57.2	100	55.5	8.1
Welch t-statistic	6.5	60.5	87	87	/	6.5	54.8	87	51.8	6.9
Fold change	60.7	19.8	7.9	7.9	6.5	/	17.4	7.9	7.4	83.5
Empirical Bayes	17.6	82.7	57.2	57.2	54.8	17.4	/	57.2	43.3	17.6
MaxT	8.2	63	100	100	87	7.9	57.2	/	55.5	8.1
ROC	8.2	45.5	55.5	55.5	51.8	7.4	43.3	55.5	/	7.6
Rank Product	55.2	20.1	8.1	8.1	6.9	83.5	17.6	8.1	7.6	/
Random	0.8	0.8	0.6	0.6	0.8	0.6	0.9	0.6	0.5	0.6

ALL.3, where n = 5

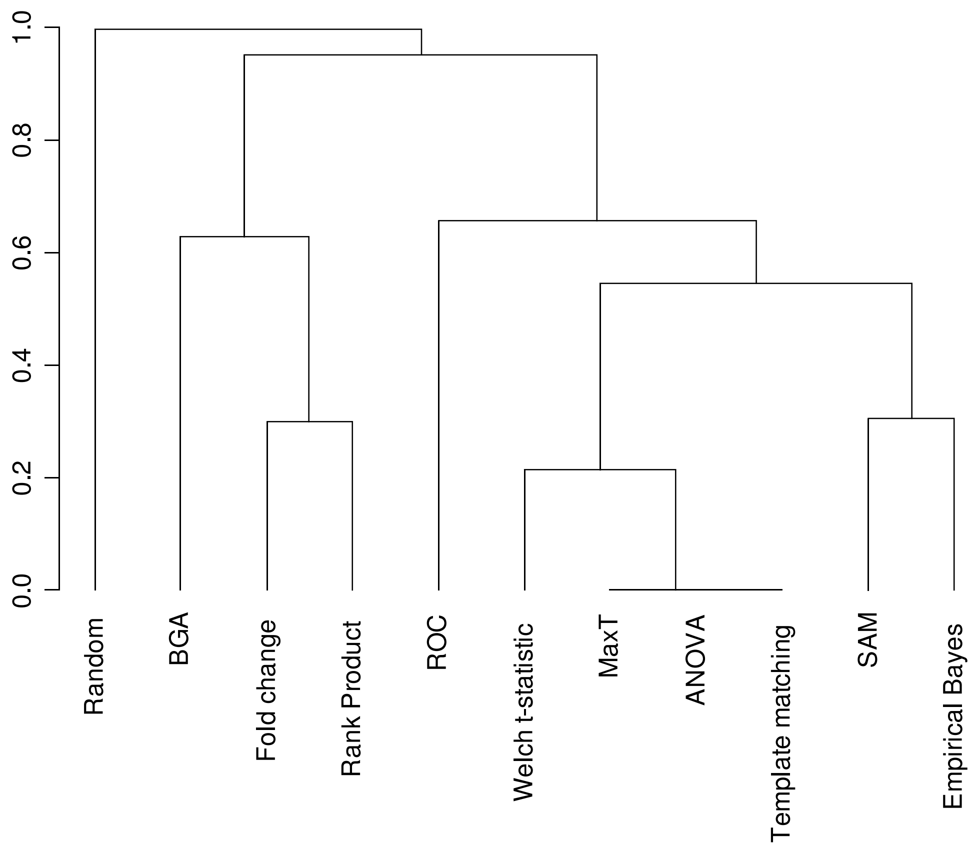

	BGA	SAM	ANOVA	Template matching	Welch t-statistic	Fold change	Empirical Bayes	MaxT	ROC	Rank Product
BGA	/	13.4	5.6	5.6	5	57.8	16.4	5.6	5.8	50.2
SAM	13.4	/	69.7	69.7	67.4	17	81.9	69.7	46.7	16
ANOVA	5.6	69.7	/	100	88	7.6	55.7	100	55.5	7.2
Template matching	5.6	69.7	100	/	88	7.6	55.7	100	55.5	7.2
Welch t-statistic	5	67.4	88	88	/	7.5	54.6	88	51.7	7.2
Fold change	57.8	17	7.6	7.6	7.5	/	20.2	7.6	6.6	82.3
Empirical Bayes	16.4	81.9	55.7	55.7	54.6	20.2	/	55.7	40.1	18.9
MaxT	5.6	69.7	100	100	88	7.6	55.7	/	55.5	7.2
ROC	5.8	46.7	55.5	55.5	51.7	6.6	40.1	55.5	/	6.2
Rank Product	50.2	16	7.2	7.2	7.2	82.3	18.9	7.2	6.2	/
Random	0.8	0.6	0.4	0.4	0.5	0.8	0.5	0.4	0.8	0.8

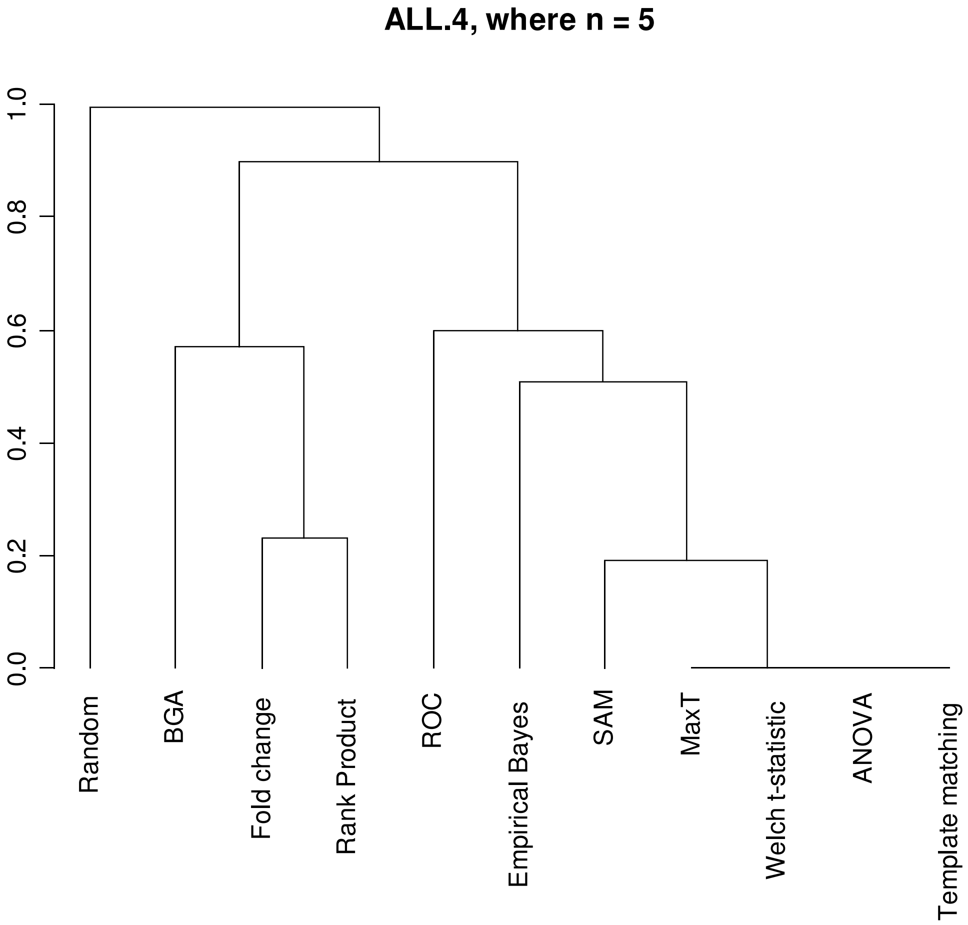

	BGA	SAM	ANOVA	Template matching	Welch t-statistic	Fold change	Empirical Bayes	MaxT	ROC	Rank Product
BGA	/	17.4	13.7	13.7	13.7	61.4	24.8	13.7	13.9	58.8
SAM	17.4	/	89.4	89.4	89.4	21.5	74.2	89.4	57.3	21.5
ANOVA	13.7	89.4	/	100	100	17.6	63.6	100	59.2	17.8
Template matching	13.7	89.4	100	/	100	17.6	63.6	100	59.2	17.8
Welch t-statistic	13.7	89.4	100	100	/	17.6	63.6	100	59.2	17.8
Fold change	61.4	21.5	17.6	17.6	17.6	/	31.4	17.6	14.6	87
Empirical Bayes	24.8	74.2	63.6	63.6	63.6	31.4	/	63.6	47.9	31
MaxT	13.7	89.4	100	100	100	17.6	63.6	/	59.2	17.8
ROC	13.9	57.3	59.2	59.2	59.2	14.6	47.9	59.2	/	14.4
Rank Product	58.8	21.5	17.8	17.8	17.8	87	31	17.8	14.4	/
Random	0.6	0.7	0.6	0.6	0.6	0.9	0.9	0.6	1.3	0.9

Colon, where n = 5

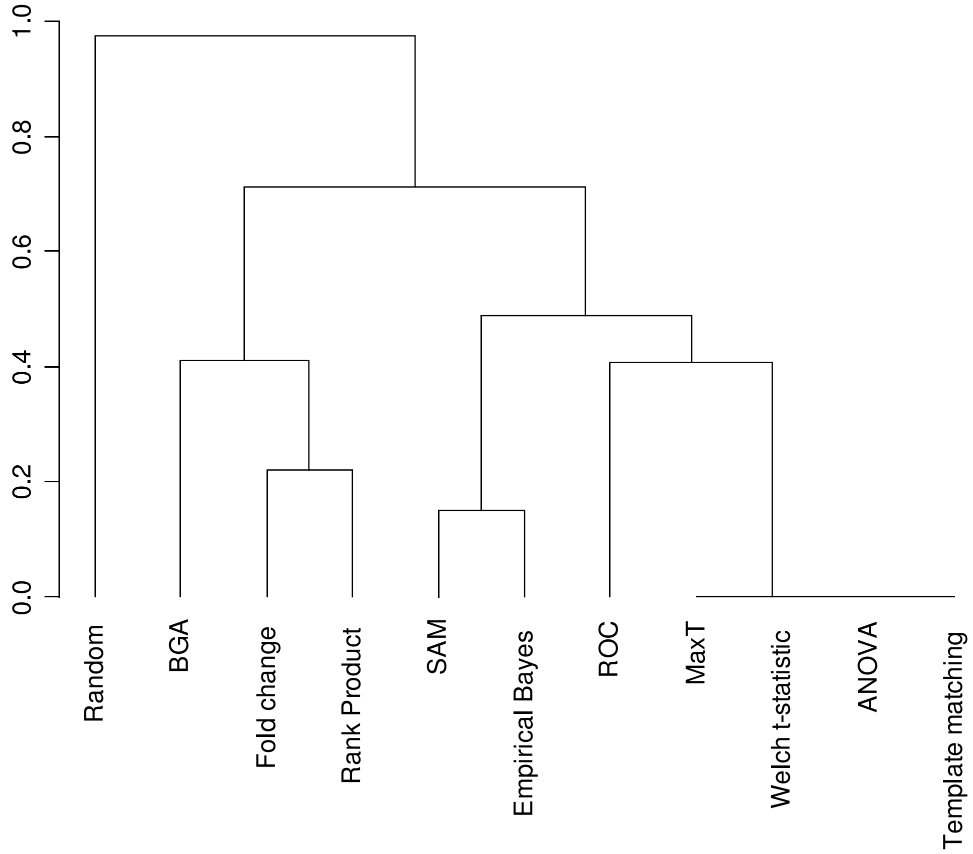

	BGA	SAM	ANOVA	Template matching	Welch t-statistic	Fold change	Empirical Bayes	MaxT	ROC	Rank Product
BGA	/	57.7	37.7	37.7	37.7	75.7	59.2	37.7	37.6	72.5
SAM	57.7	/	71	71	71	65.1	91.8	71	63	56.4
ANOVA	37.7	71	/	100	100	40.4	67.1	100	74.3	33.9
Template matching	37.7	71	100	/	100	40.4	67.1	100	74.3	33.9
Welch t-statistic	37.7	71	100	100	/	40.4	67.1	100	74.3	33.9
Fold change	75.7	65.1	40.4	40.4	40.4	/	66.5	40.4	39.1	87.5
Empirical Bayes	59.2	91.8	67.1	67.1	67.1	66.5	/	67.1	60.6	57.1
MaxT	37.7	71	100	100	100	40.4	67.1	/	74.3	33.9
ROC	37.6	63	74.3	74.3	74.3	39.1	60.6	74.3	/	33
Rank Product	72.5	56.4	33.9	33.9	33.9	87.5	57.1	33.9	33	/
Random	4.8	5	4.4	4.4	4.4	5.1	4.9	4.4	4.7	5.3

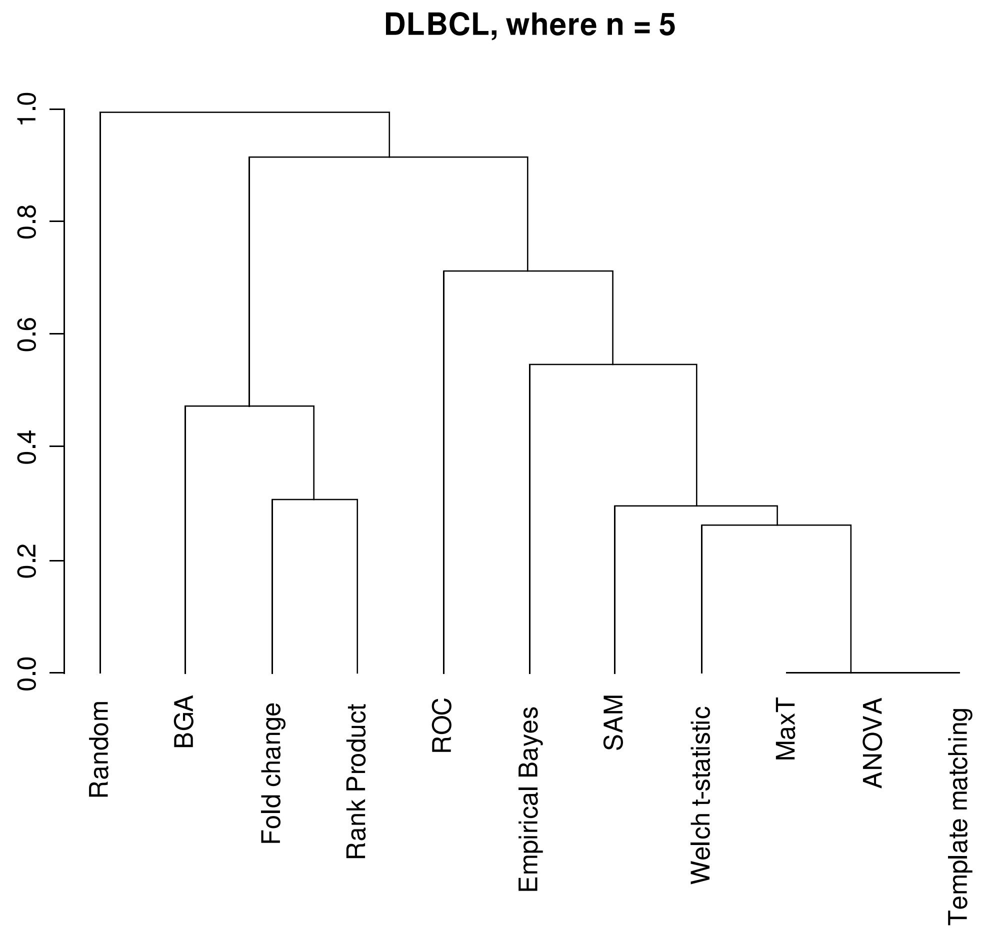

	BGA	SAM	ANOVA	Template matching	Welch t-statistic	Fold change	Empirical Bayes	MaxT	ROC	Rank Product
BGA	/	18.2	11.9	11.9	10.4	70.9	27.7	11.9	10.4	67.3
SAM	18.2	/	84.5	84.5	76.3	19.7	74.3	84.5	44.4	21.3
ANOVA	11.9	84.5	/	100	84.9	13.2	59.8	100	47.7	15
Template matching	11.9	84.5	100	/	84.9	13.2	59.8	100	47.7	15
Welch t-statistic	10.4	76.3	84.9	84.9	/	11.9	57.2	84.9	43.8	13.5
Fold change	70.9	19.7	13.2	13.2	11.9	/	29.5	13.2	10.1	81.8
Empirical Bayes	27.7	74.3	59.8	59.8	57.2	29.5	/	59.8	35.8	31.1
MaxT	11.9	84.5	100	100	84.9	13.2	59.8	/	47.7	15
ROC	10.4	44.4	47.7	47.7	43.8	10.1	35.8	47.7	/	10.5
Rank Product	67.3	21.3	15	15	13.5	81.8	31.1	15	10.5	/
Random	1	1.1	1.1	1.1	1	1.2	1	1.1	2.1	1

Leukeamia, where n = 5

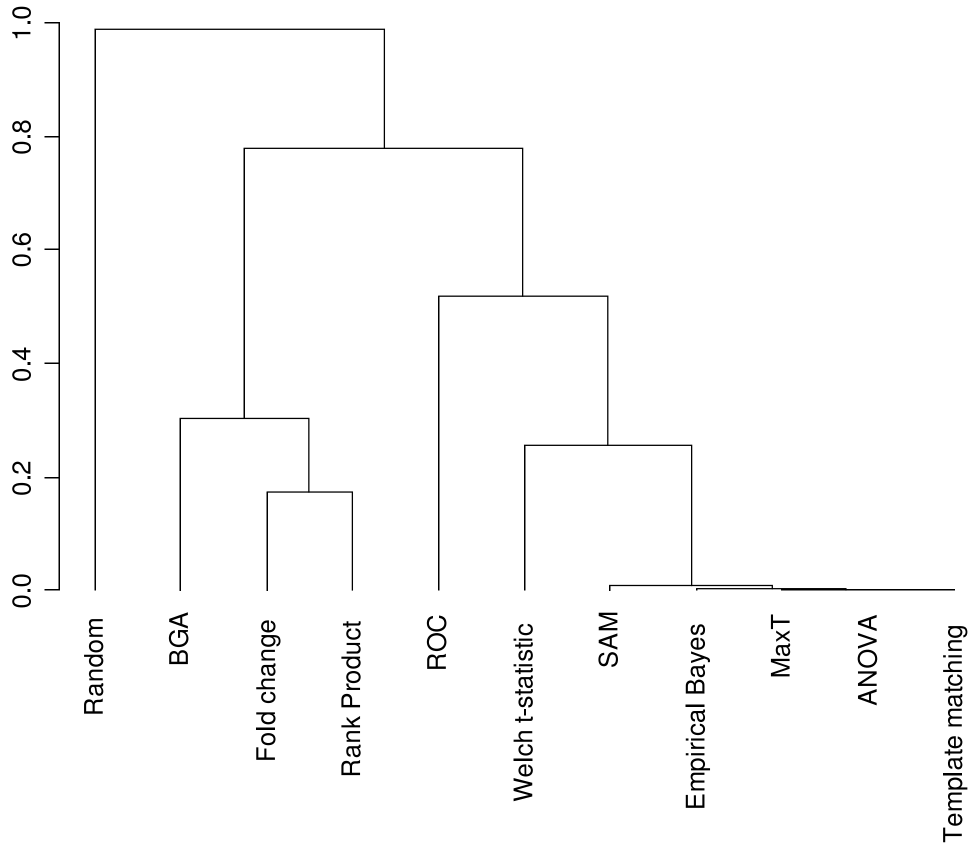

	BGA	SAM	ANOVA	Template matching	Welch t-statistic	Fold change	Empirical Bayes	MaxT	ROC	Rank Product
BGA	/	36.7	36.3	36.3	33.7	83	36.5	36.3	27.6	81.1
SAM	36.7	/	99.5	99.5	85.3	37.6	99.7	99.5	65.3	38.4
ANOVA	36.3	99.5	/	100	85.4	37.2	99.8	100	65.4	38
Template matching	36.3	99.5	100	/	85.4	37.2	99.8	100	65.4	38
Welch t-statistic	33.7	85.3	85.4	85.4	/	35.8	85.3	85.4	63	35.9
Fold change	83	37.6	37.2	37.2	35.8	/	37.4	37.2	30.1	90.6
Empirical Bayes	36.5	99.7	99.8	99.8	85.3	37.4	/	99.8	65.3	38.2
MaxT	36.3	99.5	100	100	85.4	37.2	99.8	/	65.4	38
ROC	27.6	65.3	65.4	65.4	63	30.1	65.3	65.4	/	31.3
Rank Product	81.1	38.4	38	38	35.9	90.6	38.2	38	31.3	/
Random	1.4	2.2	2.2	2.2	2.2	1.6	2.2	2.2	1.3	1.6

Myeloma, where n = 5

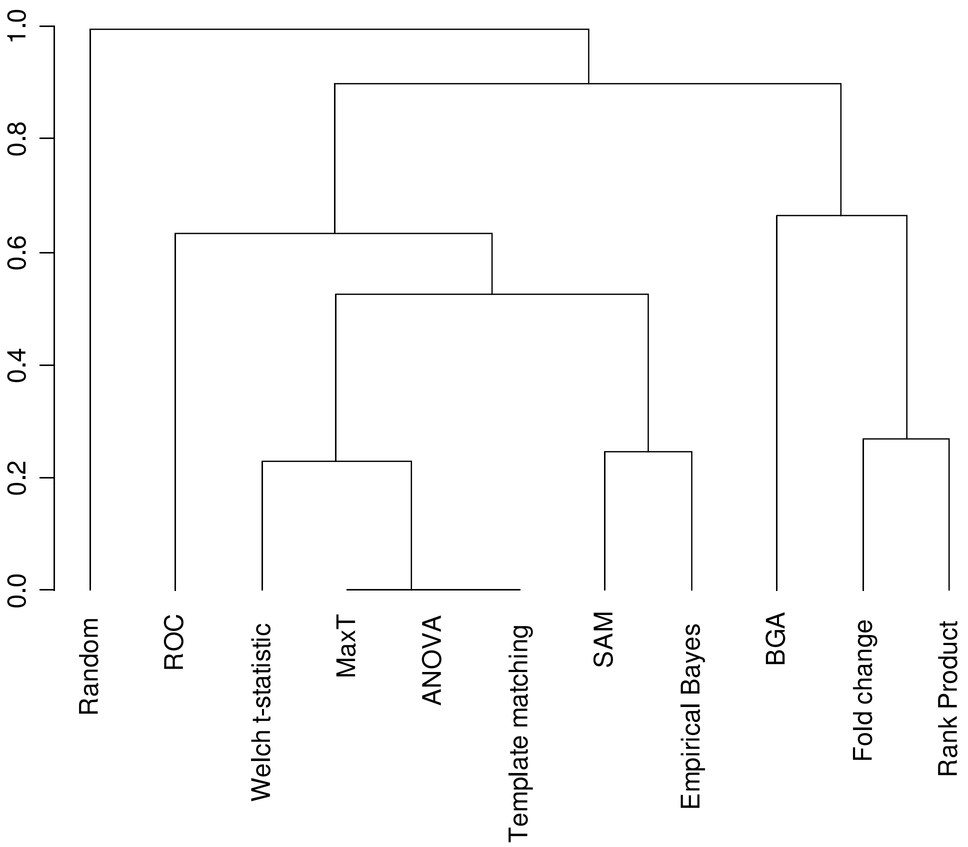

	BGA	SAM	ANOVA	Template matching	Welch t-statistic	Fold change	Empirical Bayes	MaxT	ROC	Rank Product
BGA	/	23.4	13.5	13.5	12.6	52.2	24.9	13.5	9.8	48.3
SAM	23.4	/	69.9	69.9	65.4	31.9	86	69.9	47.3	24.6
ANOVA	13.5	69.9	/	100	87.2	18.1	60.7	100	58.8	14.5
Template matching	13.5	69.9	100	/	87.2	18.1	60.7	100	58.8	14.5
Welch t-statistic	12.6	65.4	87.2	87.2	/	16.9	57.4	87.2	54.7	13.5
Fold change	52.2	31.9	18.1	18.1	16.9	/	32.8	18.1	14.3	84.5
Empirical Bayes	24.9	86	60.7	60.7	57.4	32.8	/	60.7	42.4	25.8
MaxT	13.5	69.9	100	100	87.2	18.1	60.7	/	58.8	14.5
ROC	9.8	47.3	58.8	58.8	54.7	14.3	42.4	58.8	/	11
Rank Product	48.3	24.6	14.5	14.5	13.5	84.5	25.8	14.5	11	/
Random	0.7	0.5	0.7	0.7	0.4	1.1	0.6	0.7	1	1.3

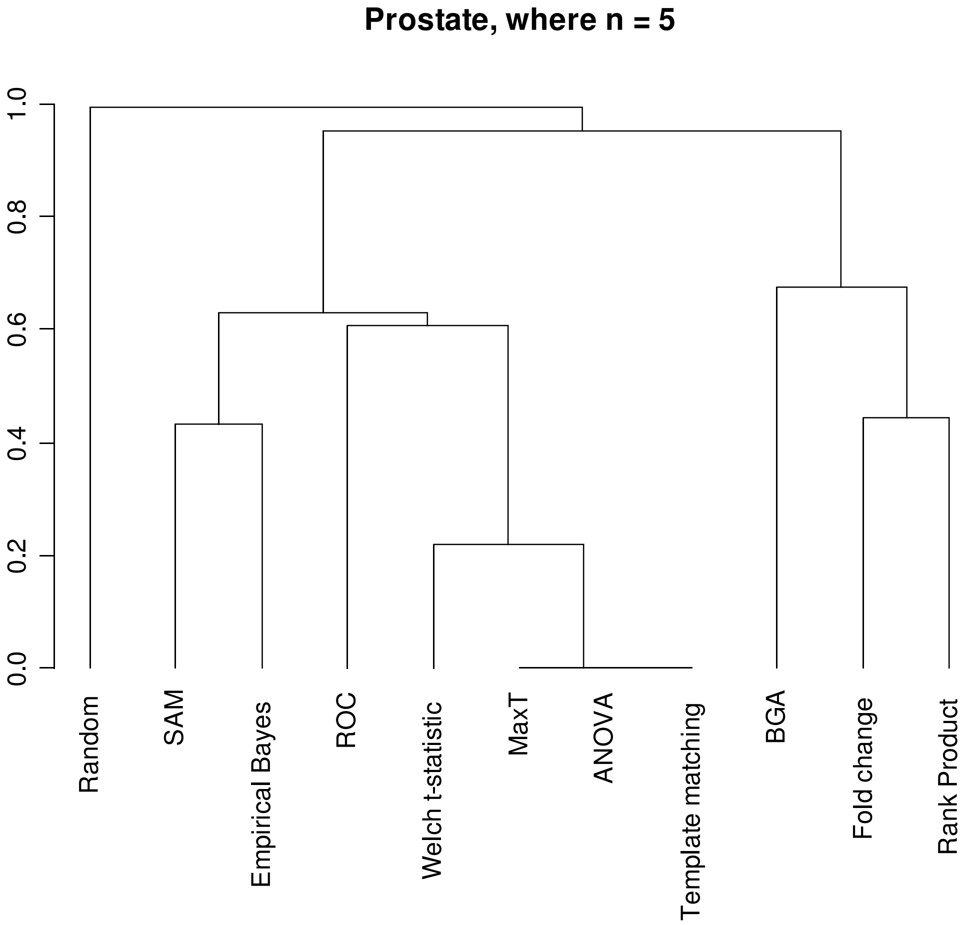

	BGA	SAM	ANOVA	Template matching	Welch t-statistic	Fold change	Empirical Bayes	MaxT	ROC	Rank Product
BGA	/	24.8	6.4	6.4	5.8	49.2	18	6.4	7.7	49
SAM	24.8	/	57.6	57.6	52.7	19.7	72.4	57.6	41.3	22.3
ANOVA	6.4	57.6	/	100	87.7	3.8	57.3	100	57.4	5.1
Template matching	6.4	57.6	100	/	87.7	3.8	57.3	100	57.4	5.1
Welch t-statistic	5.8	52.7	87.7	87.7	/	3.2	53.9	87.7	54	4.5
Fold change	49.2	19.7	3.8	3.8	3.2	/	10.8	3.8	3.7	71.5
Empirical Bayes	18	72.4	57.3	57.3	53.9	10.8	/	57.3	45.9	13.8
MaxT	6.4	57.6	100	100	87.7	3.8	57.3	/	57.4	5.1
ROC	7.7	41.3	57.4	57.4	54	3.7	45.9	57.4	/	4.7
Rank Product	49	22.3	5.1	5.1	4.5	71.5	13.8	5.1	4.7	/
Random	0.8	1.1	0.9	0.9	0.9	1.1	1	0.9	1	1.2
