# Supplementary material for: Comparison and evaluation of methods for generating differentially expressed gene lists from microarray data
Source: BMC Bioinformatics. 2006 Jul 26;7:359. doi: 10.1186/1471-2105-7-359 (PMC1544358; doi:10.1186/1471-2105-7-359)
Supplement: Additional File 2 — Overlap in gene lists produced by different feature selection methods where n = 10 samples per class. Each feature selection method was applied to datasets containing 10 samples per class. The overlap of genes ranked in the top 100 by each method was compared using a binary distance metric. Dendrograms show the results of average linkage hierarchical cluster analysis of these scores for each dataset. Percentage matricies below each of the dendrograms show the percentage similarity between each of the feature selection methods. [file 1471-2105-7-359-S2.pdf]

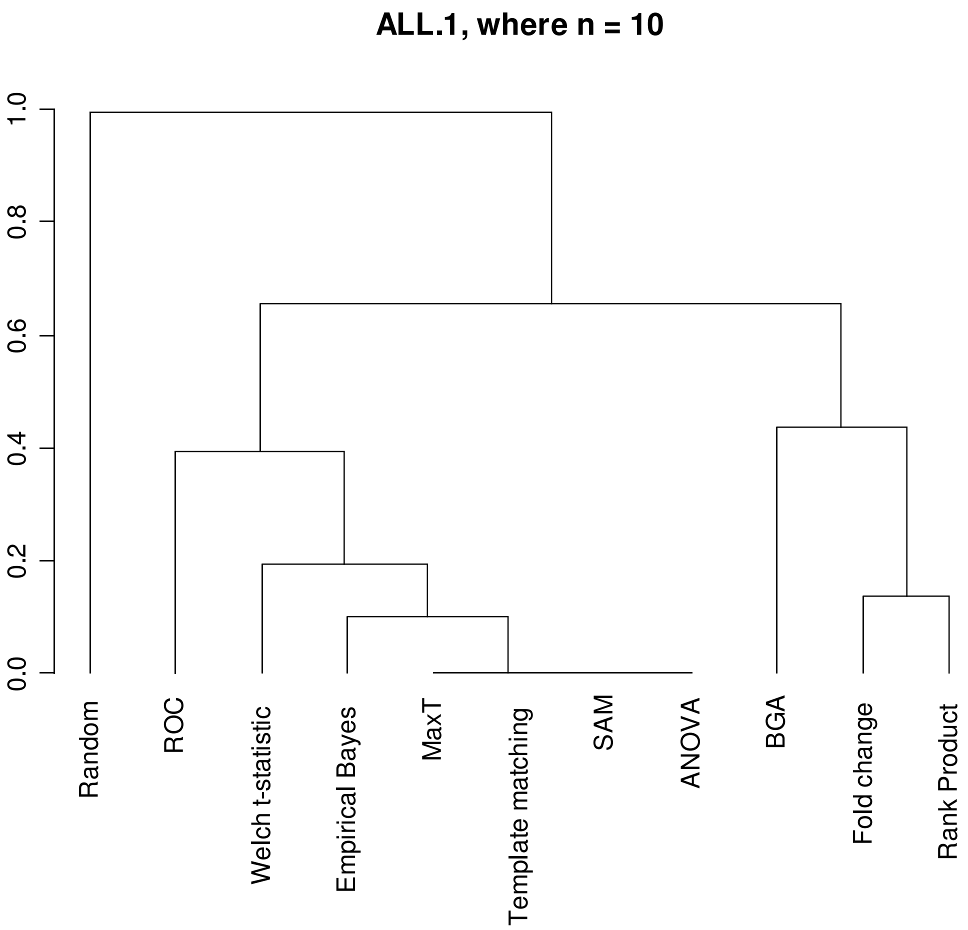

	BGA	SAM	ANOVA	Template matching	Welch t-statistic	Fold change	Empirical Bayes	MaxT	ROC	Rank Product
BGA	/	46.6	46.6	46.6	43.6	73	48.4	46.6	42.2	71.1
SAM	46.6	/	100	100	89.6	52.9	94.8	100	76.7	56.2
ANOVA	46.6	100	/	100	89.6	52.9	94.8	100	76.7	56.2
Template matching	46.6	100	100	/	89.6	52.9	94.8	100	76.7	56.2
Welch t-statistic	43.6	89.6	89.6	89.6	/	50.5	87.8	89.6	71.5	53.5
Fold change	73	52.9	52.9	52.9	50.5	/	55.4	52.9	45.6	92.7
Empirical Bayes	48.4	94.8	94.8	94.8	87.8	55.4	/	94.8	75.1	58.9
MaxT	46.6	100	100	100	89.6	52.9	94.8	/	76.7	56.2
ROC	42.2	76.7	76.7	76.7	71.5	45.6	75.1	76.7	/	48
Rank Product	71.1	56.2	56.2	56.2	53.5	92.7	58.9	56.2	48	/
Random	0.9	0.7	0.7	0.7	0.6	0.8	0.7	0.7	0.8	0.8

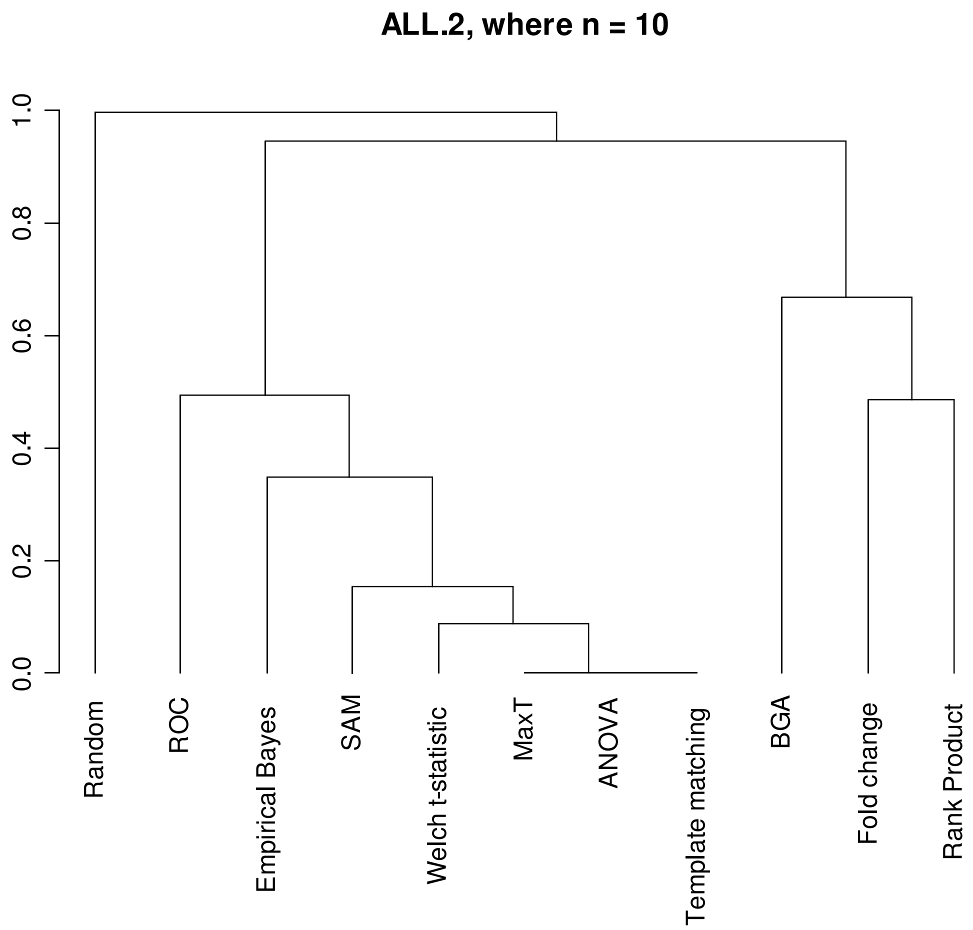

	BGA	SAM	ANOVA	Template matching	Welch t-statistic	Fold change	Empirical Bayes	MaxT	ROC	Rank Product
BGA	/	12.7	10.3	10.3	9.5	56.2	15.9	10.3	8.1	42.7
SAM	12.7	/	92.4	92.4	89.1	14.8	80.4	92.4	66.5	8.9
ANOVA	10.3	92.4	/	100	95.3	11.7	78.7	100	69.1	7.3
Template matching	10.3	92.4	100	/	95.3	11.7	78.7	100	69.1	7.3
Welch t-statistic	9.5	89.1	95.3	95.3	/	11.9	78.4	95.3	67.4	7
Fold change	56.2	14.8	11.7	11.7	11.9	/	17.9	11.7	9.4	67.9
Empirical Bayes	15.9	80.4	78.7	78.7	78.4	17.9	/	78.7	62.2	11.4
MaxT	10.3	92.4	100	100	95.3	11.7	78.7	/	69.1	7.3
ROC	8.1	66.5	69.1	69.1	67.4	9.4	62.2	69.1	/	6.1
Rank Product	42.7	8.9	7.3	7.3	7	67.9	11.4	7.3	6.1	/
Random	0.3	0.7	0.7	0.7	0.7	0.5	0.6	0.7	0.7	0.2

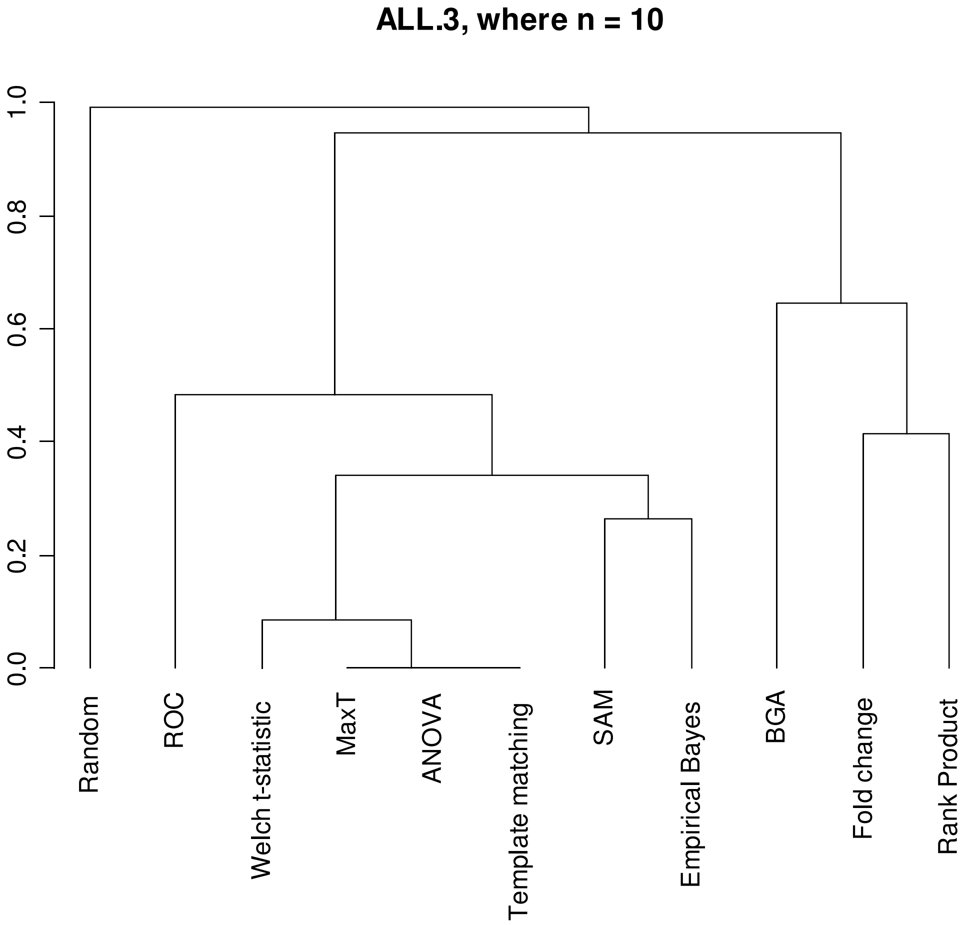

	BGA	SAM	ANOVA	Template matching	Welch t-statistic	Fold change	Empirical Bayes	MaxT	ROC	Rank Product
BGA	/	15.2	8.7	8.7	8.4	57.8	15.1	8.7	9	46.1
SAM	15.2	/	82	82	79.3	17.3	84.8	82	63.5	11.8
ANOVA	8.7	82	/	100	95.5	10.2	77.8	100	70.2	7
Template matching	8.7	82	100	/	95.5	10.2	77.8	100	70.2	7
Welch t-statistic	8.4	79.3	95.5	95.5	/	10.1	76.7	95.5	70	6.8
Fold change	57.8	17.3	10.2	10.2	10.1	/	16.4	10.2	9.5	73.8
Empirical Bayes	15.1	84.8	77.8	77.8	76.7	16.4	/	77.8	63.4	11.2
MaxT	8.7	82	100	100	95.5	10.2	77.8	/	70.2	7
ROC	9	63.5	70.2	70.2	70	9.5	63.4	70.2	/	6.3
Rank Product	46.1	11.8	7	7	6.8	73.8	11.2	7	6.3	/
Random	1.4	1.4	1	1	1	1.7	1.5	1	0.7	2.1

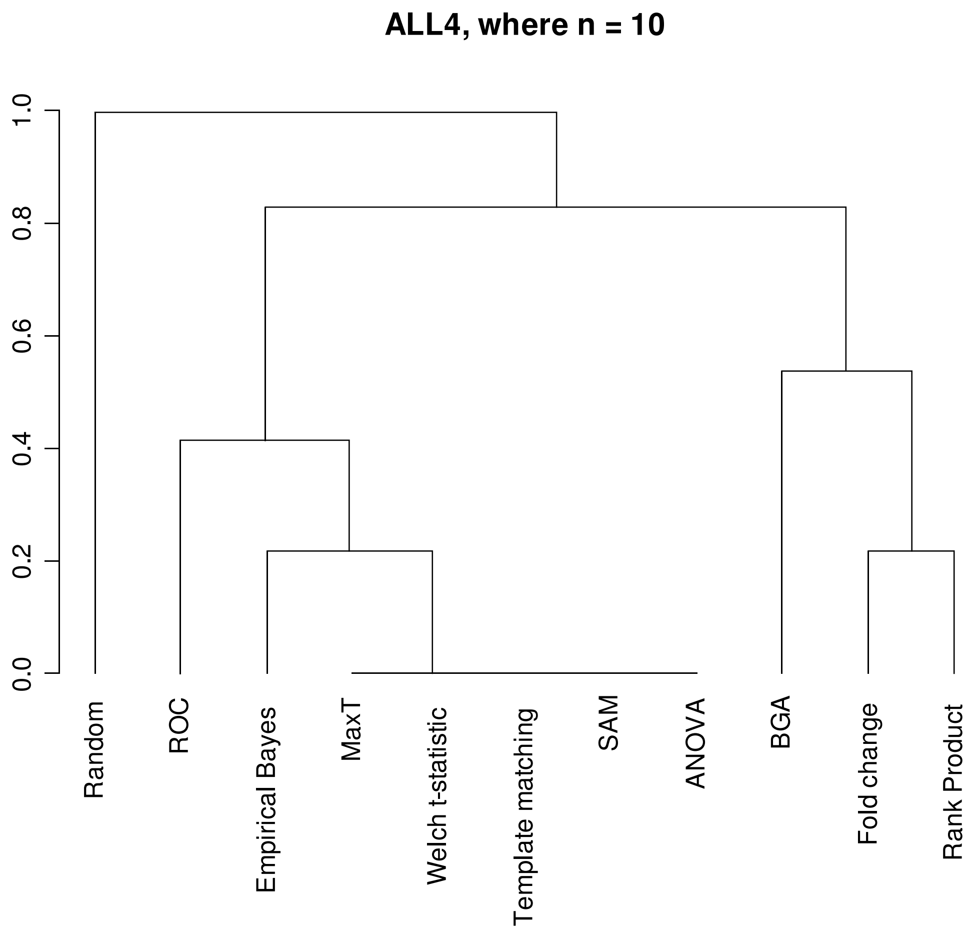

	BGA	SAM	ANOVA	Template matching	Welch t-statistic	Fold change	Empirical Bayes	MaxT	ROC	Rank Product
BGA	/	26.1	26.1	26.1	26.1	64.9	31.3	26.1	25.3	61.9
SAM	26.1	/	100	100	100	30.9	87.8	100	74.4	29.6
ANOVA	26.1	100	/	100	100	30.9	87.8	100	74.4	29.6
Template matching	26.1	100	100	/	100	30.9	87.8	100	74.4	29.6
Welch t-statistic	26.1	100	100	100	/	30.9	87.8	100	74.4	29.6
Fold change	64.9	30.9	30.9	30.9	30.9	/	36.7	30.9	28.6	87.9
Empirical Bayes	31.3	87.8	87.8	87.8	87.8	36.7	/	87.8	71	34.7
MaxT	26.1	100	100	100	100	30.9	87.8	/	74.4	29.6
ROC	25.3	74.4	74.4	74.4	74.4	28.6	71	74.4	/	27.3
Rank Product	61.9	29.6	29.6	29.6	29.6	87.9	34.7	29.6	27.3	/
Random	0.6	0.6	0.6	0.6	0.6	0.4	0.5	0.6	0.8	0.5

Colon, where n = 10

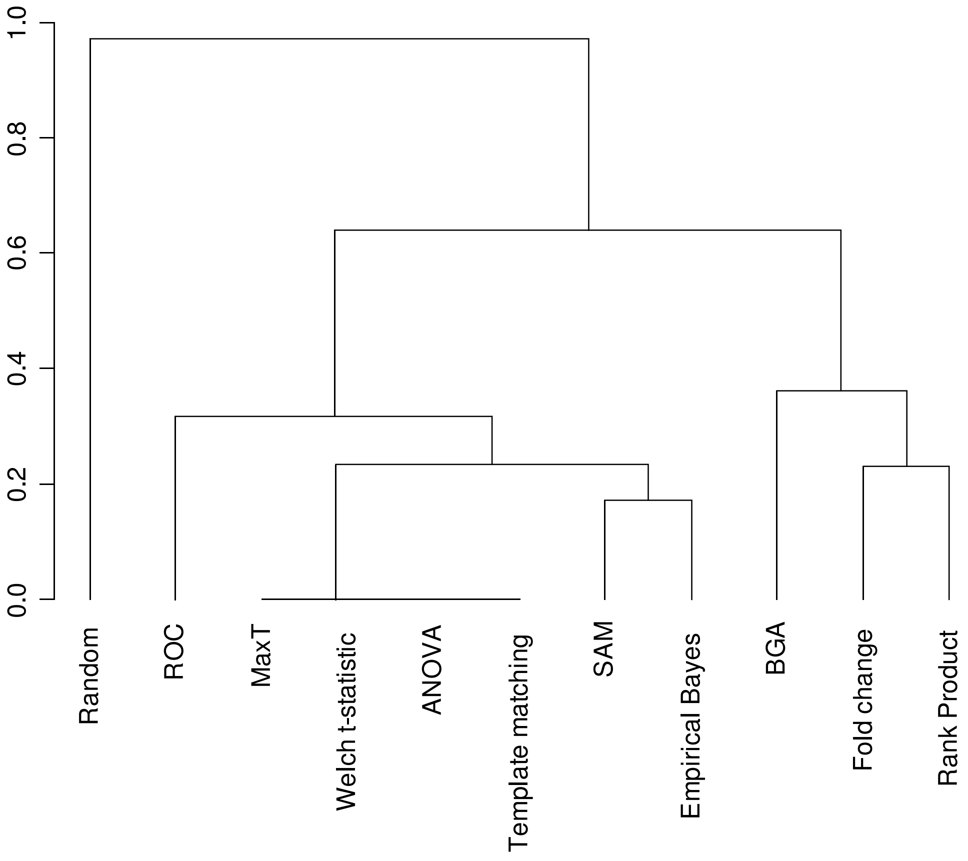

	BGA	SAM	ANOVA	Template matching	Welch t-statistic	Fold change	Empirical Bayes	MaxT	ROC	Rank Product
BGA	/	58.7	50.2	50.2	50.2	80	62.7	50.2	49.4	75.9
SAM	58.7	/	89.2	89.2	89.2	63.7	90.5	89.2	77.8	53.4
ANOVA	50.2	89.2	/	100	100	53.8	84.1	100	83	44.4
Template matching	50.2	89.2	100	/	100	53.8	84.1	100	83	44.4
Welch t-statistic	50.2	89.2	100	100	/	53.8	84.1	100	83	44.4
Fold change	80	63.7	53.8	53.8	53.8	/	67.4	53.8	52.7	87
Empirical Bayes	62.7	90.5	84.1	84.1	84.1	67.4	/	84.1	77.1	57
MaxT	50.2	89.2	100	100	100	53.8	84.1	/	83	44.4
ROC	49.4	77.8	83	83	83	52.7	77.1	83	/	43.5
Rank Product	75.9	53.4	44.4	44.4	44.4	87	57	44.4	43.5	/
Random	5.7	5.3	5.4	5.4	5.4	5.3	5.7	5.4	5.2	5.2

DLBCL, where n = 10

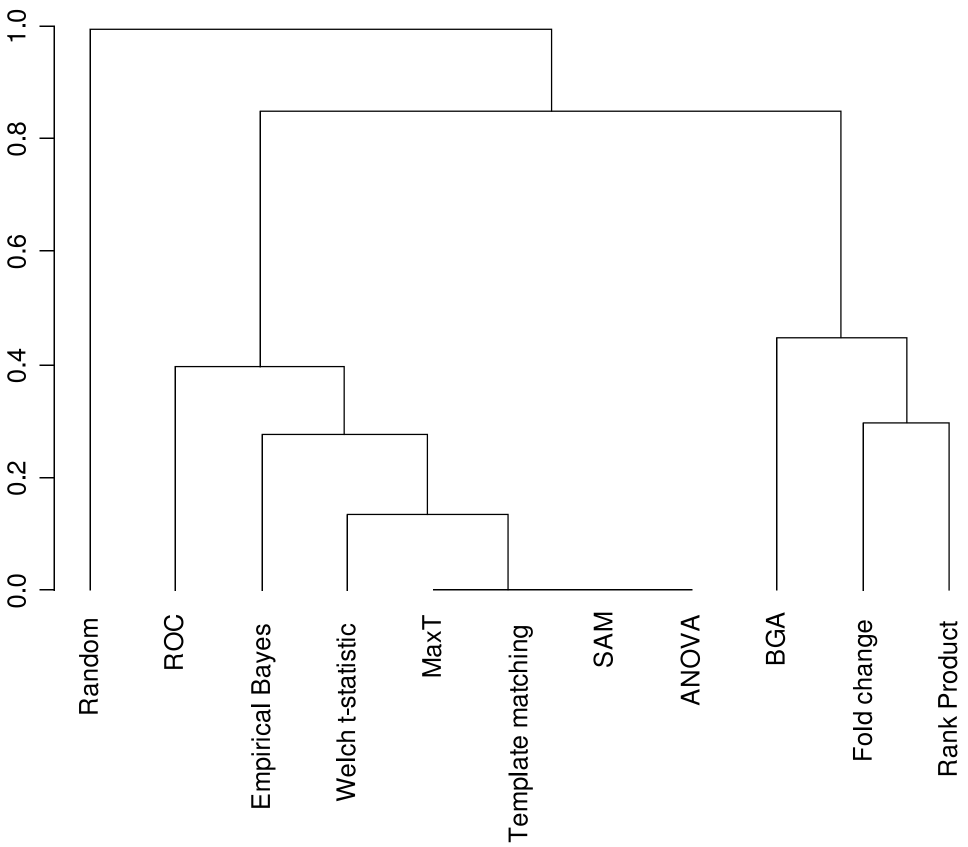

	BGA	SAM	ANOVA	Template matching	Welch t-statistic	Fold change	Empirical Bayes	MaxT	ROC	Rank Product
BGA	/	23.8	23.8	23.8	23.5	72.9	31.4	23.8	22.8	69.4
SAM	23.8	/	100	100	92.8	27.1	84.4	100	76.6	25.5
ANOVA	23.8	100	/	100	92.8	27.1	84.4	100	76.6	25.5
Template matching	23.8	100	100	/	92.8	27.1	84.4	100	76.6	25.5
Welch t-statistic	23.5	92.8	92.8	92.8	/	27	81.8	92.8	74.3	25.6
Fold change	72.9	27.1	27.1	27.1	27	/	36.3	27.1	25.2	82.7
Empirical Bayes	31.4	84.4	84.4	84.4	81.8	36.3	/	84.4	70.7	34.1
MaxT	23.8	100	100	100	92.8	27.1	84.4	/	76.6	25.5
ROC	22.8	76.6	76.6	76.6	74.3	25.2	70.7	76.6	/	23.5
Rank Product	69.4	25.5	25.5	25.5	25.6	82.7	34.1	25.5	23.5	/
Random	1.8	0.8	0.8	0.8	0.8	1.9	0.7	0.8	1.2	1.8

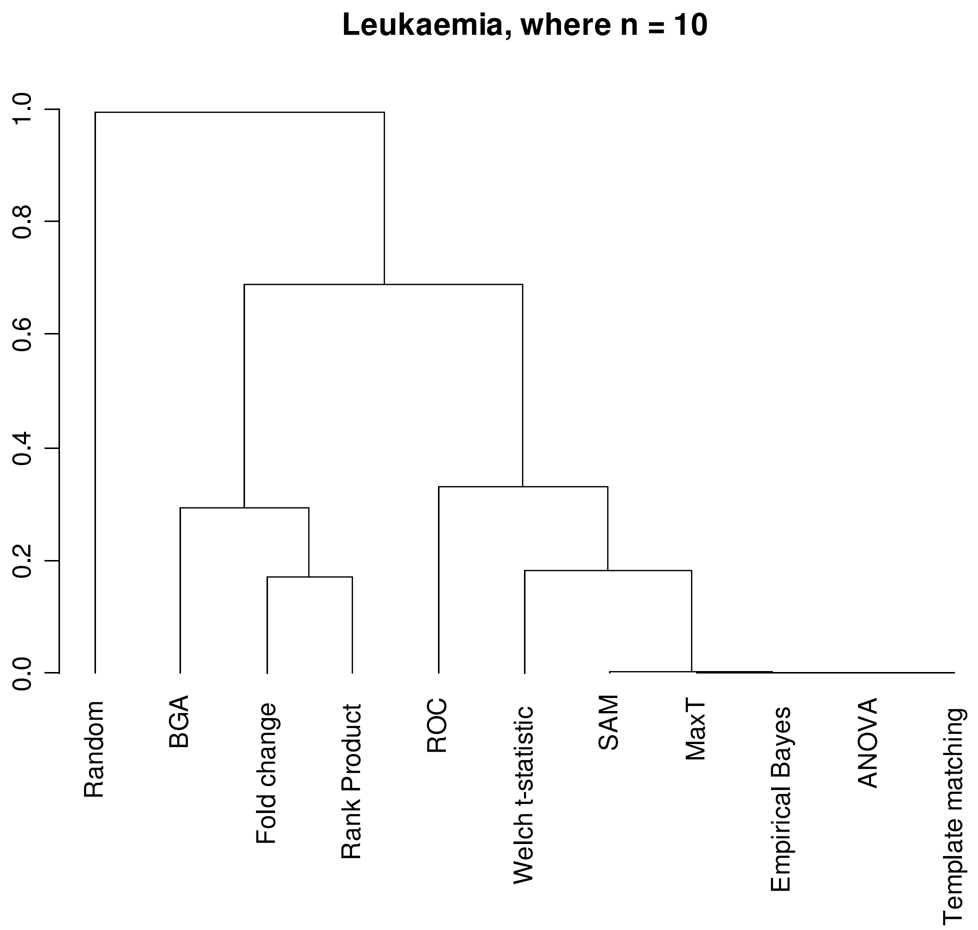

	BGA	SAM	ANOVA	Template matching	Welch t-statistic	Fold change	Empirical Bayes	MaxT	ROC	Rank Product
BGA	/	47.9	47.9	47.9	43.8	83.9	47.9	47.9	41.7	81.7
SAM	47.9	/	99.9	99.9	89.9	49.4	99.9	99.9	80.1	48.3
ANOVA	47.9	99.9	/	100	89.9	49.4	100	100	80.1	48.3
Template matching	47.9	99.9	100	/	89.9	49.4	100	100	80.1	48.3
Welch t-statistic	43.8	89.9	89.9	89.9	/	46.4	89.9	89.9	81.1	46.2
Fold change	83.9	49.4	49.4	49.4	46.4	/	49.4	49.4	43.7	90.7
Empirical Bayes	47.9	99.9	100	100	89.9	49.4	/	100	80.1	48.3
MaxT	47.9	99.9	100	100	89.9	49.4	100	/	80.1	48.3
ROC	41.7	80.1	80.1	80.1	81.1	43.7	80.1	80.1	/	42.9
Rank Product	81.7	48.3	48.3	48.3	46.2	90.7	48.3	48.3	42.9	/
Random	1	0.9	0.9	0.9	0.9	1.2	0.9	0.9	0.9	1.1

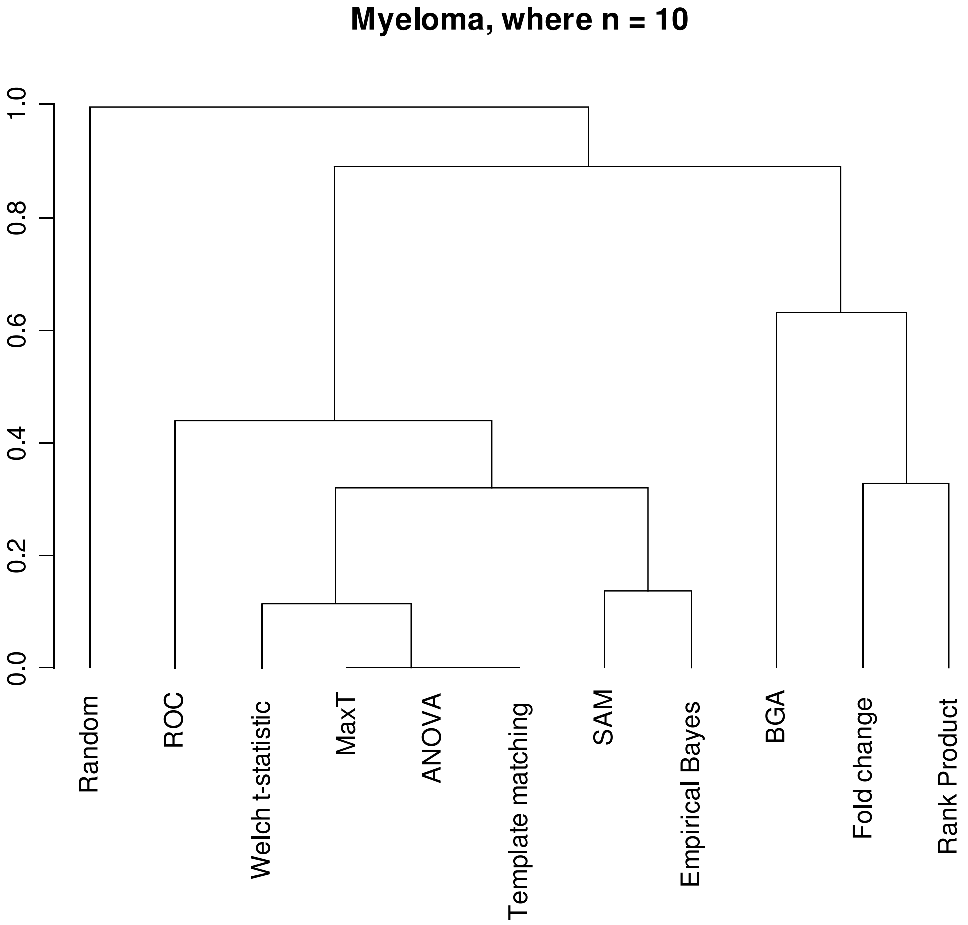

	BGA	SAM	ANOVA	Template matching	Welch t-statistic	Fold change	Empirical Bayes	MaxT	ROC	Rank Product
BGA	/	23.7	17.8	17.8	17	56.2	24.5	17.8	14.9	51.7
SAM	23.7	/	84.1	84.1	81	29	92.7	84.1	68.2	21
ANOVA	17.8	84.1	/	100	94	21.7	79.3	100	74.3	15.5
Template matching	17.8	84.1	100	/	94	21.7	79.3	100	74.3	15.5
Welch t-statistic	17	81	94	94	/	21	76.7	94	73	15
Fold change	56.2	29	21.7	21.7	21	/	29.1	21.7	19.1	80.3
Empirical Bayes	24.5	92.7	79.3	79.3	76.7	29.1	/	79.3	66.2	20.8
MaxT	17.8	84.1	100	100	94	21.7	79.3	/	74.3	15.5
ROC	14.9	68.2	74.3	74.3	73	19.1	66.2	74.3	/	13.4
Rank Product	51.7	21	15.5	15.5	15	80.3	20.8	15.5	13.4	/
Random	1.1	0.7	0.7	0.7	0.6	0.5	0.7	0.7	0.8	0.8

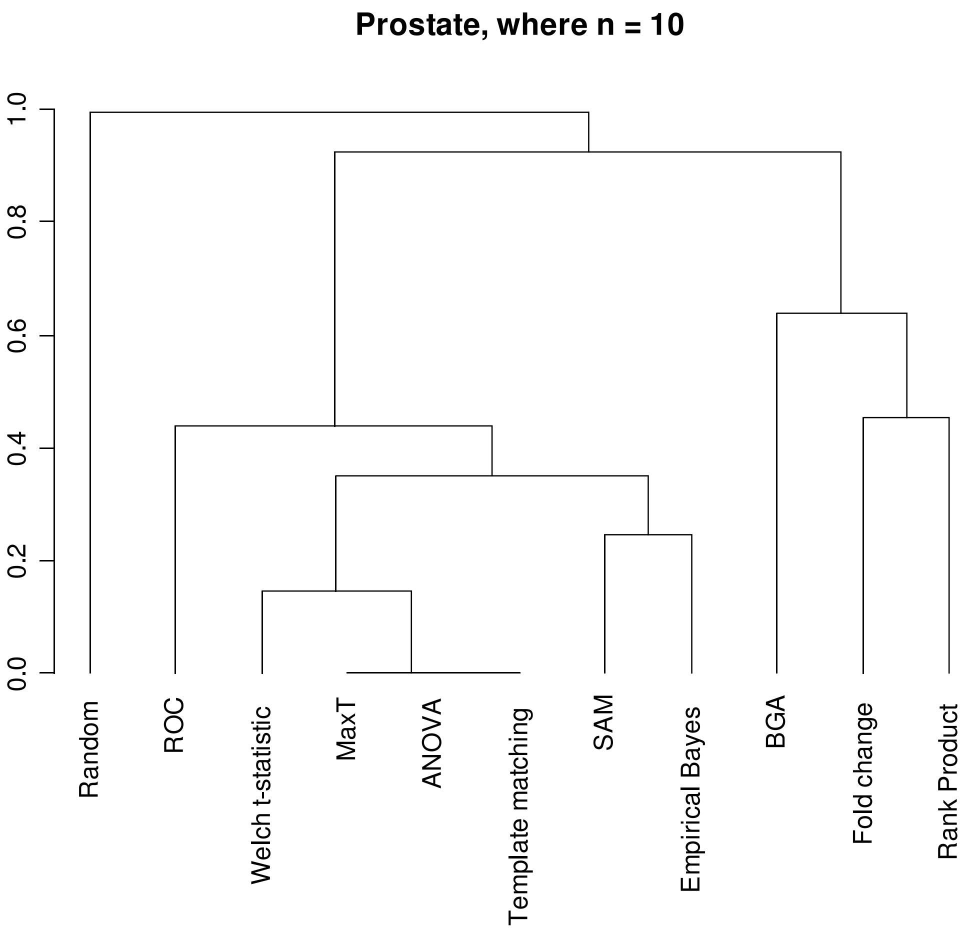

	BGA	SAM	ANOVA	Template matching	Welch t-statistic	Fold change	Empirical Bayes	MaxT	ROC	Rank Product
BGA	/	24.2	14.8	14.8	12.8	52	20.4	14.8	15.3	54.4
SAM	24.2	/	77.8	77.8	73.1	17.5	86.1	77.8	67	21.8
ANOVA	14.8	77.8	/	100	92.2	10.2	81.4	100	74.1	13
Template matching	14.8	77.8	100	/	92.2	10.2	81.4	100	74.1	13
Welch t-statistic	12.8	73.1	92.2	92.2	/	8.9	78.6	92.2	72.1	11.6
Fold change	52	17.5	10.2	10.2	8.9	/	14	10.2	8.5	70.6
Empirical Bayes	20.4	86.1	81.4	81.4	78.6	14	/	81.4	69.7	17.5
MaxT	14.8	77.8	100	100	92.2	10.2	81.4	/	74.1	13
ROC	15.3	67	74.1	74.1	72.1	8.5	69.7	74.1	/	11.1
Rank Product	54.4	21.8	13	13	11.6	70.6	17.5	13	11.1	/
Random	0.8	0.8	0.8	0.8	0.8	1	0.8	0.8	0.6	0.9
