# Supplementary material for: Comparison and evaluation of methods for generating differentially expressed gene lists from microarray data
Source: BMC Bioinformatics. 2006 Jul 26;7:359. doi: 10.1186/1471-2105-7-359 (PMC1544358; doi:10.1186/1471-2105-7-359)
Supplement: Additional File 3 — Overlap in gene lists produced by different feature selection methods where n = 50% of the samples per class. Each feature selection method was applied to datasets containing 50% of the samples per class. The overlap of genes ranked in the top 100 by each method was compared using a binary distance metric. Dendrograms show the results of average linkage hierarchical cluster analysis of these scores for each dataset. Percentage matricies below each of the dendrograms show the percentage similarity between each of the feature selection methods. [file 1471-2105-7-359-S3.pdf]

ALL1, where n = 50%

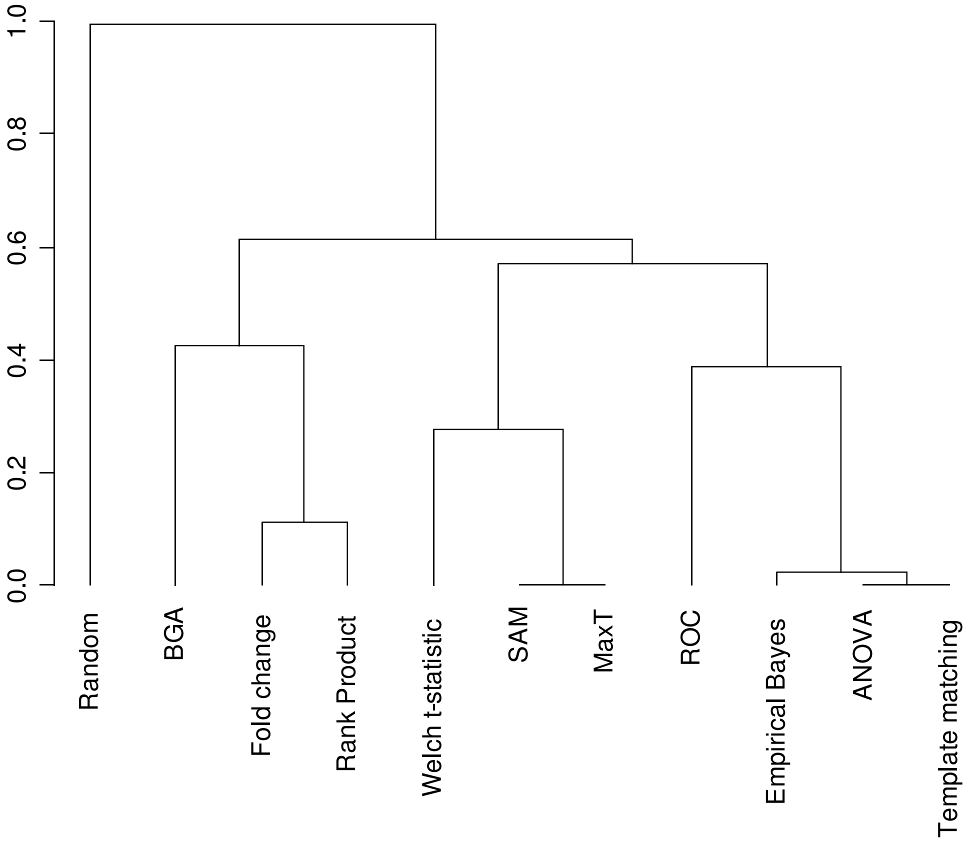

	BGA	SAM	ANOVA	Template matching	Welch t-statistic	Fold change	Empirical Bayes	MaxT	ROC	Rank Produc
BGA	/	51.8	54.4	54.4	44.9	72.8	55	51.8	51.3	73.1
SAM	51.8	/	59.1	59.1	83.9	58.9	59.3	100	78.6	59.6
ANOVA	54.4	59.1	/	100	44.2	57.3	98.9	59.1	76	59.7
Template matching	54.4	59.1	100	/	44.2	57.3	98.9	59.1	76	59.7
Welch t-statistic	44.9	83.9	44.2	44.2	/	51.5	44.3	83.9	65	50.5
Fold change	72.8	58.9	57.3	57.3	51.5	/	58.1	58.9	56.3	94.1
Empirical Bayes	55	59.3	98.9	98.9	44.3	58.1	/	59.3	76	60.4
MaxT	51.8	100	59.1	59.1	83.9	58.9	59.3	/	78.6	59.6
ROC	51.3	78.6	76	76	65	56.3	76	78.6	/	58.1
Rank Product	73.1	59.6	59.7	59.7	50.5	94.1	60.4	59.6	58.1	/
Random	1	1	0.7	0.7	0.9	0.9	0.7	1	0.9	0.9

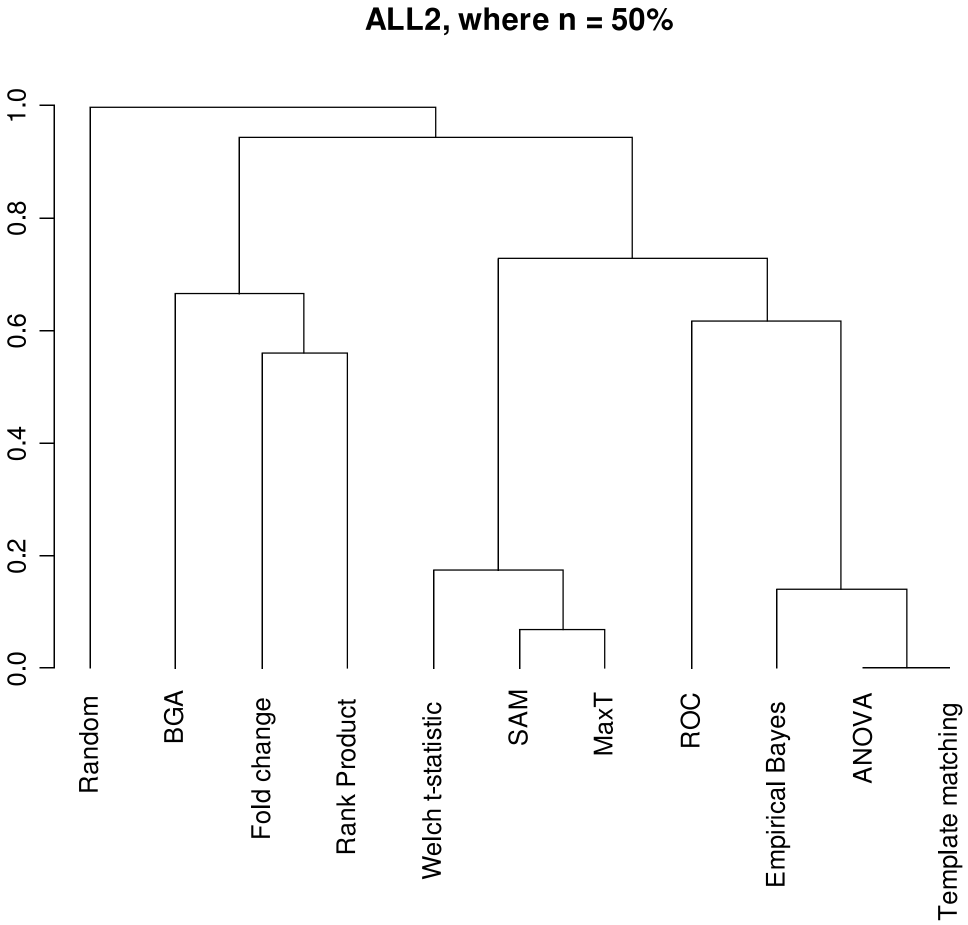

	BGA	SAM	ANOVA	Template matching	Welch t-statistic	Fold change	Empirical Bayes	MaxT	ROC	Rank Produc
BGA	/	17.6	12.1	12.1	17.1	58.5	14.3	16.3	9.5	41.1
SAM	17.6	/	40.6	40.6	89.1	15.9	40.5	96.4	53.8	7.1
ANOVA	12.1	40.6	/	100	35	11.1	92.4	41.3	55.8	5
Template matching	12.1	40.6	100	/	35	11.1	92.4	41.3	55.8	5
Welch t-statistic	17.1	89.1	35	35	/	14.2	34.9	91.9	50.6	6.5
Fold change	58.5	15.9	11.1	11.1	14.2	/	12.7	14.2	9.8	61.3
Empirical Bayes	14.3	40.5	92.4	92.4	34.9	12.7	/	41.1	55	5.5
MaxT	16.3	96.4	41.3	41.3	91.9	14.2	41.1	/	56.1	6.5
ROC	9.5	53.8	55.8	55.8	50.6	9.8	55	56.1	/	5
Rank Product	41.1	7.1	5	5	6.5	61.3	5.5	6.5	5	/
Random	0.7	0.3	0.9	0.9	0.2	0.3	0.9	0.3	0.6	1

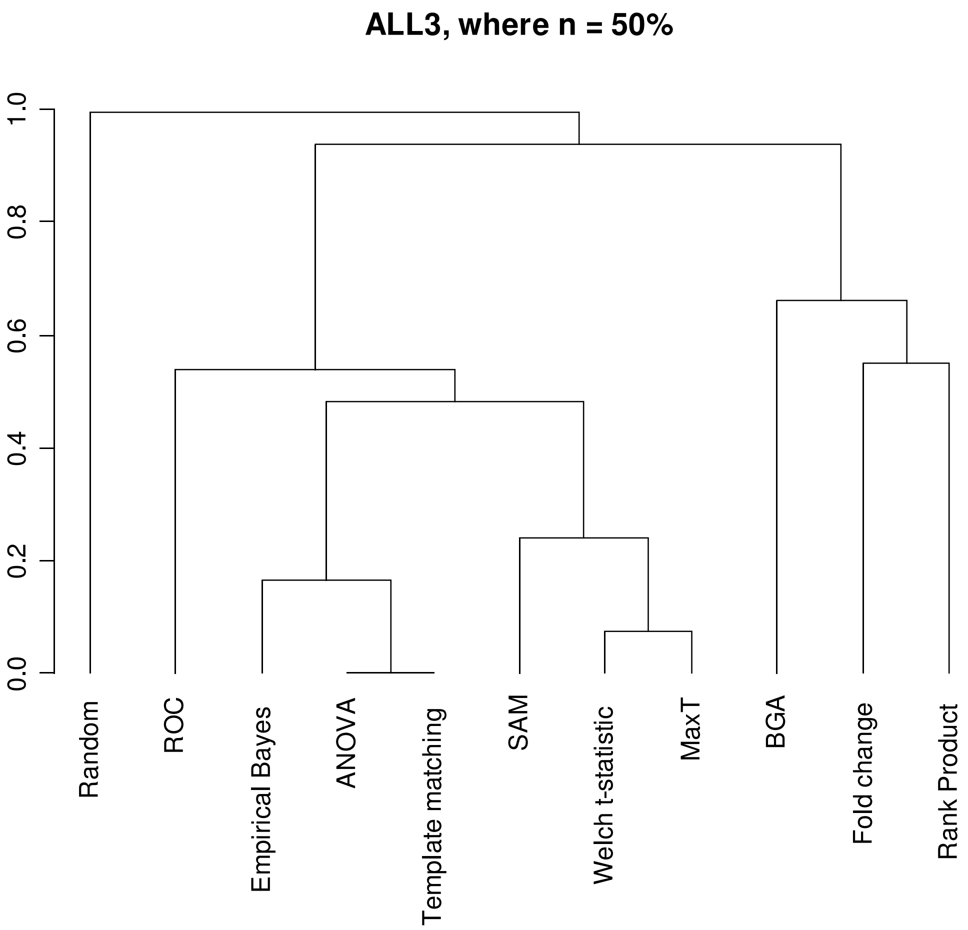

	BGA	SAM	ANOVA	Template matching	Welch t-statistic	Fold change	Empirical Bayes	MaxT	ROC	Rank Produc
BGA	/	21.2	11.7	11.7	16.9	59.5	13.8	16.4	11.1	40.3
SAM	21.2	/	66.1	66.1	85	18	67.4	87.6	58.5	8.2
ANOVA	11.7	66.1	/	100	67.6	11.9	90.9	71.4	64.9	5.7
Template matching	11.7	66.1	100	/	67.6	11.9	90.9	71.4	64.9	5.7
Welch t-statistic	16.9	85	67.6	67.6	/	13.7	66.9	96.1	62.6	6
Fold change	59.5	18	11.9	11.9	13.7	/	14.9	13.6	10.4	62.1
Empirical Bayes	13.8	67.4	90.9	90.9	66.9	14.9	/	70.5	64.2	7.3
MaxT	16.4	87.6	71.4	71.4	96.1	13.6	70.5	/	63.4	6
ROC	11.1	58.5	64.9	64.9	62.6	10.4	64.2	63.4	/	5.3
Rank Product	40.3	8.2	5.7	5.7	6	62.1	7.3	6	5.3	/
Random	0.6	0.8	0.9	0.9	0.7	0.8	0.9	0.7	0.7	0.8

ALL4, where n = 50%

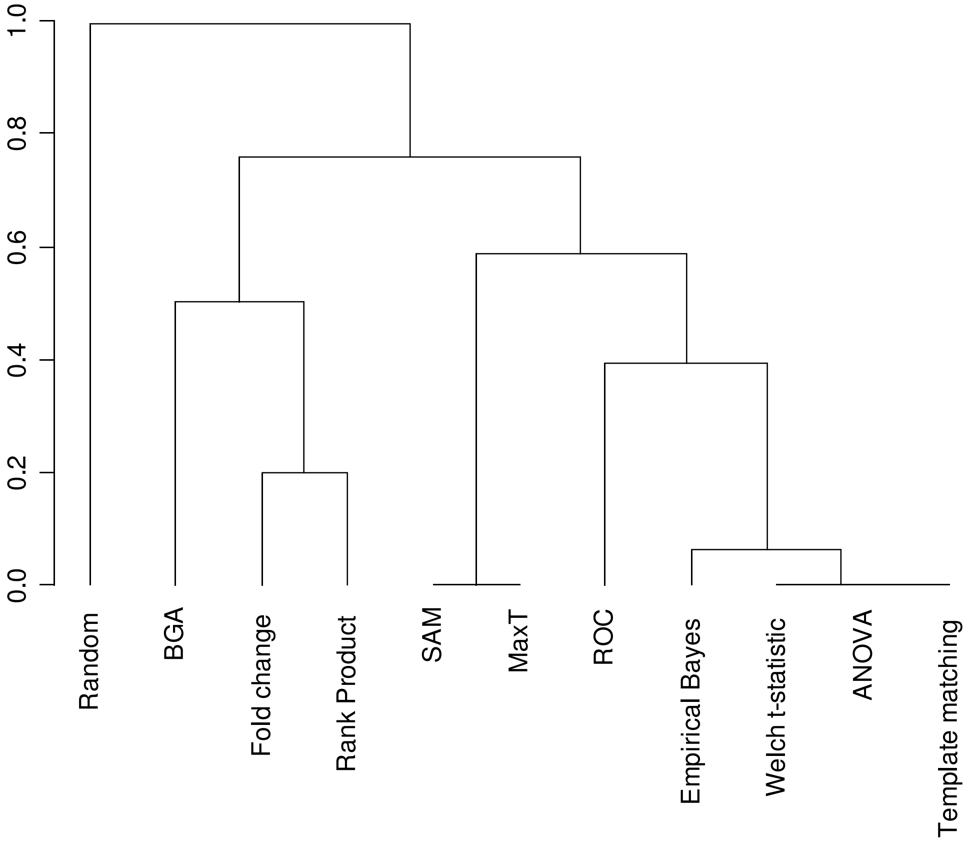

	BGA	SAM	ANOVA	Template matching	Welch t-statistic	Fold change	Empirical Bayes	MaxT	ROC	Rank Produc
BGA	/	37.5	39.3	39.3	39.3	68.1	40.3	37.5	38.1	65.1
SAM	37.5	/	56.4	56.4	56.4	45.1	57.1	100	65	41.7
ANOVA	39.3	56.4	/	100	100	37.9	96.8	56.4	75.4	35.5
Template matching	39.3	56.4	100	/	100	37.9	96.8	56.4	75.4	35.5
Welch t-statistic	39.3	56.4	100	100	/	37.9	96.8	56.4	75.4	35.5
Fold change	68.1	45.1	37.9	37.9	37.9	/	39.3	45.1	38.2	88.9
Empirical Bayes	40.3	57.1	96.8	96.8	96.8	39.3	/	57.1	75.9	36.7
MaxT	37.5	100	56.4	56.4	56.4	45.1	57.1	/	65	41.7
ROC	38.1	65	75.4	75.4	75.4	38.2	75.9	65	/	36.2
Rank Product	65.1	41.7	35.5	35.5	35.5	88.9	36.7	41.7	36.2	/
Random	0.8	0.7	1	1	1	0.8	1	0.7	0.9	0.8

Colon, where n = 50%

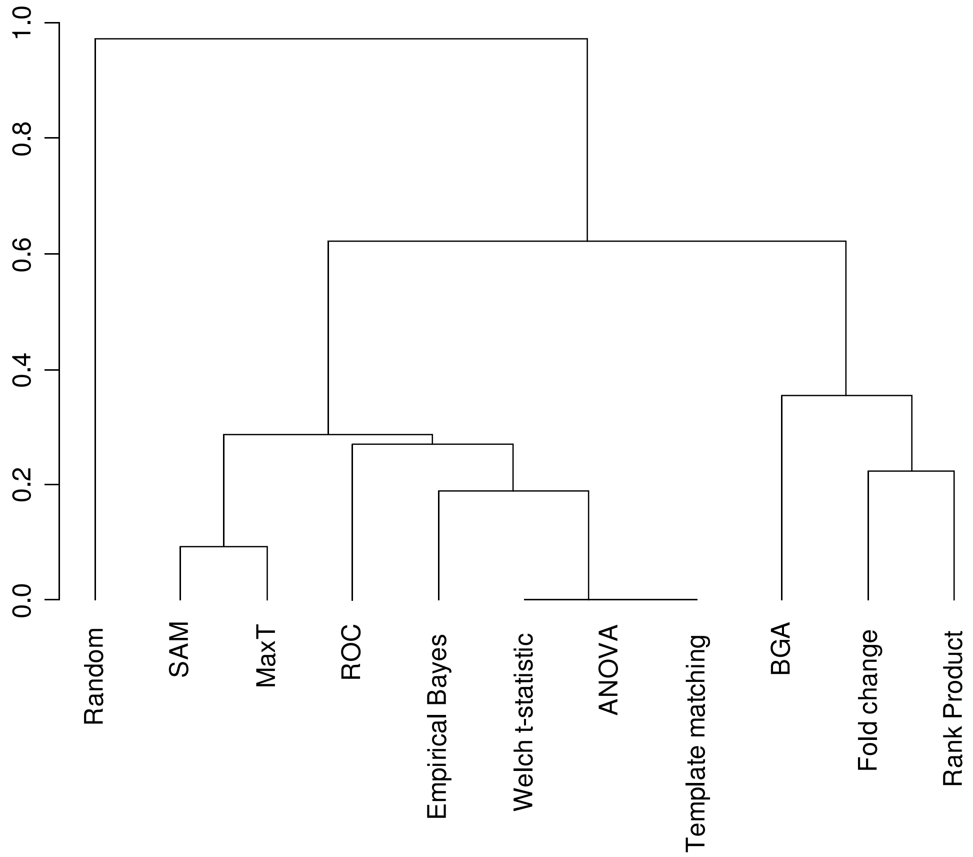

	BGA	SAM	ANOVA	Template matching	Welch t-statistic	Fold change	Empirical Bayes	MaxT	ROC	Rank Produc
BGA	/	56.4	54.6	54.6	54.6	80.2	63	52.7	53.1	76.7
SAM	56.4	/	82.9	82.9	82.9	60.3	83.2	95.2	82.8	50.3
ANOVA	54.6	82.9	/	100	100	57.9	89.6	84.3	85.2	47.9
Template matching	54.6	82.9	100	/	100	57.9	89.6	84.3	85.2	47.9
Welch t-statistic	54.6	82.9	100	100	/	57.9	89.6	84.3	85.2	47.9
Fold change	80.2	60.3	57.9	57.9	57.9	/	66.7	56.2	56.7	87.4
Empirical Bayes	63	83.2	89.6	89.6	89.6	66.7	/	80.9	81.5	56.2
MaxT	52.7	95.2	84.3	84.3	84.3	56.2	80.9	/	84.2	46.4
ROC	53.1	82.8	85.2	85.2	85.2	56.7	81.5	84.2	/	46.9
Rank Product	76.7	50.3	47.9	47.9	47.9	87.4	56.2	46.4	46.9	/
Random	4.8	5.2	5.3	5.3	5.3	4.8	5.6	5.3	5.4	4.3

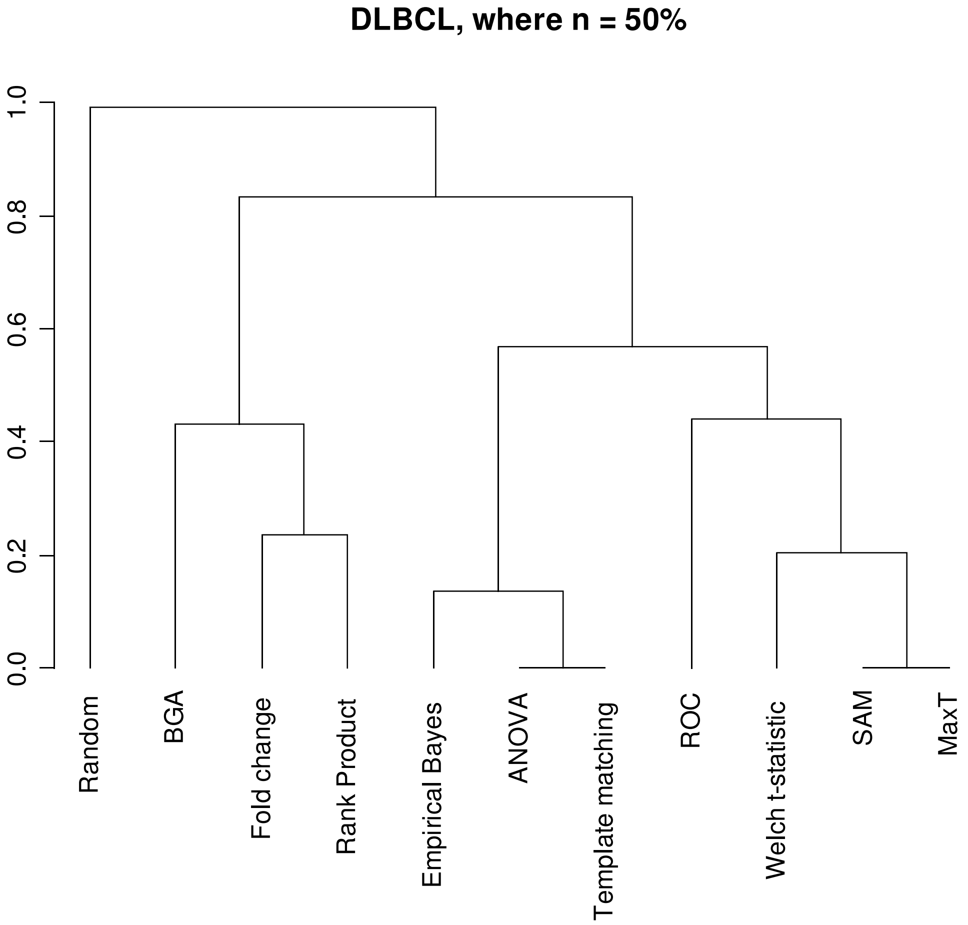

	BGA	SAM	ANOVA	Template matching	Welch t-statistic	Fold change	Empirical Bayes	MaxT	ROC	Rank Produc
BGA	/	26.7	26.5	26.5	26.5	73.8	29.7	26.7	24.9	71.2
SAM	26.7	/	58.9	58.9	88.6	30.4	59.6	100	74.6	28.1
ANOVA	26.5	58.9	/	100	48.2	30.3	92.7	58.9	71.9	29.2
Template matching	26.5	58.9	100	/	48.2	30.3	92.7	58.9	71.9	29.2
Welch t-statistic	26.5	88.6	48.2	48.2	/	30.1	49.5	88.6	65.8	27.3
Fold change	73.8	30.4	30.3	30.3	30.1	/	33.4	30.4	27.6	86.6
Empirical Bayes	29.7	59.6	92.7	92.7	49.5	33.4	/	59.6	71.5	32
MaxT	26.7	100	58.9	58.9	88.6	30.4	59.6	/	74.6	28.1
ROC	24.9	74.6	71.9	71.9	65.8	27.6	71.5	74.6	/	25.6
Rank Product	71.2	28.1	29.2	29.2	27.3	86.6	32	28.1	25.6	/
Random	1.1	1	1.8	1.8	1	1.3	1.6	1	1.2	1.5

Leukaemia, where n = 50%

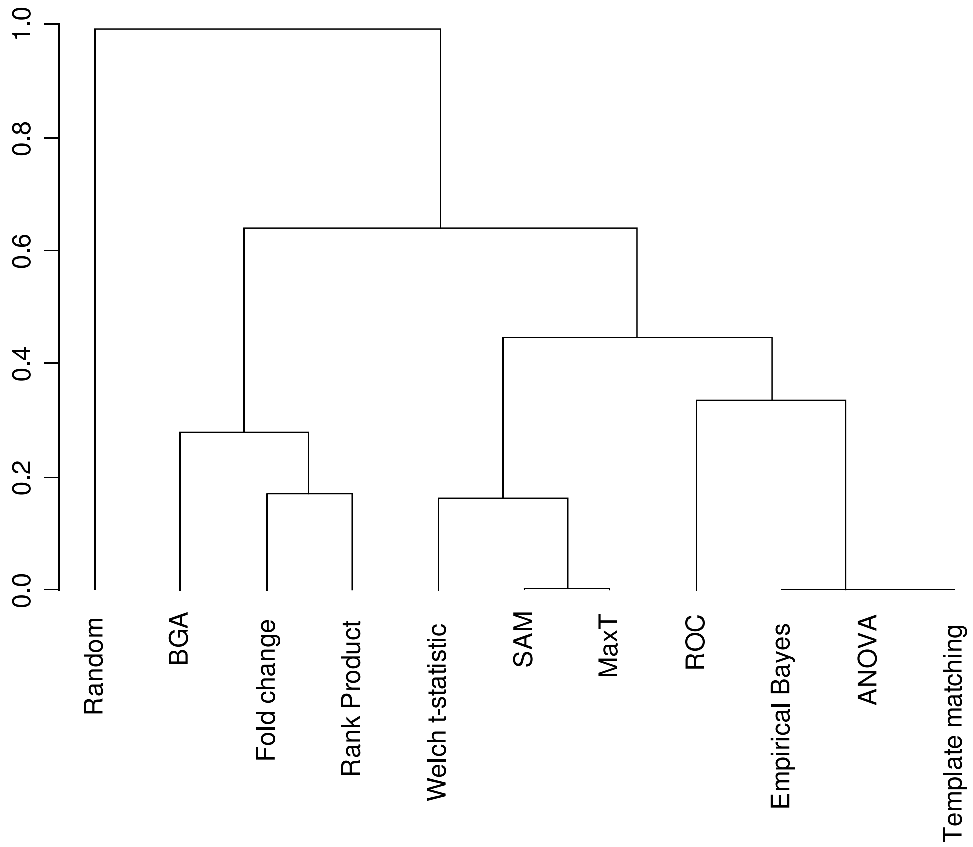

	BGA	SAM	ANOVA	Template matching	Welch t-statistic	Fold change	Empirical Bayes	MaxT	ROC	Rank Produc
BGA	/	49.3	54.6	54.6	44.2	85.1	54.6	49.3	46.4	82.5
SAM	49.3	/	72	72	91.2	53.5	72	99.8	79.3	52.1
ANOVA	54.6	72	/	100	63.4	59	100	72	79.9	56.2
Template matching	54.6	72	100	/	63.4	59	100	72	79.9	56.2
Welch t-statistic	44.2	91.2	63.4	63.4	/	48.6	63.4	91.2	72.8	47.2
Fold change	85.1	53.5	59	59	48.6	/	59	53.5	52.8	90.7
Empirical Bayes	54.6	72	100	100	63.4	59	/	72	79.9	56.2
MaxT	49.3	99.8	72	72	91.2	53.5	72	/	79.2	52.1
ROC	46.4	79.3	79.9	79.9	72.8	52.8	79.9	79.2	/	50.7
Rank Product	82.5	52.1	56.2	56.2	47.2	90.7	56.2	52.1	50.7	/
Random	1.3	1.4	1.2	1.2	1.3	1.4	1.2	1.4	1	1.4

Myeloma, where n = 50%

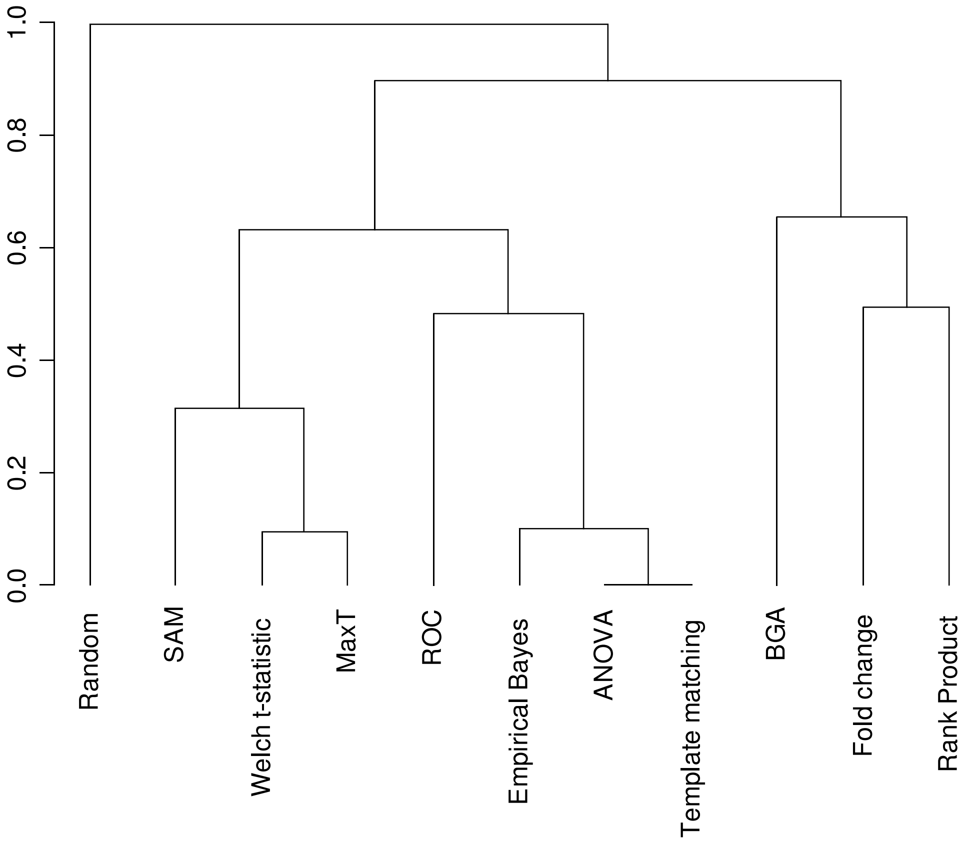

	BGA	SAM	ANOVA	Template matching	Welch t-statistic	Fold change	Empirical Bayes	MaxT	ROC	Rank Produc
BGA	/	23	17	17	15.7	56.8	19	16.3	14.4	45.6
SAM	23	/	52.4	52.4	80.8	31.5	53.5	82	62.1	16.8
ANOVA	17	52.4	/	100	48.2	26.3	94.7	52.8	68.5	12.2
Template matching	17	52.4	100	/	48.2	26.3	94.7	52.8	68.5	12.2
Welch t-statistic	15.7	80.8	48.2	48.2	/	20.3	47.6	95	59.8	10.7
Fold change	56.8	31.5	26.3	26.3	20.3	/	28.5	21.6	22.7	67.1
Empirical Bayes	19	53.5	94.7	94.7	47.6	28.5	/	52.1	67.4	13.8
MaxT	16.3	82	52.8	52.8	95	21.6	52.1	/	63.6	11.3
ROC	14.4	62.1	68.5	68.5	59.8	22.7	67.4	63.6	/	10.7
Rank Product	45.6	16.8	12.2	12.2	10.7	67.1	13.8	11.3	10.7	/
Random	0.5	0.4	0.8	0.8	0.4	0.9	0.7	0.4	0.4	0.9

Prostate, where n = 50%

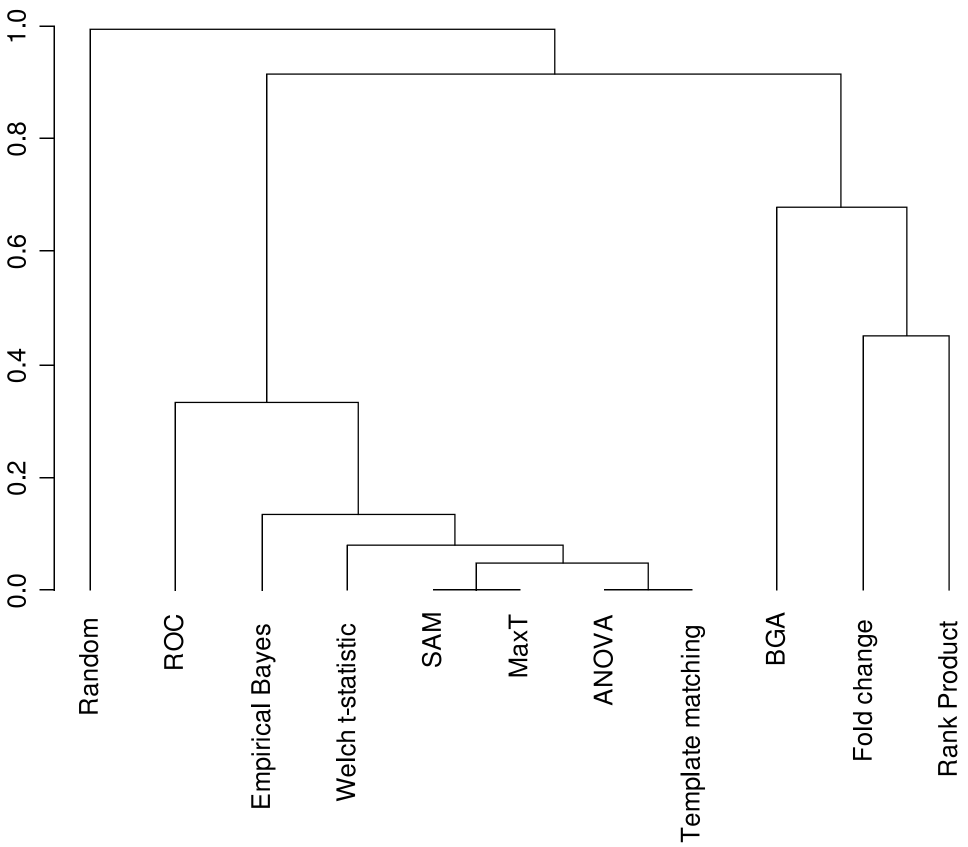

	BGA	SAM	ANOVA	Template matching	Welch t-statistic	Fold change	Empirical Bayes	MaxT	ROC	Rank Produc
BGA	/	18.9	19.2	19.2	17.9	47.7	20.9	18.9	19.9	49.7
SAM	18.9	/	97.5	97.5	95.8	12.7	93.5	100	80.4	15.6
ANOVA	19.2	97.5	/	100	95.8	12.8	93.2	97.5	80.1	15.8
Template matching	19.2	97.5	100	/	95.8	12.8	93.2	97.5	80.1	15.8
Welch t-statistic	17.9	95.8	95.8	95.8	/	12.1	90.5	95.8	79.2	14.7
Fold change	47.7	12.7	12.8	12.8	12.1	/	14	12.7	12	71
Empirical Bayes	20.9	93.5	93.2	93.2	90.5	14	/	93.5	79.3	17.3
MaxT	18.9	100	97.5	97.5	95.8	12.7	93.5	/	80.4	15.6
ROC	19.9	80.4	80.1	80.1	79.2	12	79.3	80.4	/	13.4
Rank Product	49.7	15.6	15.8	15.8	14.7	71	17.3	15.6	13.4	/
Random	1.1	1.2	1.1	1.1	1.2	0.9	1.1	1.2	1	1
