# Supplementary material for: Comparison and evaluation of methods for generating differentially expressed gene lists from microarray data
Source: BMC Bioinformatics. 2006 Jul 26;7:359. doi: 10.1186/1471-2105-7-359 (PMC1544358; doi:10.1186/1471-2105-7-359)
Supplement: Additional File 4 — Overlap in gene lists produced by different feature selection methods when applied to each dataset. Each feature selection method was applied to each of the full datasets. The overlap of genes ranked in the top 100 by each method was compared using a binary distance metric. Dendrograms show the results of average linkage hierarchical cluster analysis of these scores for each dataset. Percentage matricies below each of the dendrograms show the percentage similarity between each of the feature selection methods. [file 1471-2105-7-359-S4.pdf]

Full ALL1 dataset

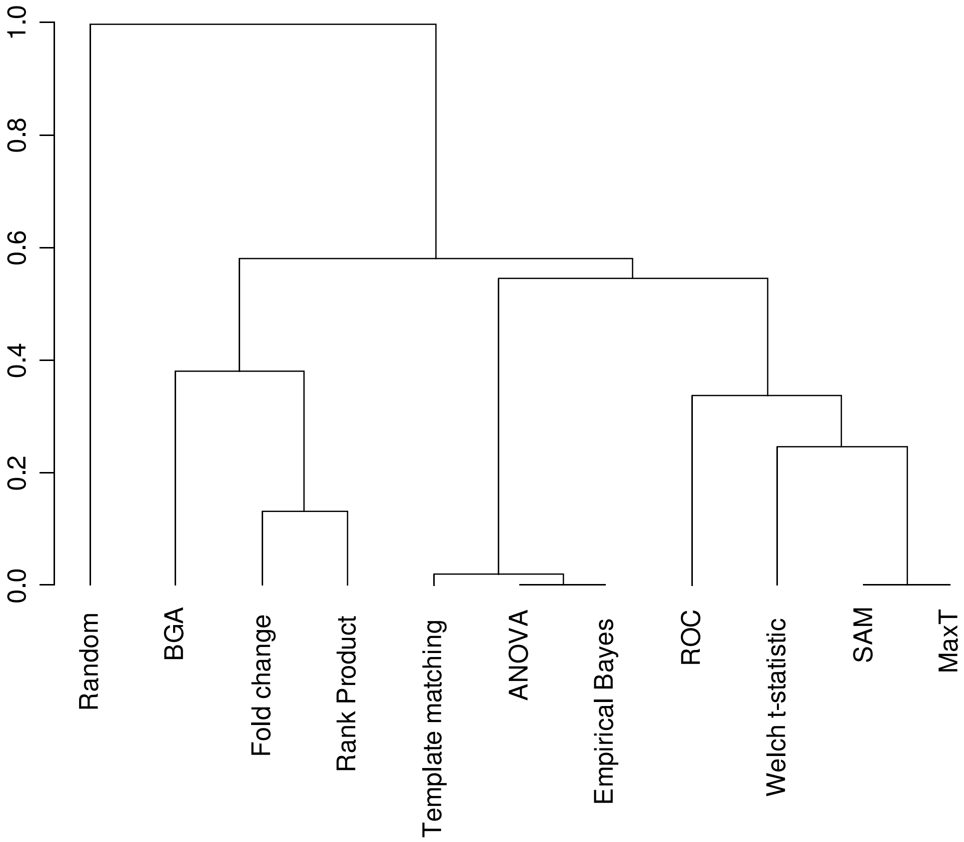

	BGA	SAM	ANOVA	Template matching	Welch t-statistic	Fold change	Empirical Bayes	MaxT	ROC	Rank Product
BGA	/	56	57	56	49	76	57	56	58	77
SAM	56	/	62	61	86	60	62	100	84	62
ANOVA	57	62	/	99	48	60	100	62	76	64
Template matching	56	61	99	/	47	59	99	61	75	63
Welch t-statistic	49	86	48	47	/	54	48	86	70	54
Fold change	76	60	60	59	54	/	60	60	62	93
Empirical Bayes	57	62	100	99	48	60	/	62	76	64
MaxT	56	100	62	61	86	60	62	/	84	62
ROC	58	84	76	75	70	62	76	84	/	64
Rank Product	77	62	64	63	54	93	64	62	64	/
Random	0	0	1	1	0	0	1	0	0	0

Full ALL2 dataset

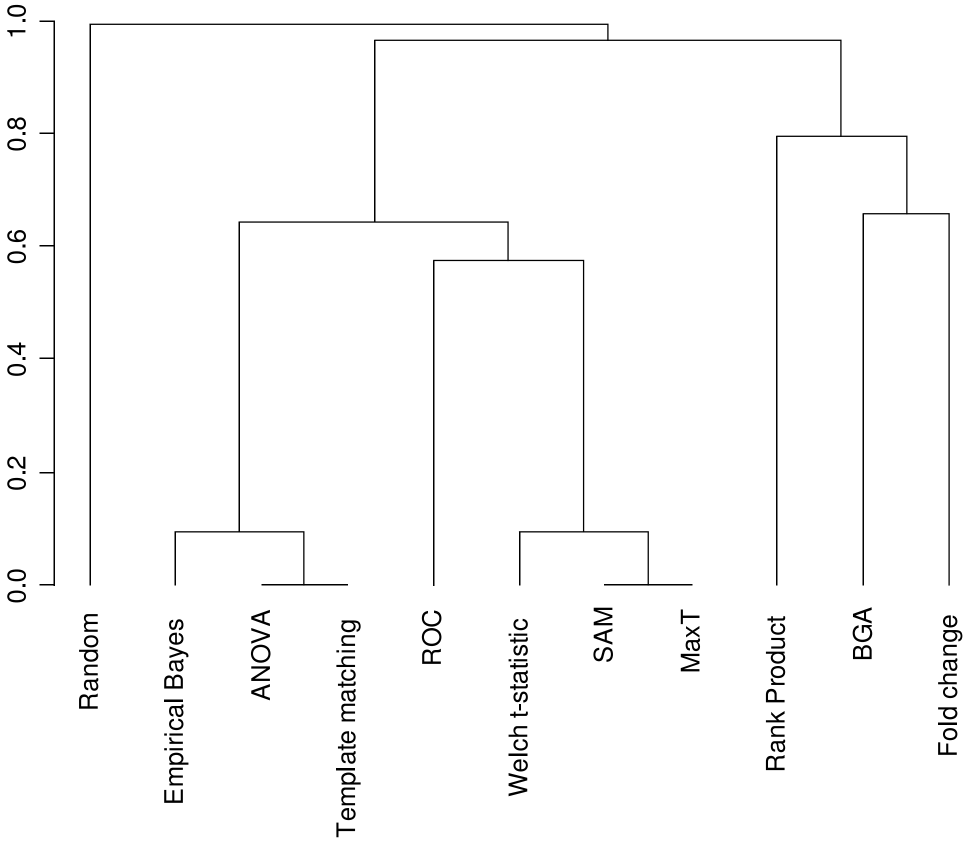

	BGA	SAM	ANOVA	Template matching	Welch t-statistic	Fold change	Empirical Bayes	MaxT	ROC	Rank Product
BGA	/	10	8	8	11	51	10	10	7	28
SAM	10	/	53	53	95	12	54	100	61	1
ANOVA	8	53	/	100	49	8	95	53	54	0
Template matching	8	53	100	/	49	8	95	53	54	0
Welch t-statistic	11	95	49	49	/	13	51	95	57	1
Fold change	51	12	8	8	13	/	10	12	8	40
Empirical Bayes	10	54	95	95	51	10	/	54	55	0
MaxT	10	100	53	53	95	12	54	/	61	1
ROC	7	61	54	54	57	8	55	61	/	0
Rank Product	28	1	0	0	1	40	0	1	0	/
Random	1	3	0	0	3	1	0	3	0	1

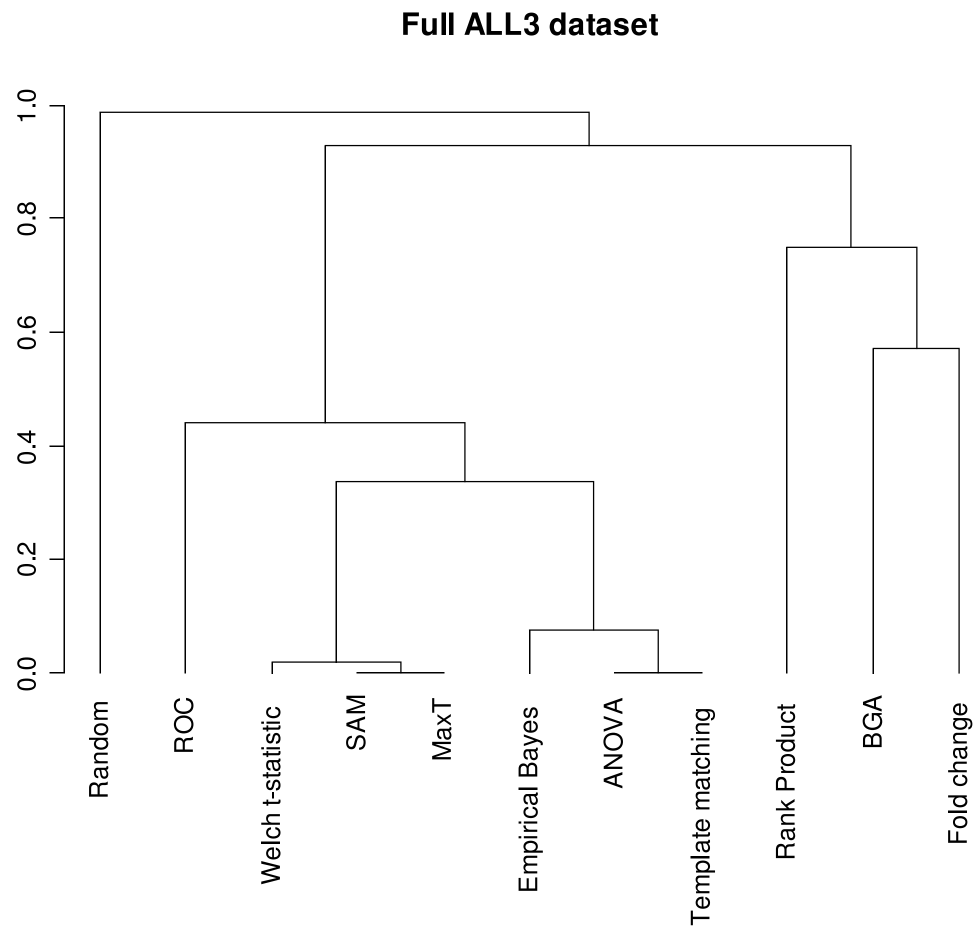

	BGA	SAM	ANOVA	Template matching	Welch t-statistic	Fold change	Empirical Bayes	MaxT	ROC	Rank Product
BGA	/	18	17	17	17	60	18	18	10	32
SAM	18	/	80	80	99	18	80	100	74	5
ANOVA	17	80	/	100	79	16	96	80	69	7
Template matching	17	80	100	/	79	16	96	80	69	7
Welch t-statistic	17	99	79	79	/	17	79	99	74	5
Fold change	60	18	16	16	17	/	20	18	14	47
Empirical Bayes	18	80	96	96	79	20	/	80	70	9
MaxT	18	100	80	80	99	18	80	/	74	5
ROC	10	74	69	69	74	14	70	74	/	6
Rank Product	32	5	7	7	5	47	9	5	6	/
Random	0	3	3	3	3	0	3	3	3	1

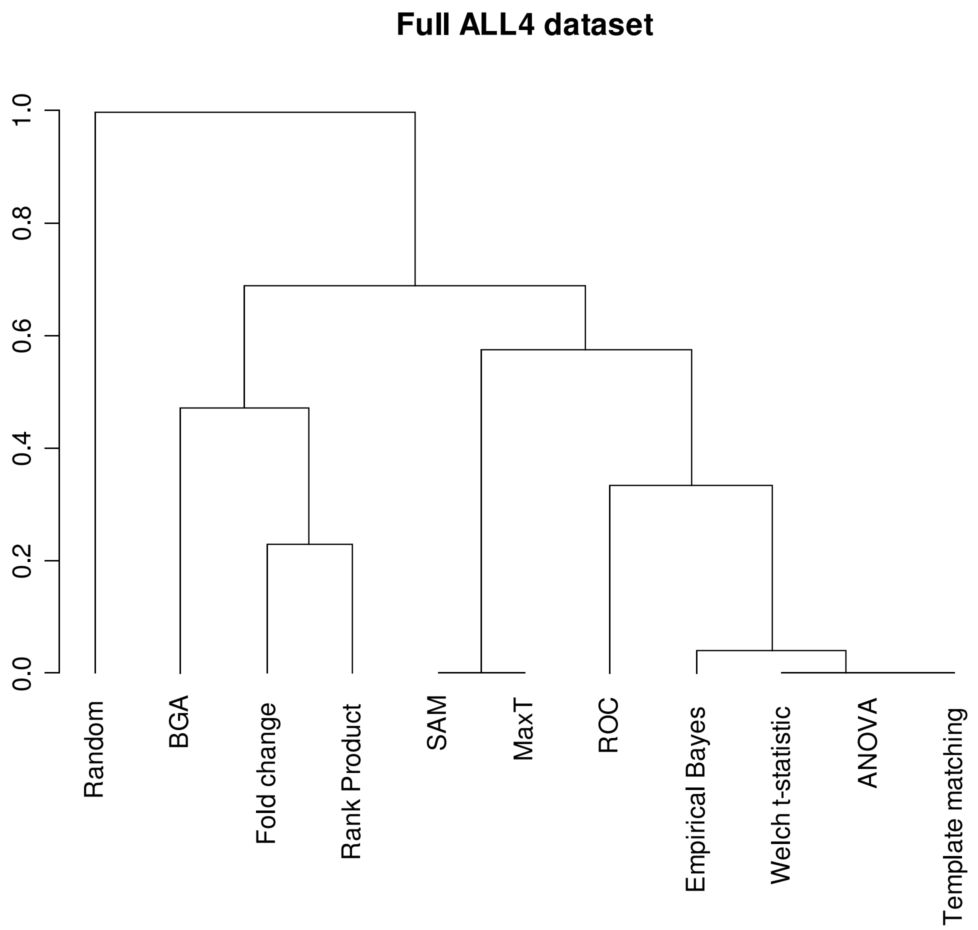

	BGA	SAM	ANOVA	Template matching	Welch t-statistic	Fold change	Empirical Bayes	MaxT	ROC	Rank Product
BGA	/	47	48	48	48	72	49	47	47	66
SAM	47	/	60	60	60	56	60	100	59	51
ANOVA	48	60	/	100	100	47	98	60	80	43
Template matching	48	60	100	/	100	47	98	60	80	43
Welch t-statistic	48	60	100	100	/	47	98	60	80	43
Fold change	72	56	47	47	47	/	47	56	45	87
Empirical Bayes	49	60	98	98	98	47	/	60	80	43
MaxT	47	100	60	60	60	56	60	/	59	51
ROC	47	59	80	80	80	45	80	59	/	42
Rank Product	66	51	43	43	43	87	43	51	42	/
Random	0	1	0	0	0	1	0	1	0	1

Full Colon dataset

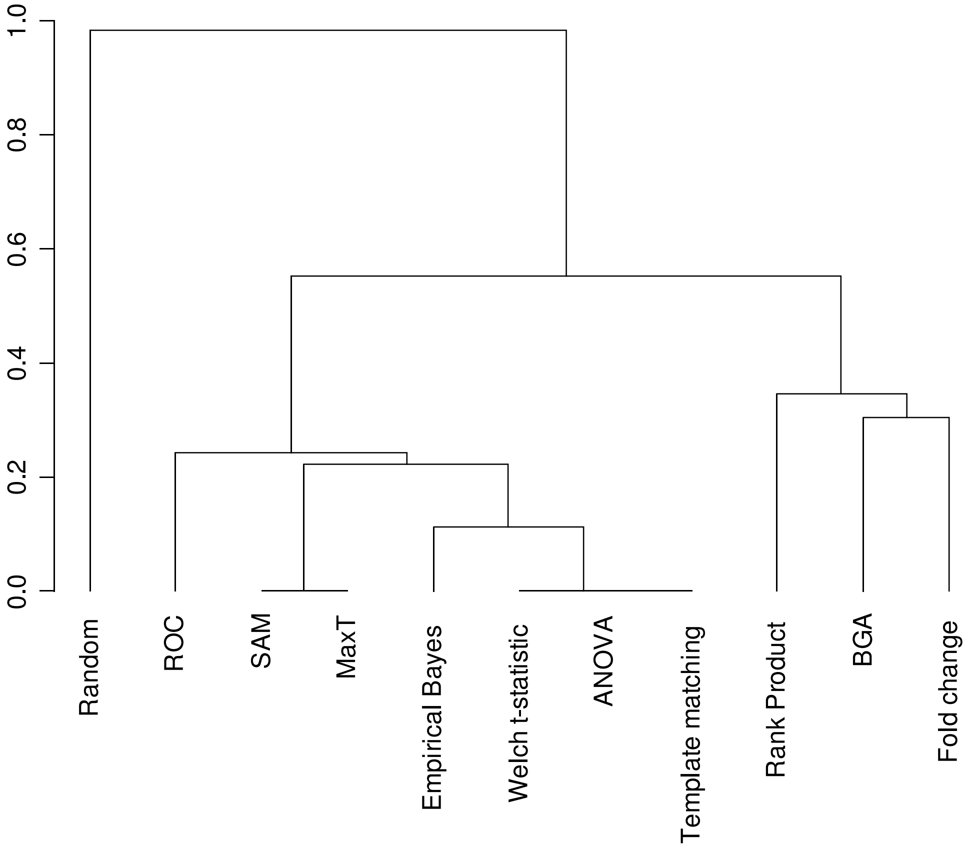

	BGA	SAM	ANOVA	Template matching	Welch t-statistic	Fold change	Empirical Bayes	MaxT	ROC	Rank Product
BGA	/	61	61	61	61	82	66	61	58	76
SAM	61	/	88	88	88	68	86	100	86	53
ANOVA	61	88	/	100	100	69	94	88	87	55
Template matching	61	88	100	/	100	69	94	88	87	55
Welch t-statistic	61	88	100	100	/	69	94	88	87	55
Fold change	82	68	69	69	69	/	73	68	67	82
Empirical Bayes	66	86	94	94	94	73	/	86	84	58
MaxT	61	100	88	88	88	68	86	/	86	53
ROC	58	86	87	87	87	67	84	86	/	53
Rank Product	76	53	55	55	55	82	58	53	53	/
Random	2	3	4	4	4	2	4	3	4	3

Full DLBCL dataset

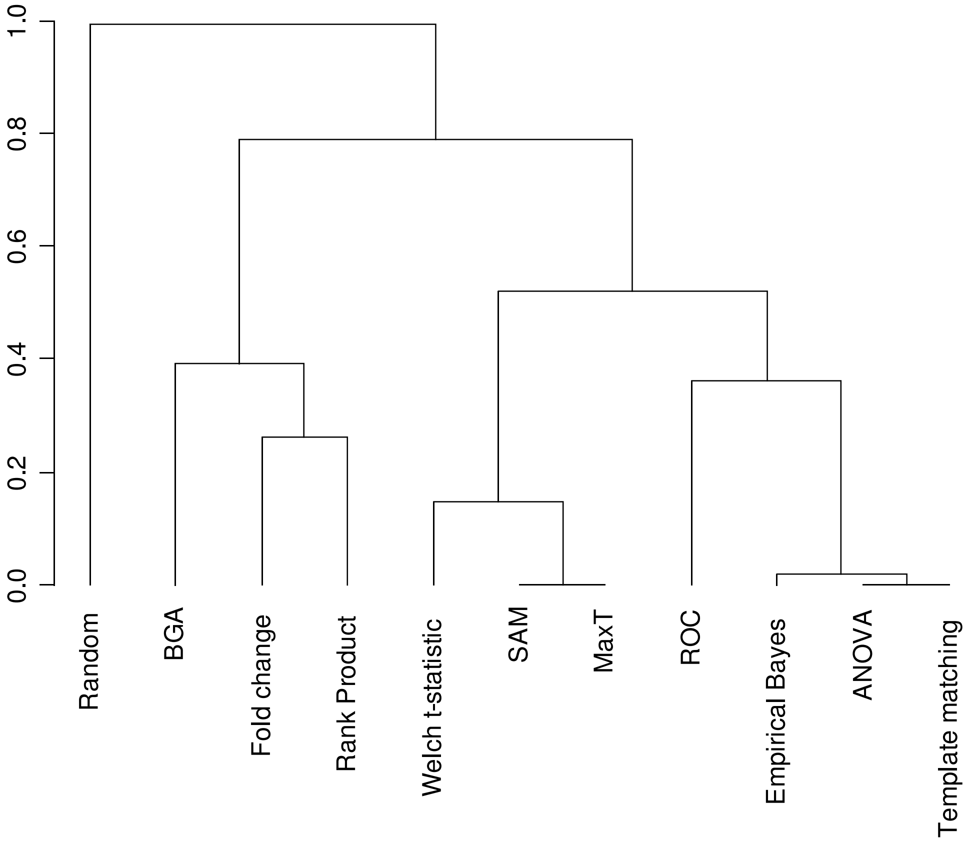

	BGA	SAM	ANOVA	Template matching	Welch t-statistic	Fold change	Empirical Bayes	MaxT	ROC	Rank Product
BGA	/	30	34	34	29	78	34	30	29	73
SAM	30	/	63	63	92	37	63	100	78	31
ANOVA	34	63	/	100	56	42	99	63	78	36
Template matching	34	63	100	/	56	42	99	63	78	36
Welch t-statistic	29	92	56	56	/	37	56	92	71	30
Fold change	78	37	42	42	37	/	42	37	38	85
Empirical Bayes	34	63	99	99	56	42	/	63	78	36
MaxT	30	100	63	63	92	37	63	/	78	31
ROC	29	78	78	78	71	38	78	78	/	33
Rank Product	73	31	36	36	30	85	36	31	33	/
Random	1	0	2	2	0	2	2	0	1	2

Full Leukaemia dataset

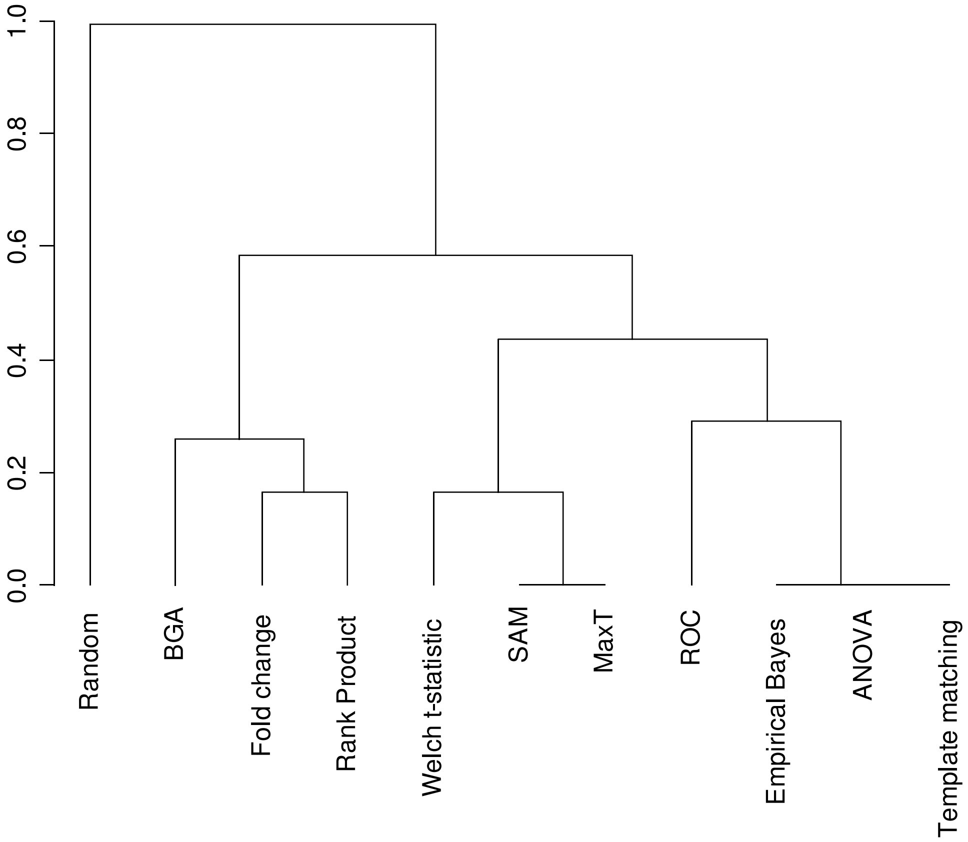

	BGA	SAM	ANOVA	Template matching	Welch t-statistic	Fold change	Empirical Bayes	MaxT	ROC	Rank Product
BGA	/	55	60	60	52	87	60	55	51	83
SAM	55	/	73	73	91	60	73	100	80	58
ANOVA	60	73	/	100	64	64	100	73	83	61
Template matching	60	73	100	/	64	64	100	73	83	61
Welch t-statistic	52	91	64	64	/	57	64	91	73	56
Fold change	87	60	64	64	57	/	64	60	58	91
Empirical Bayes	60	73	100	100	64	64	/	73	83	61
MaxT	55	100	73	73	91	60	73	/	80	58
ROC	51	80	83	83	73	58	83	80	/	56
Rank Product	83	58	61	61	56	91	61	58	56	/
Random	1	1	1	1	1	1	1	1	1	1

Full Myeloma dataset

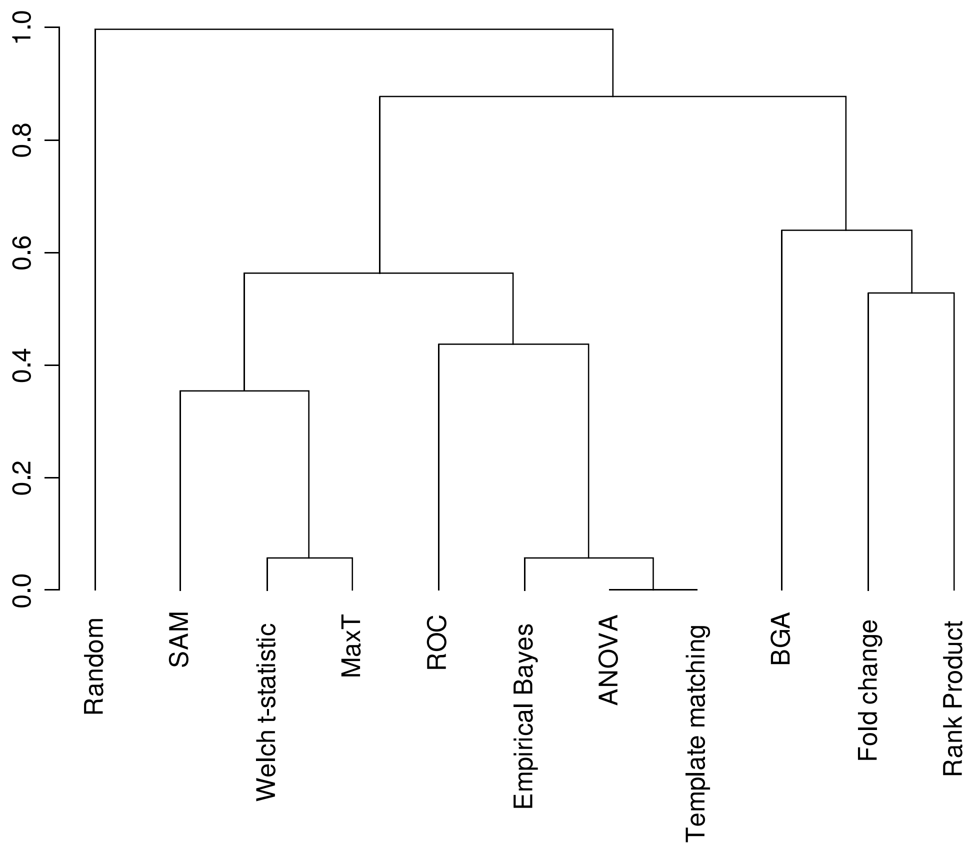

	BGA	SAM	ANOVA	Template matching	Welch t-statistic	Fold change	Empirical Bayes	MaxT	ROC	Rank Product
BGA	/	25	21	21	15	55	22	15	14	51
SAM	25	/	61	61	78	39	64	79	66	22
ANOVA	21	61	/	100	57	32	97	60	72	17
Template matching	21	61	100	/	57	32	97	60	72	17
Welch t-statistic	15	78	57	57	/	24	56	97	63	12
Fold change	55	39	32	32	24	/	33	24	25	64
Empirical Bayes	22	64	97	97	56	33	/	59	72	18
MaxT	15	79	60	60	97	24	59	/	65	12
ROC	14	66	72	72	63	25	72	65	/	11
Rank Product	51	22	17	17	12	64	18	12	11	/
Random	0	1	0	0	1	1	0	1	0	1

Full Prostate dataset

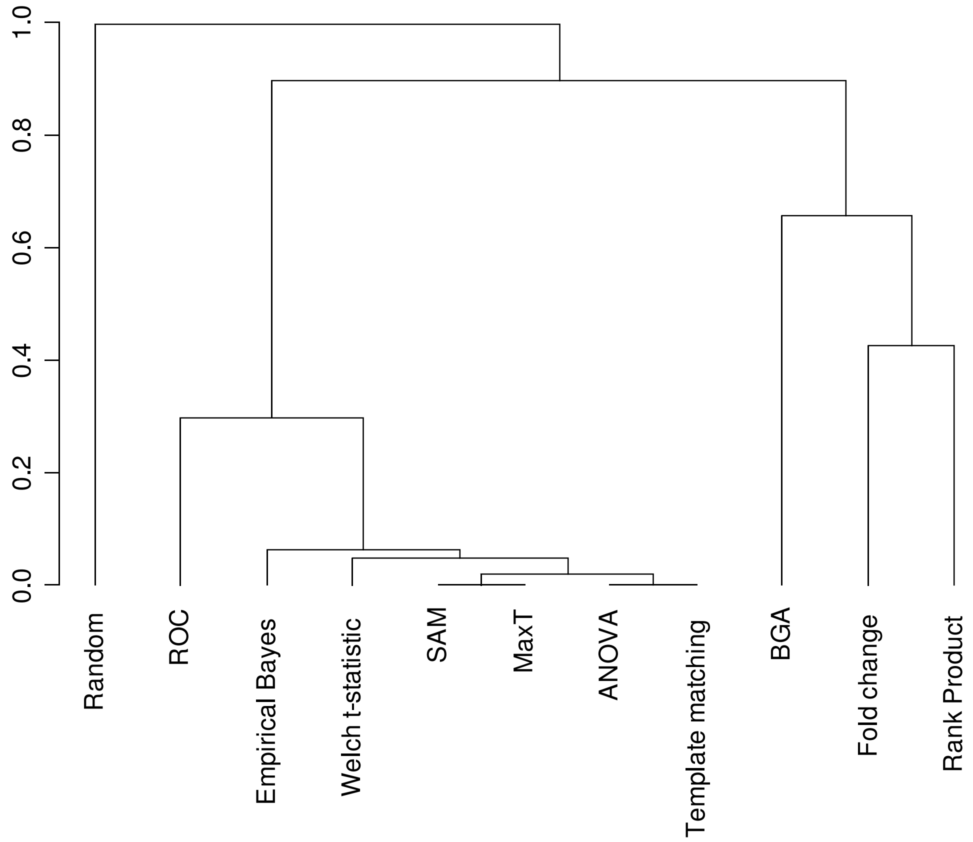

	BGA	SAM	ANOVA	Template matching	Welch t-statistic	Fold change	Empirical Bayes	MaxT	ROC	Rank Product
BGA	/	22	21	21	21	49	22	22	20	53
SAM	22	/	99	99	98	17	97	100	83	19
ANOVA	21	99	/	100	97	17	97	99	83	19
Template matching	21	99	100	/	97	17	97	99	83	19
Welch t-statistic	21	98	97	97	/	16	96	98	81	18
Fold change	49	17	17	17	16	/	17	17	13	73
Empirical Bayes	22	97	97	97	96	17	/	97	82	19
MaxT	22	100	99	99	98	17	97	/	83	19
ROC	20	83	83	83	81	13	82	83	/	15
Rank Product	53	19	19	19	18	73	19	19	15	/
Random	2	0	0	0	0	0	0	0	1	0
