# Supplementary material for: Comparison and evaluation of methods for generating differentially expressed gene lists from microarray data
Source: BMC Bioinformatics. 2006 Jul 26;7:359. doi: 10.1186/1471-2105-7-359 (PMC1544358; doi:10.1186/1471-2105-7-359)
Supplement: Additional File 9 — The RCI scores for each of the individual datasets and individual classification methods where the top 40 genes are used and n = 10 samples per class. RCI values showing the success of the top 40 genes, selected by the feature selection methods, to form classifiers which can predict the class of blind test data for each of the 9 datasets. These figures show the results for each of the classification methods when a reduced training set of 20 (10 from each class) is used. [file 1471-2105-7-359-S9.pdf]

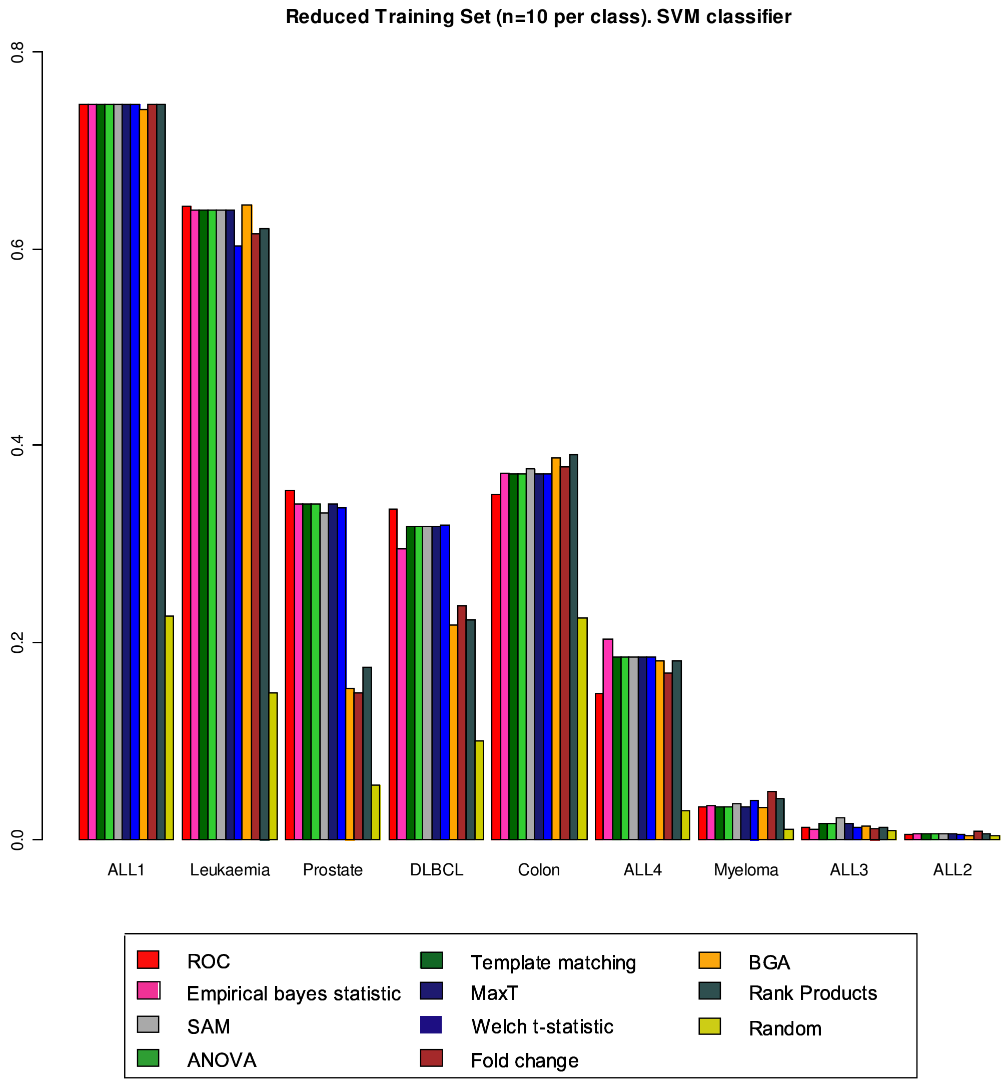
Reduced Training Set (n=10 per class). SVM classifier
0.0
0.2
0.4
0.6
0.8
ALL1
Leukaemia
Prostate
DLBCL
Colon
ALL4
Myeloma
ALL3
ALL2
ROC
Empirical bayes statistic
SAM
ANOVA
Template matching
MaxT
Welch t-statistic
Fold change
BGA
Rank Products
Random

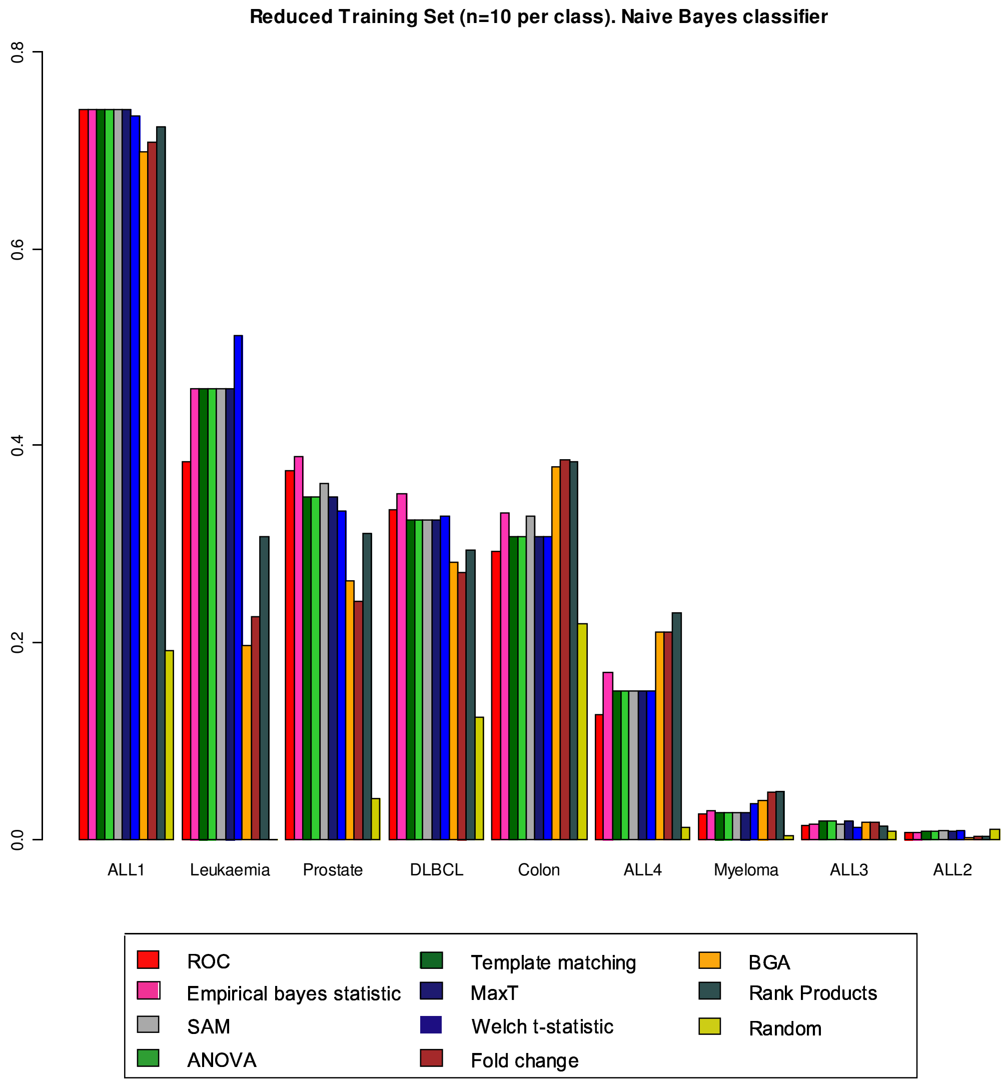

Reduced Training Set (n=10 per class). Naive Bayes classifier
0.0
0.2
0.4
0.6
0.8
ALL1
Leukaemia
Prostate
DLBCL
Colon
ALL4
Myeloma
ALL3
ALL2
ROC
Empirical bayes statistic
SAM
ANOVA
Template matching
MaxT
Welch t-statistic
Fold change
BGA
Rank Products
Random

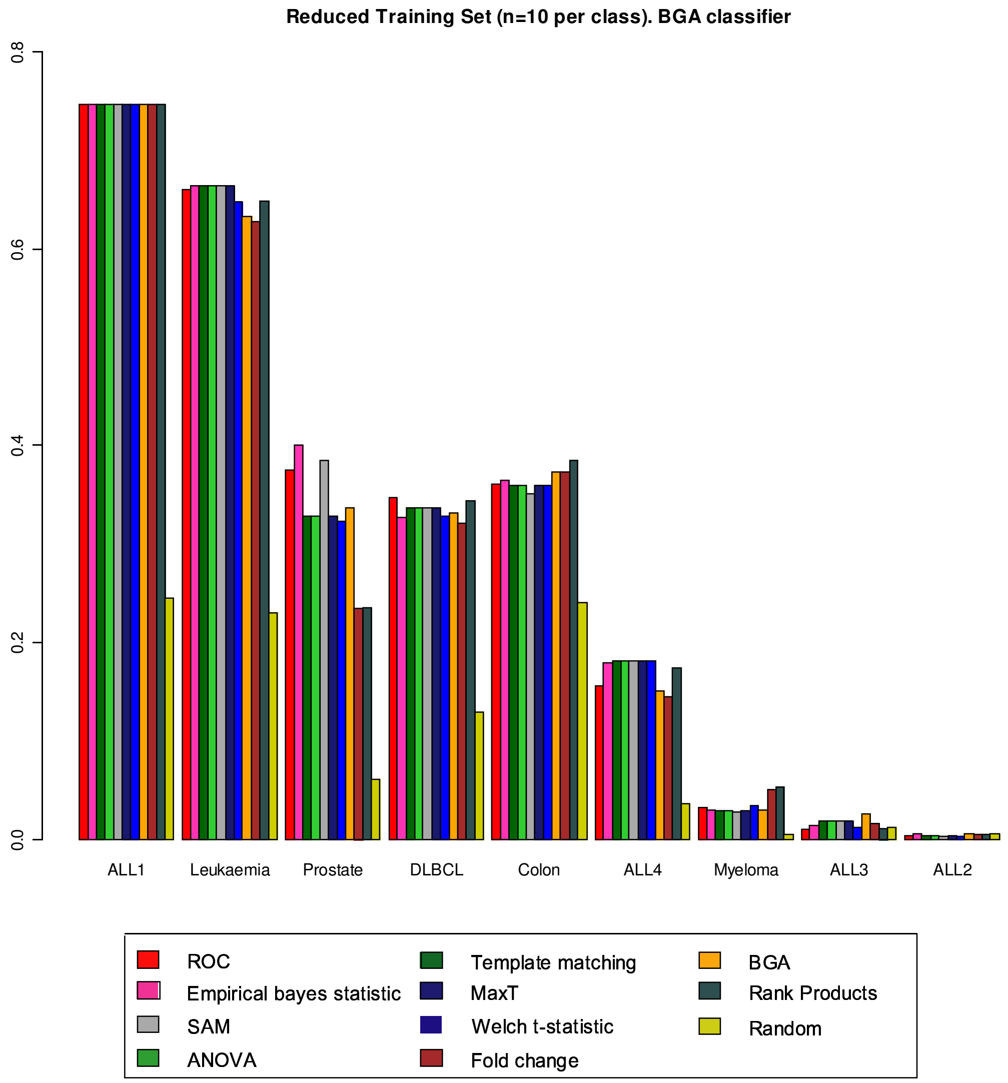
Reduced Training Set (n=10 per class). BGA classifier
0.0
0.2
0.4
0.6
0.8
ALL1
Leukaemia
Prostate
DLBCL
Colon
ALL4
Myeloma
ALL3
ALL2
ROC
Empirical bayes statistic
SAM
ANOVA
Template matching
MaxT
Welch t-statistic
Fold change
BGA
Rank Products
Random

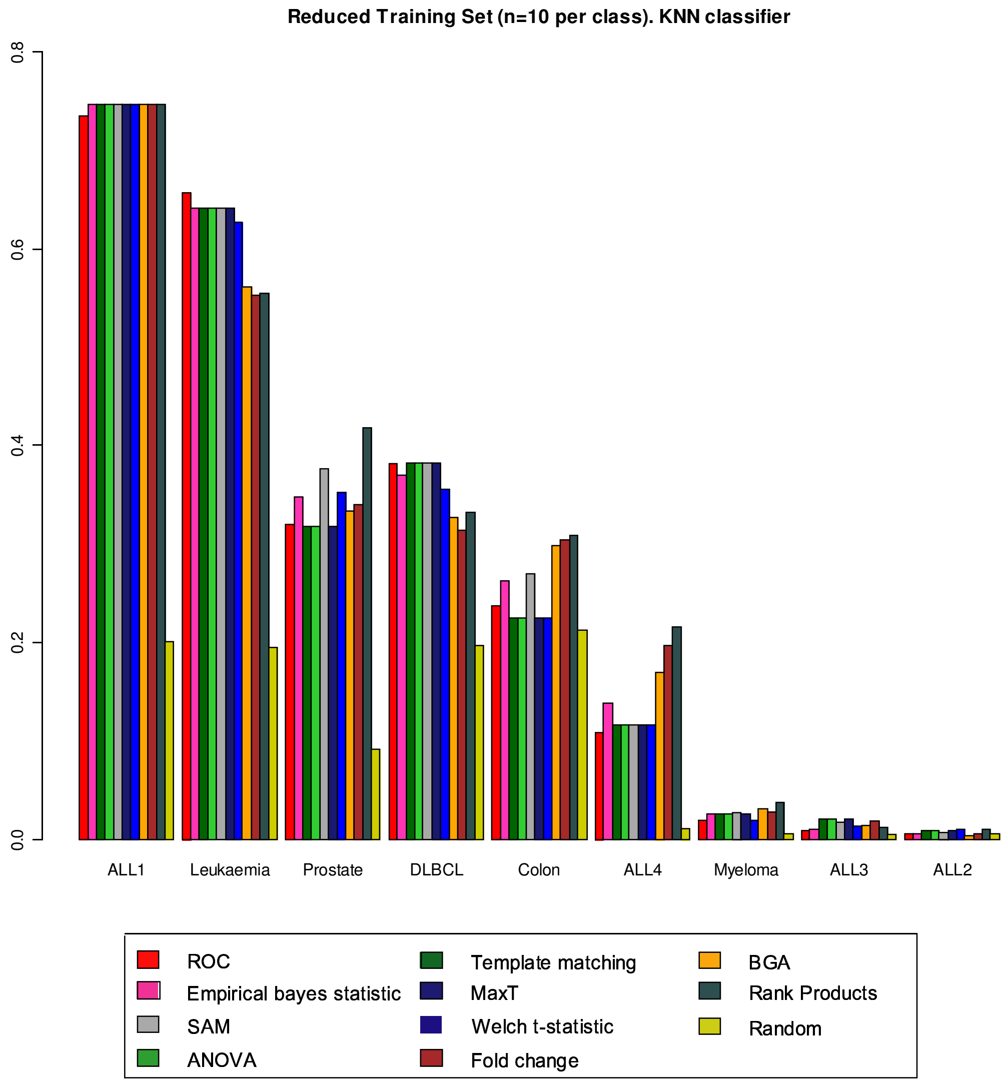
Reduced Training Set (n=10 per class). KNN classifier
0.0
0.2
0.4
0.6
0.8
ALL1
Leukaemia
Prostate
DLBCL
Colon
ALL4
Myeloma
ALL3
ALL2
ROC
Empirical bayes statistic
SAM
ANOVA
Template matching
MaxT
Welch t-statistic
Fold change
BGA
Rank Products
Random
