# Supplementary figures and images for: Comparison and evaluation of methods for generating differentially expressed gene lists from microarray data
Source: BMC Bioinformatics. 2006 Jul 26;7:359. doi: 10.1186/1471-2105-7-359 (PMC1544358; doi:10.1186/1471-2105-7-359)

[illegible]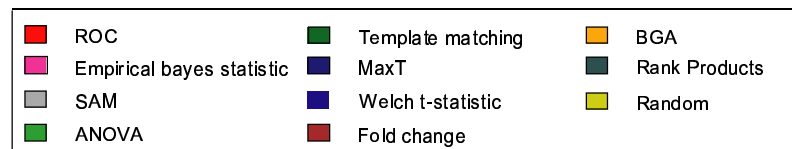

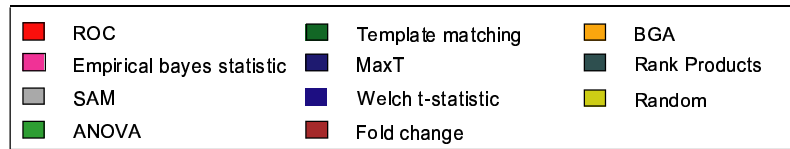

### Split Sample. Training and Test. BGA classifier

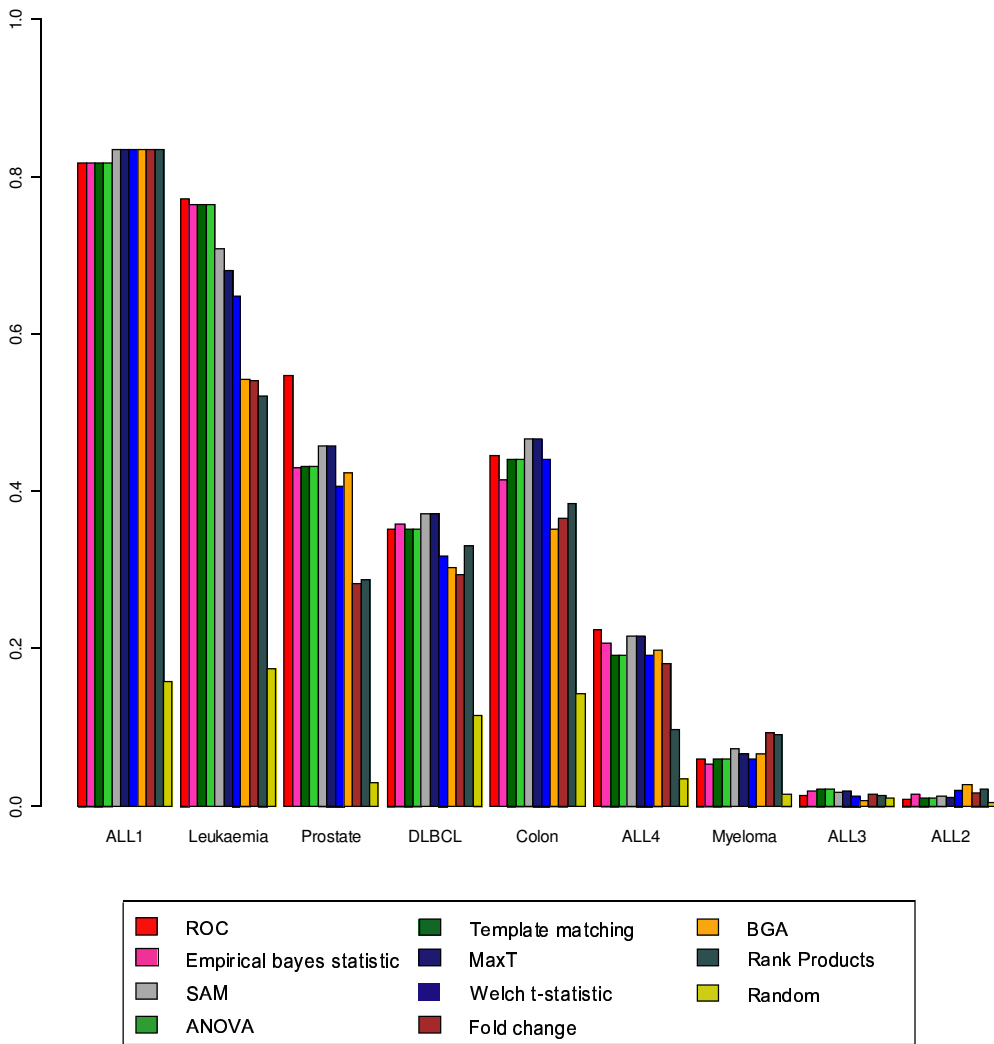

[illegible]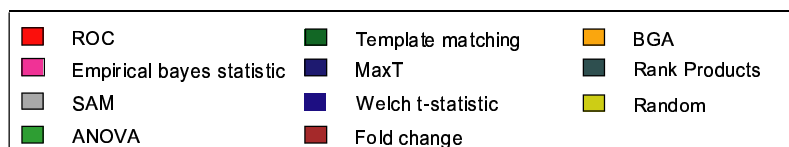

Supplement: Additional File 13 — The RCI scores for each of the individual datasets and individual classification methods where the top 20 genes are used and n = 50% of the samples per class. RCI values showing the success of the top 20 genes, selected by the feature selection methods, to form classifiers which can predict the class of blind test data for each of the 9 datasets. These figures show the results for each of the classification methods when a datasets split equally into training and test sets is used. [file 1471-2105-7-359-S13.pdf]

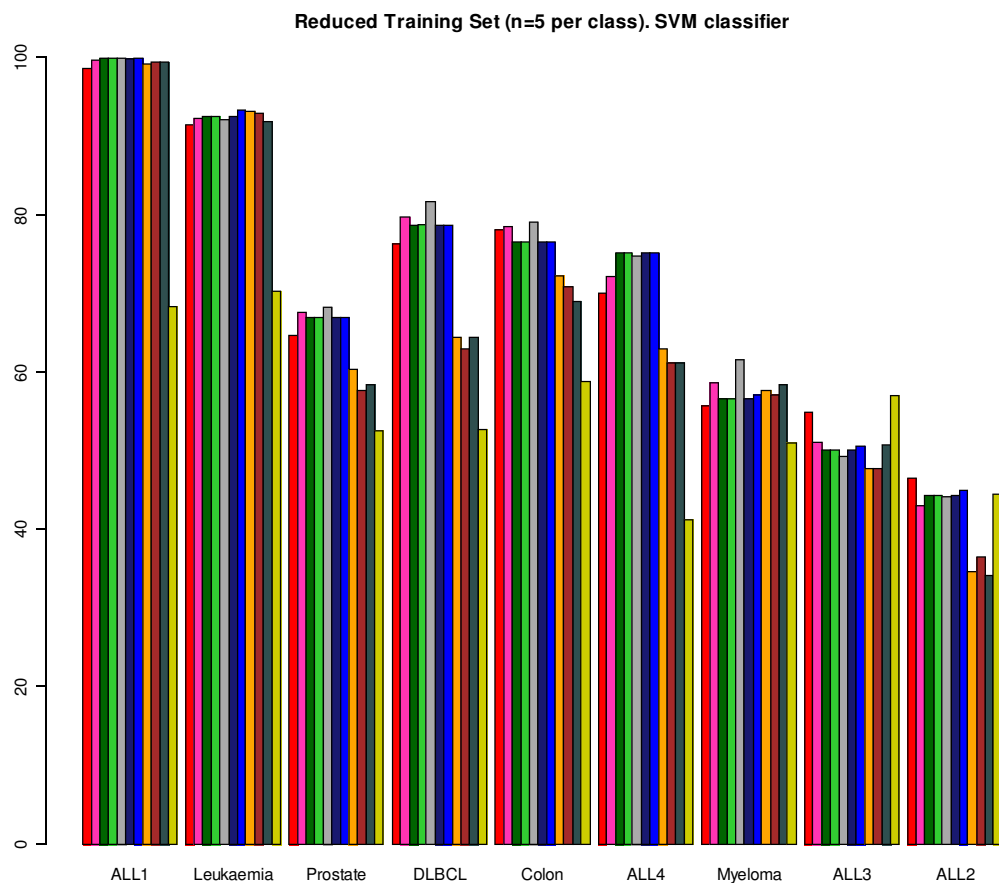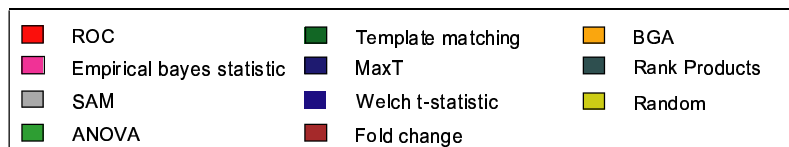

Reduced Training Set (n=5 per class). Naive Bayes classifier

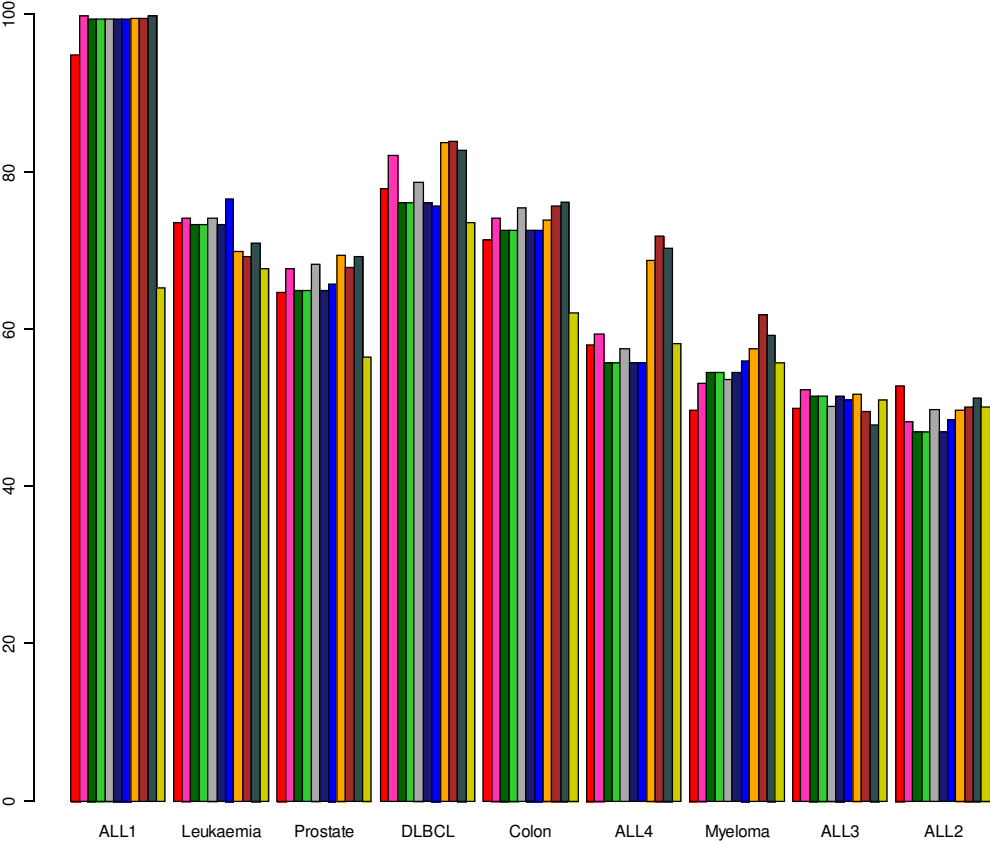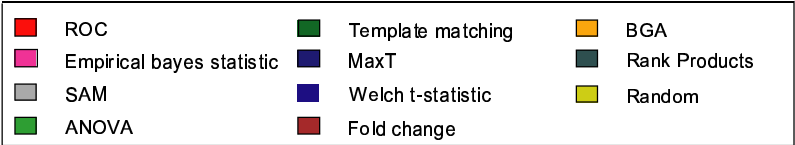

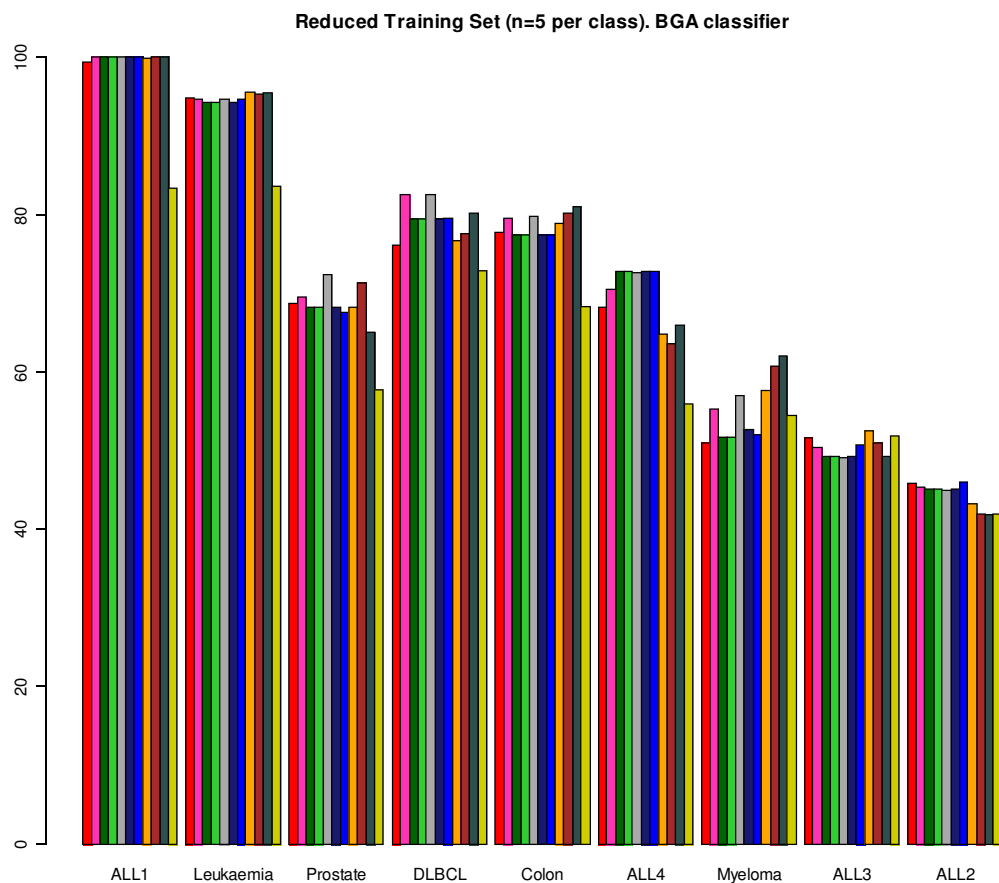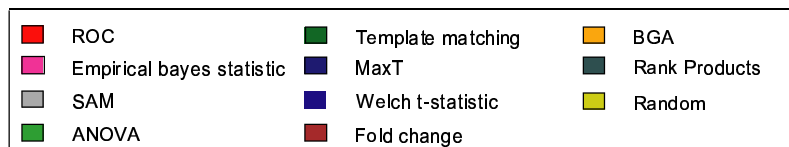

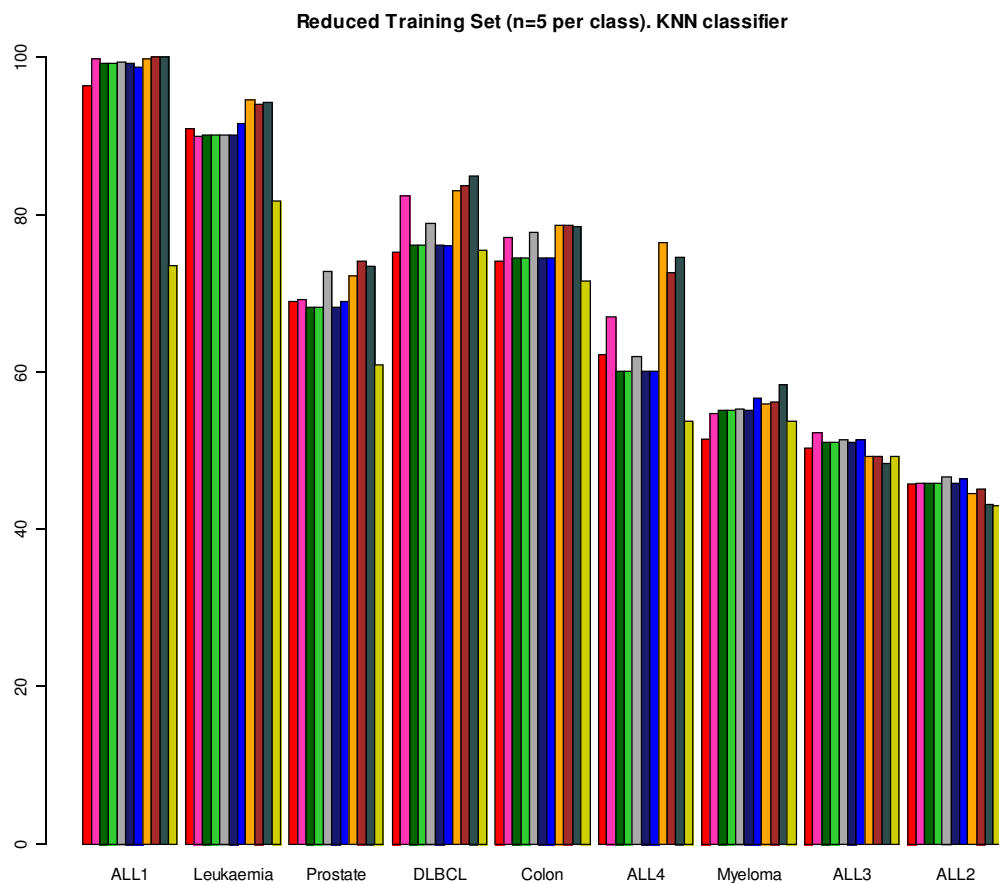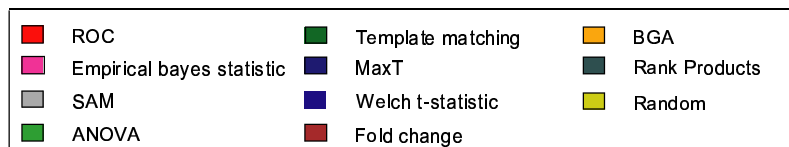

Supplement: Additional File 14 — The percentage accuracy scores for each of the individual datasets and individual classification methods where the top 80 genes are used and n = 5 samples per class. The percentage accuracy of the top 80 genes, selected by the feature selection methods, to form classifiers which can predict the class of blind test data for each of the 9 datasets. These figures show the results for each of the classification methods when a reduced training set of 10 (5 from each class) is used. [file 1471-2105-7-359-S14.pdf]

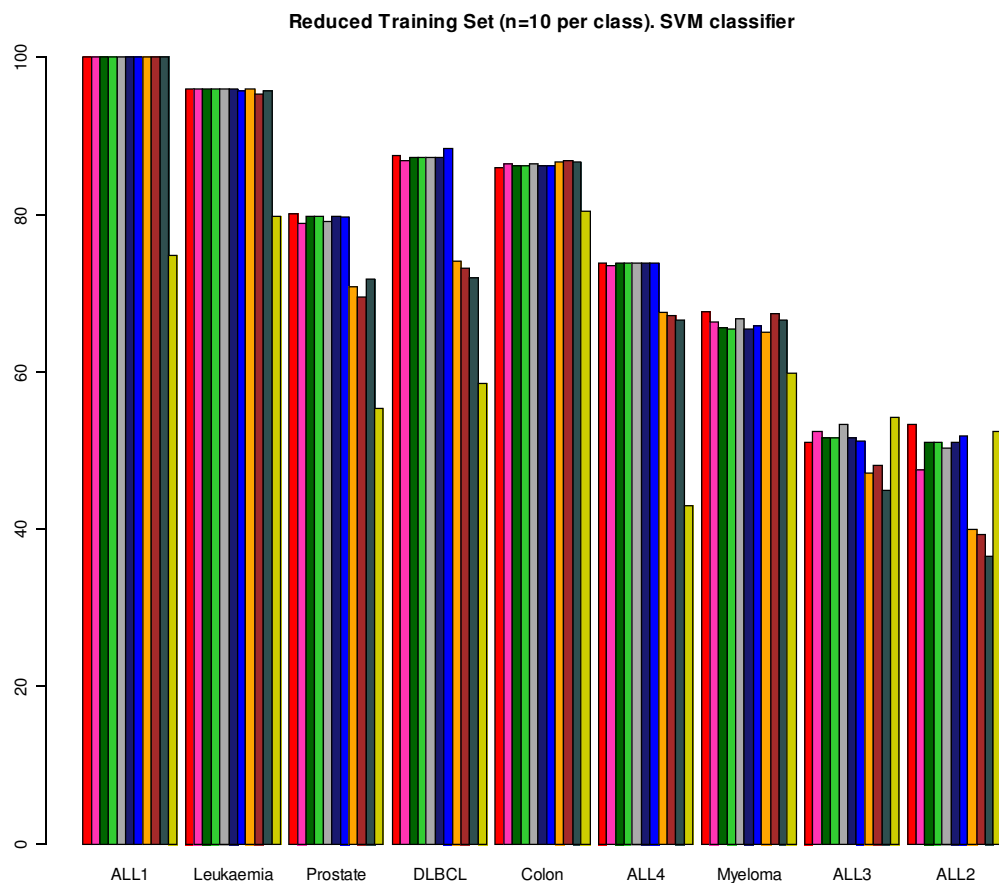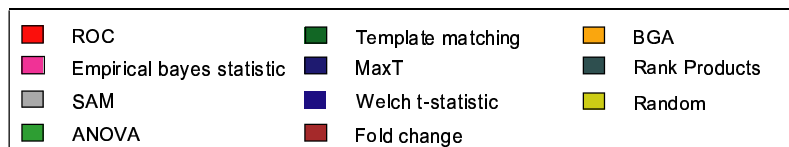

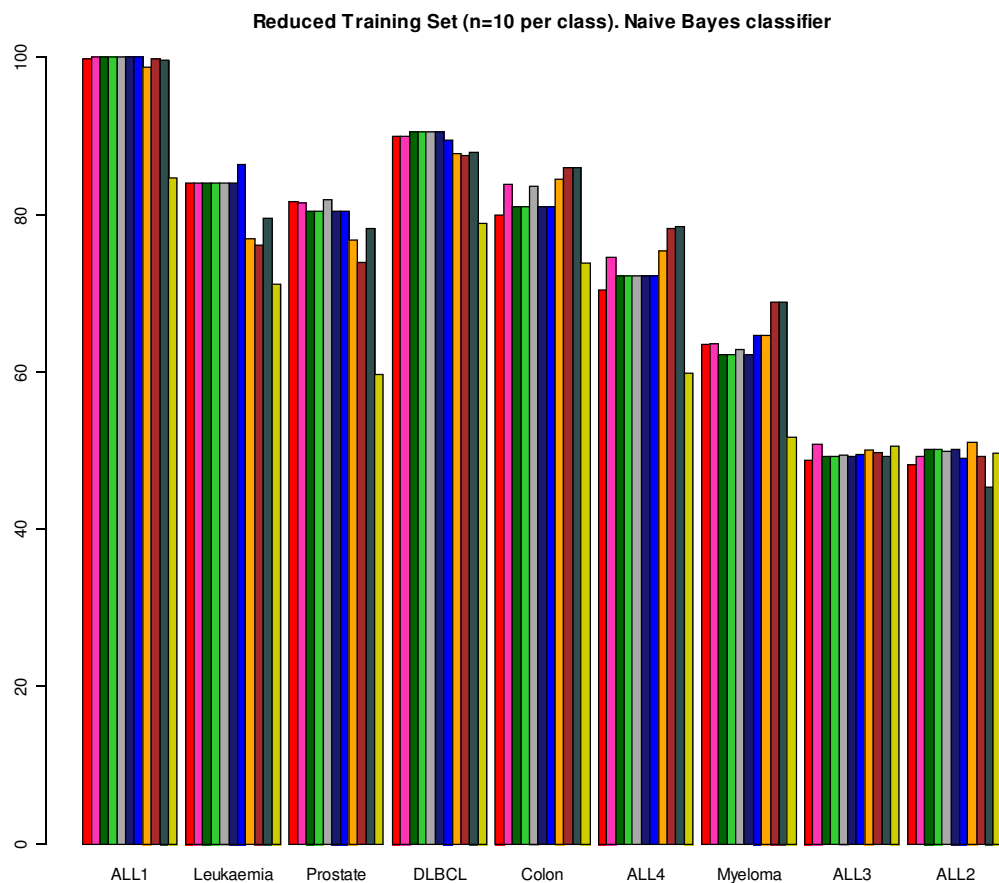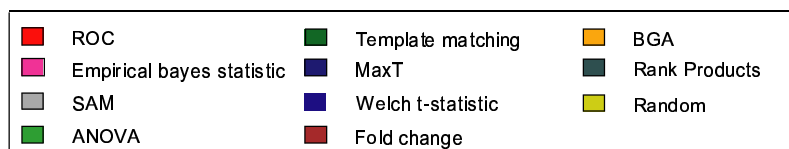

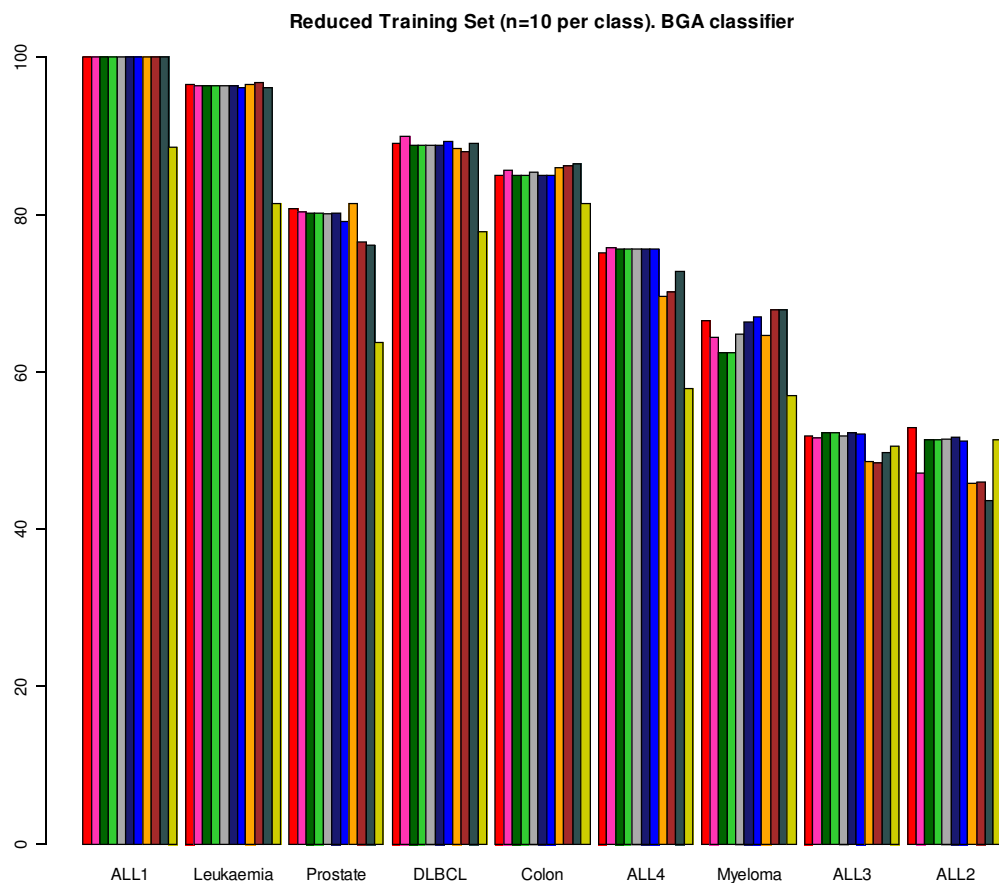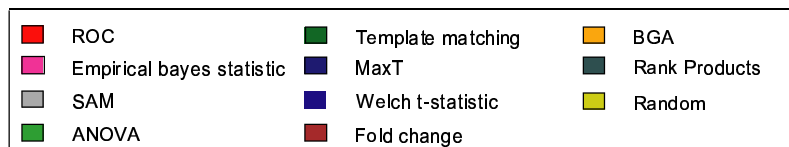

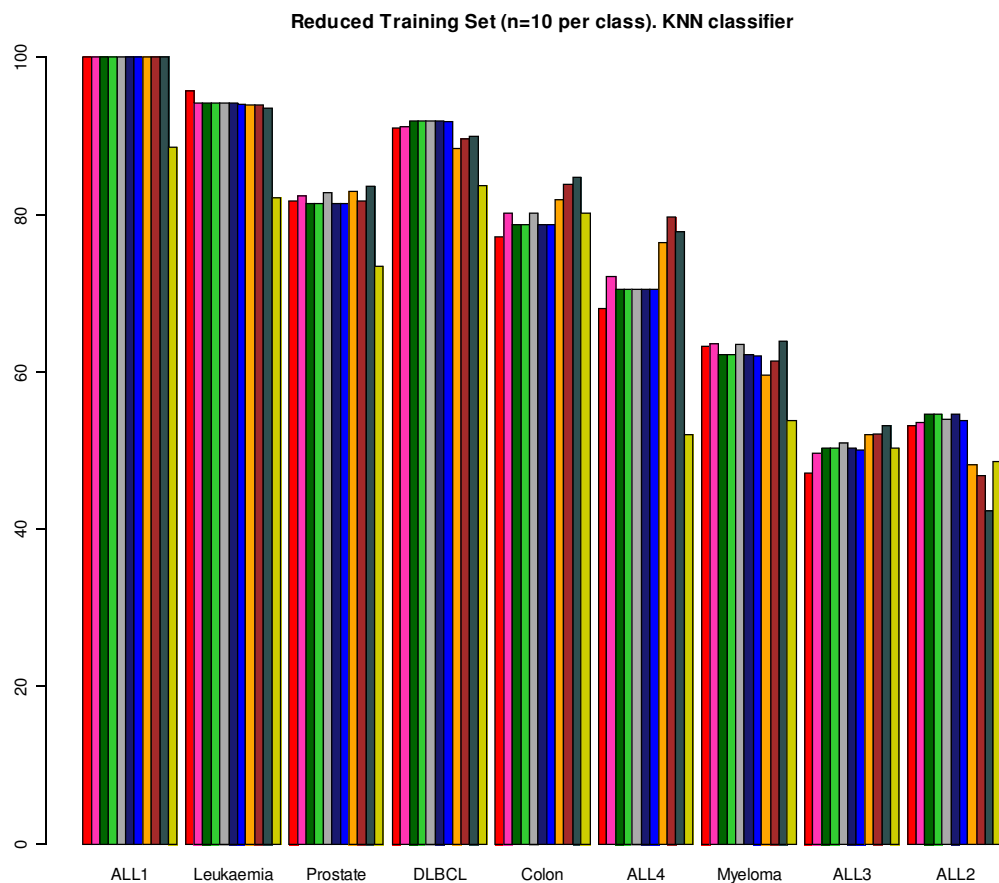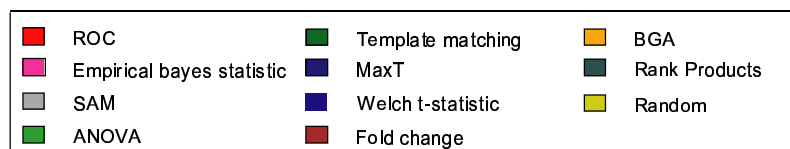

Supplement: Additional File 15 — The percentage accuracy scores for each of the individual datasets and individual classification methods where the top 80 genes are used and n = 10 samples per class. The percentage accuracy of the top 80 genes, selected by the feature selection methods, to form classifiers which can predict the class of blind test data for each of the 9 datasets. These figures show the results for each of the classification methods when a reduced training set of 20 (10 from each class) is used. [file 1471-2105-7-359-S15.pdf]

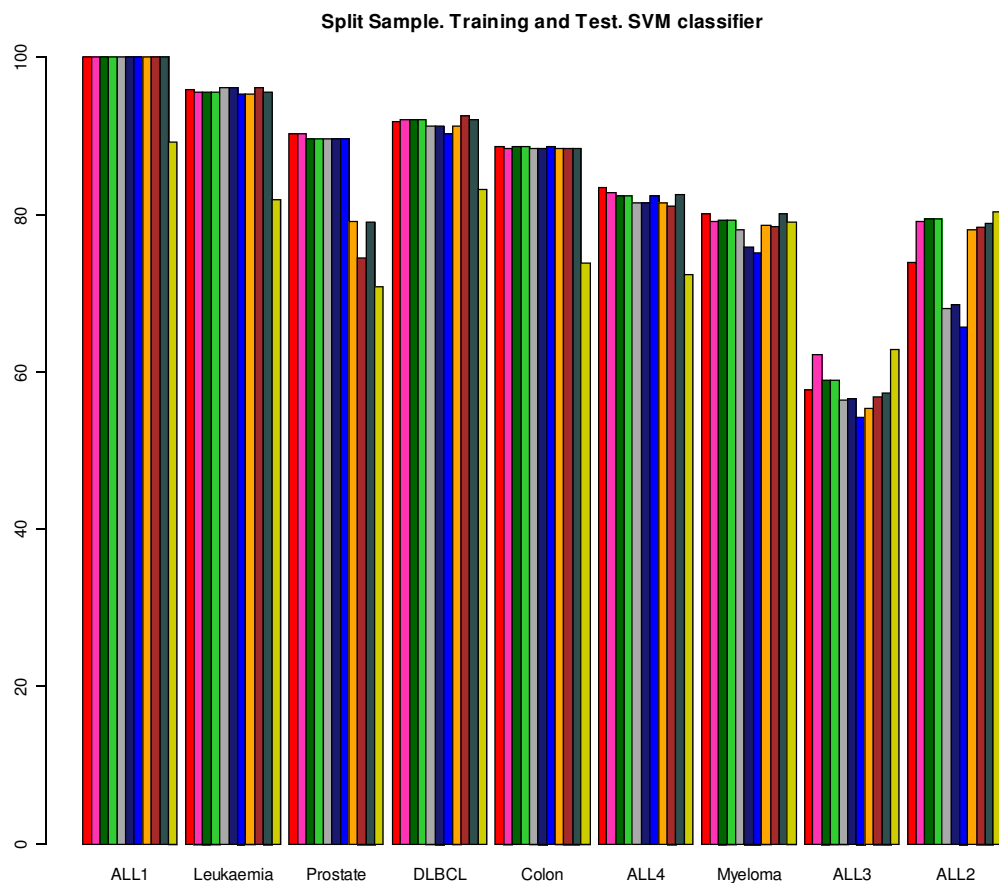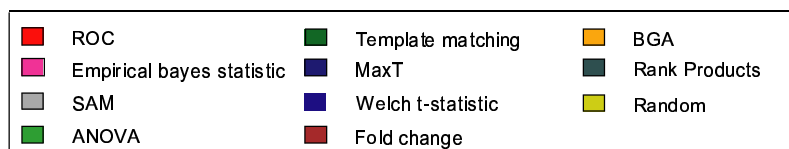

Split Sample. Training and Test. Naive Bayes classifier

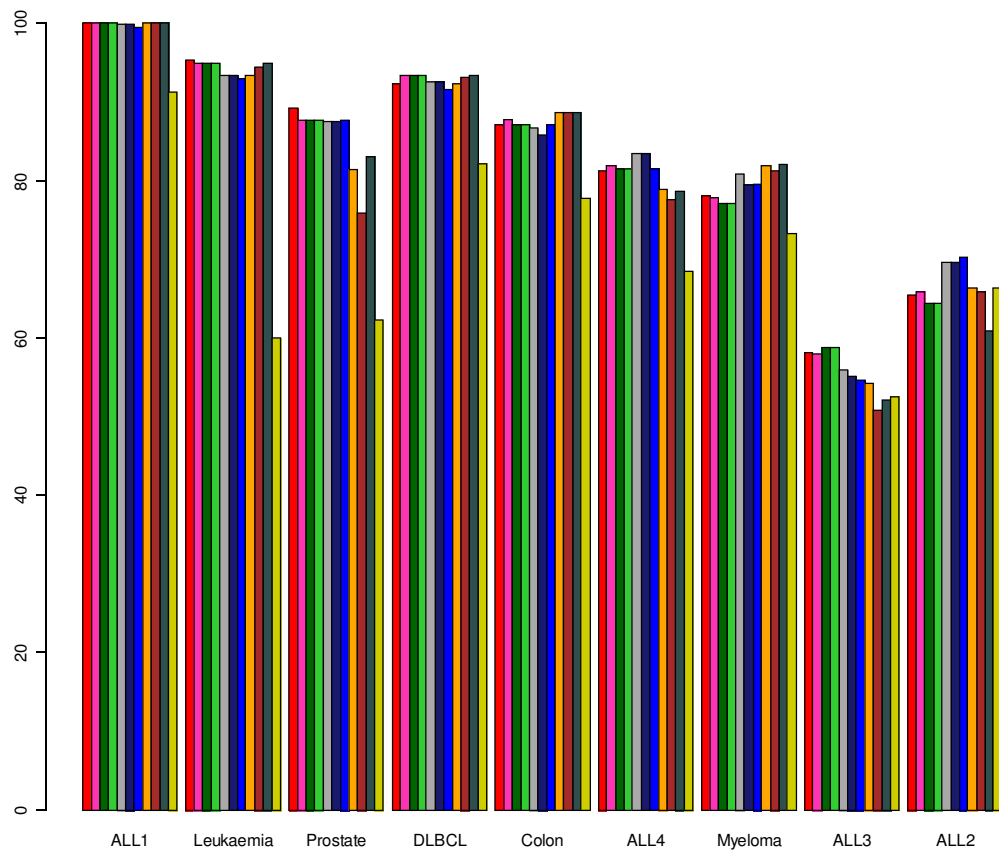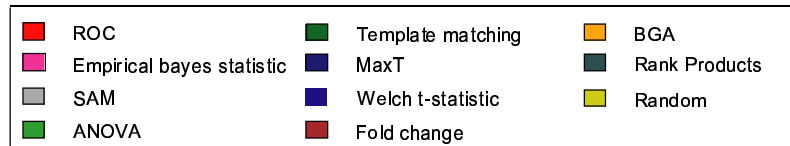

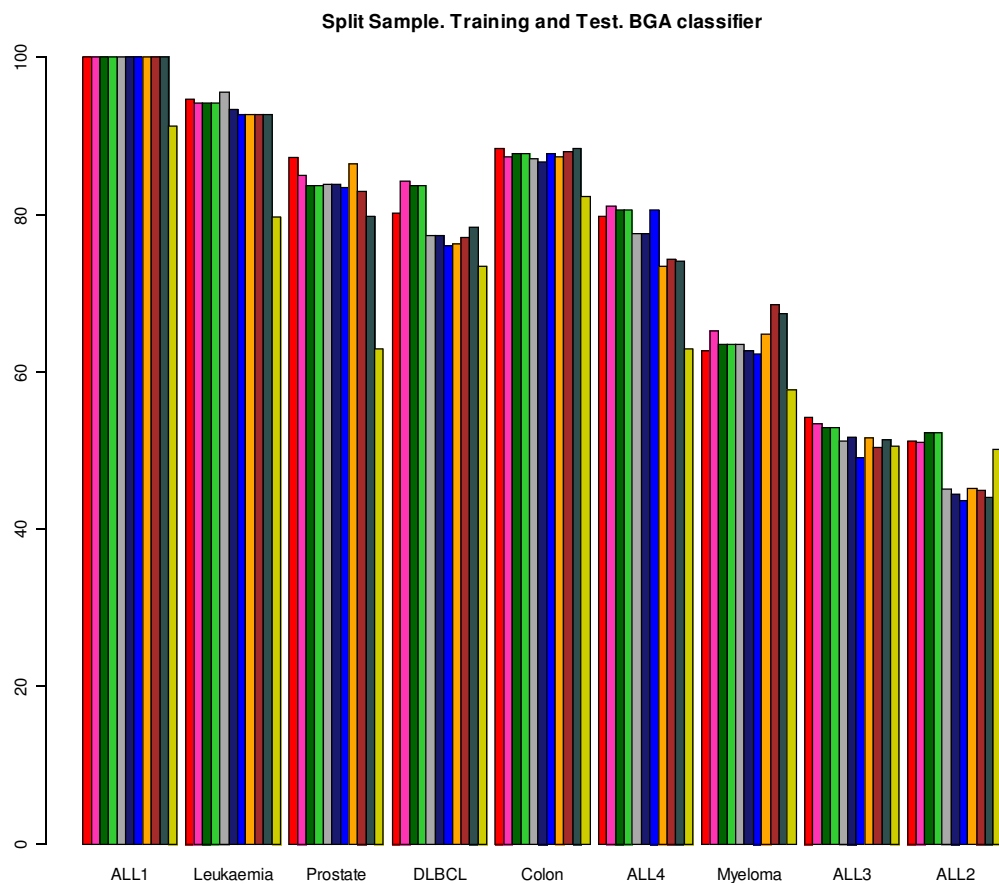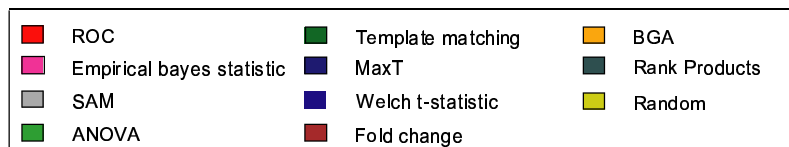

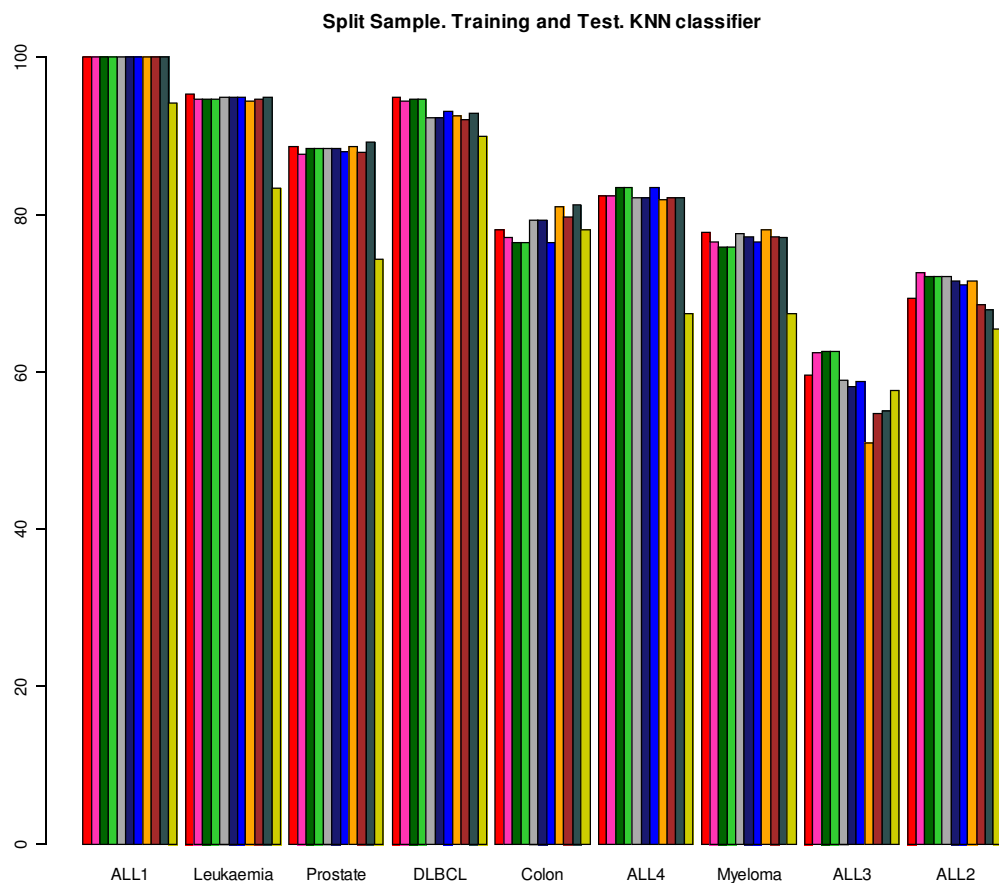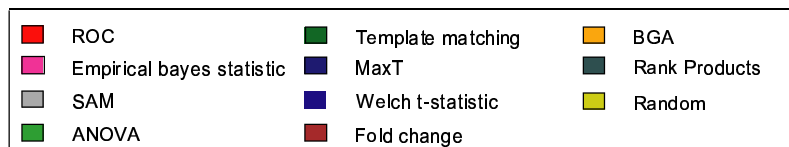

Supplement: Additional File 16 — The percentage accuracy scores for each of the individual datasets and individual classification methods where the top 80 genes are used and n = 50% of the samples per class. The percentage accuracy of the top 80 genes, selected by the feature selection methods, to form classifiers which can predict the class of blind test data for each of the 9 datasets. These figures show the results for each of the classification methods when a datasets split equally into training and test sets is used. [file 1471-2105-7-359-S16.pdf]

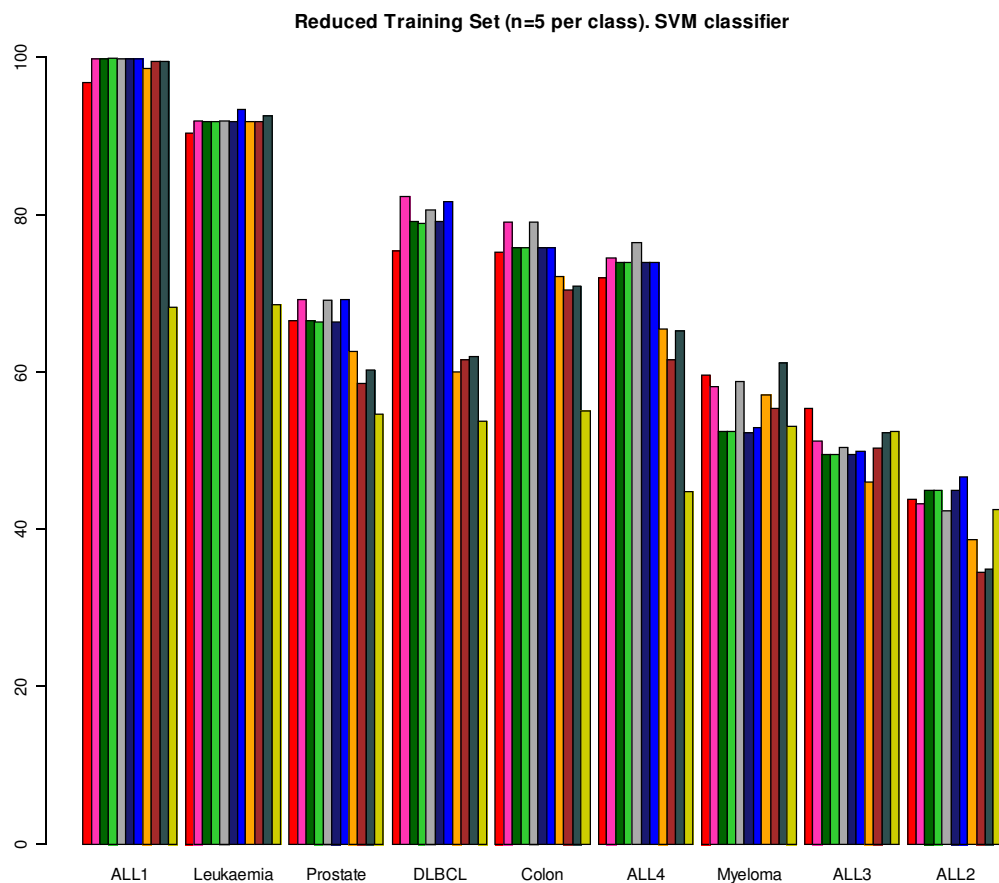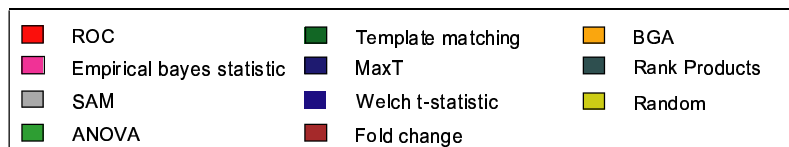

Reduced Training Set (n=5 per class). Naive Bayes classifier

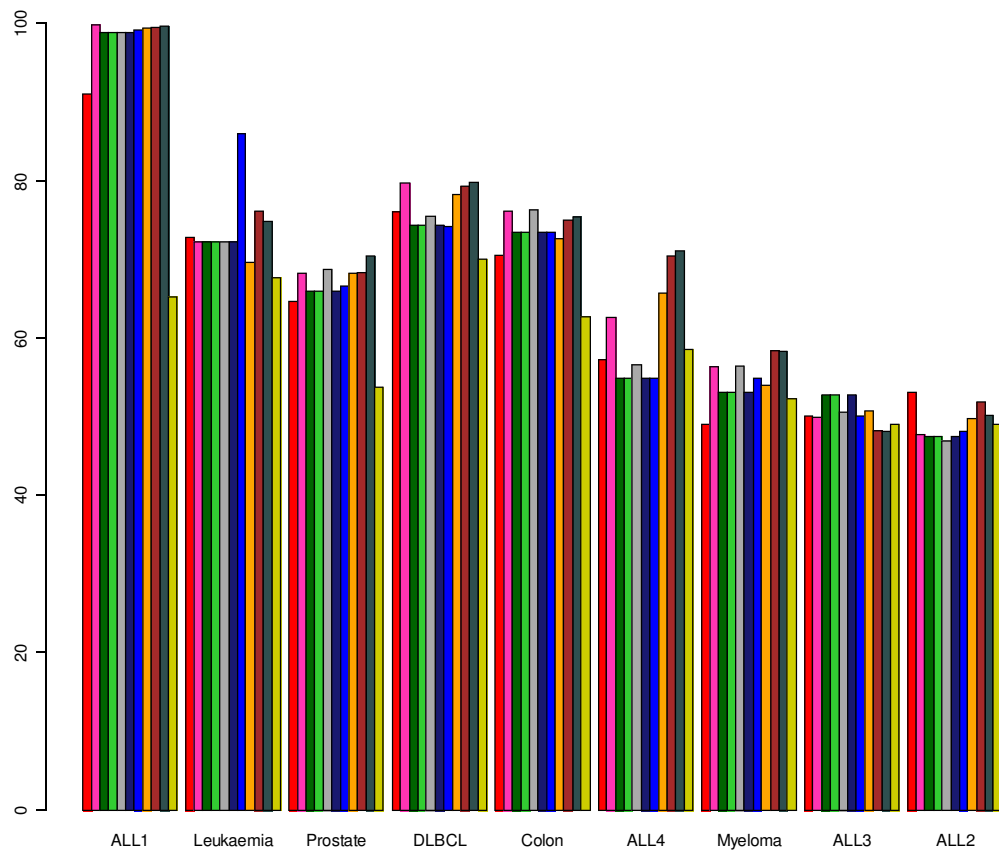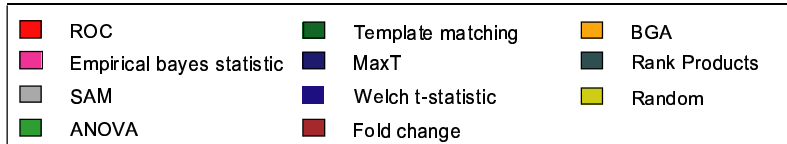

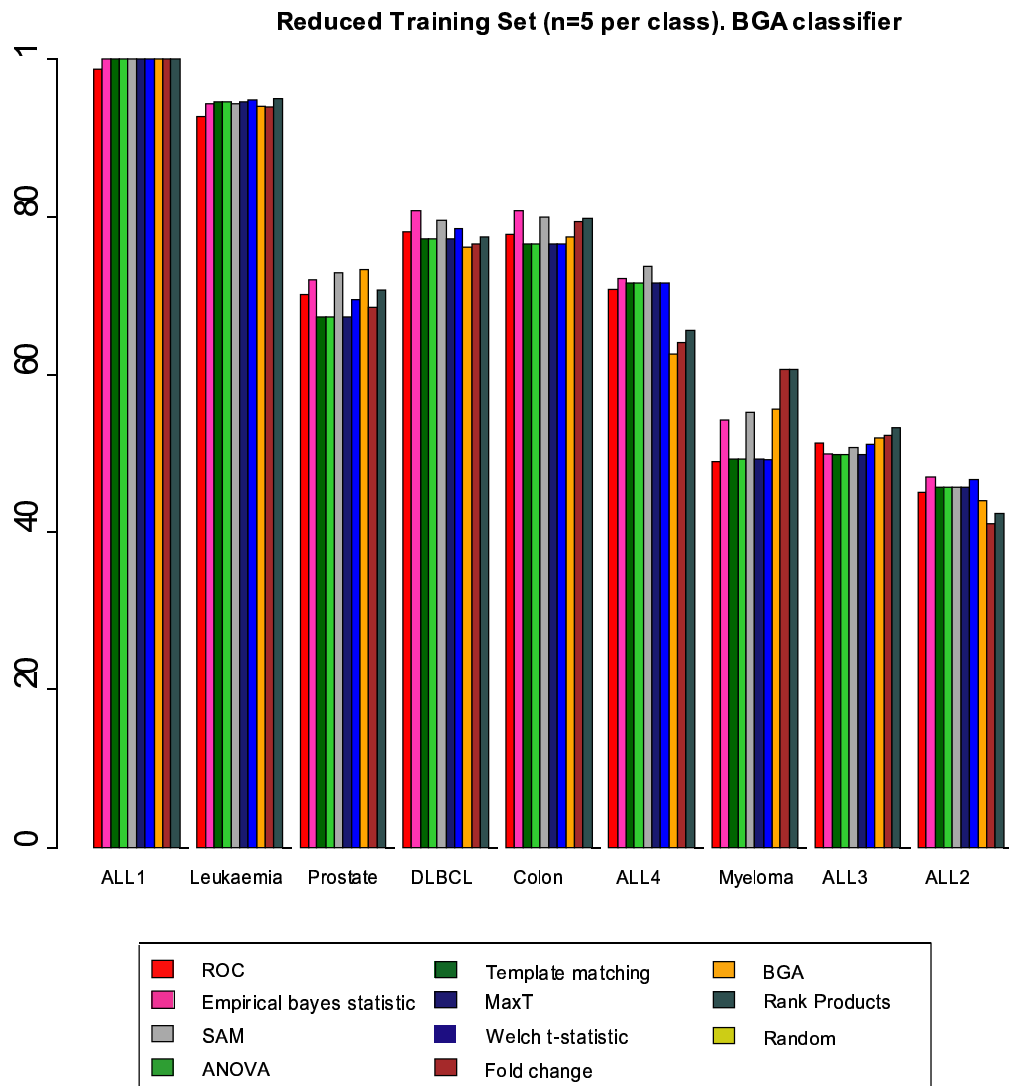

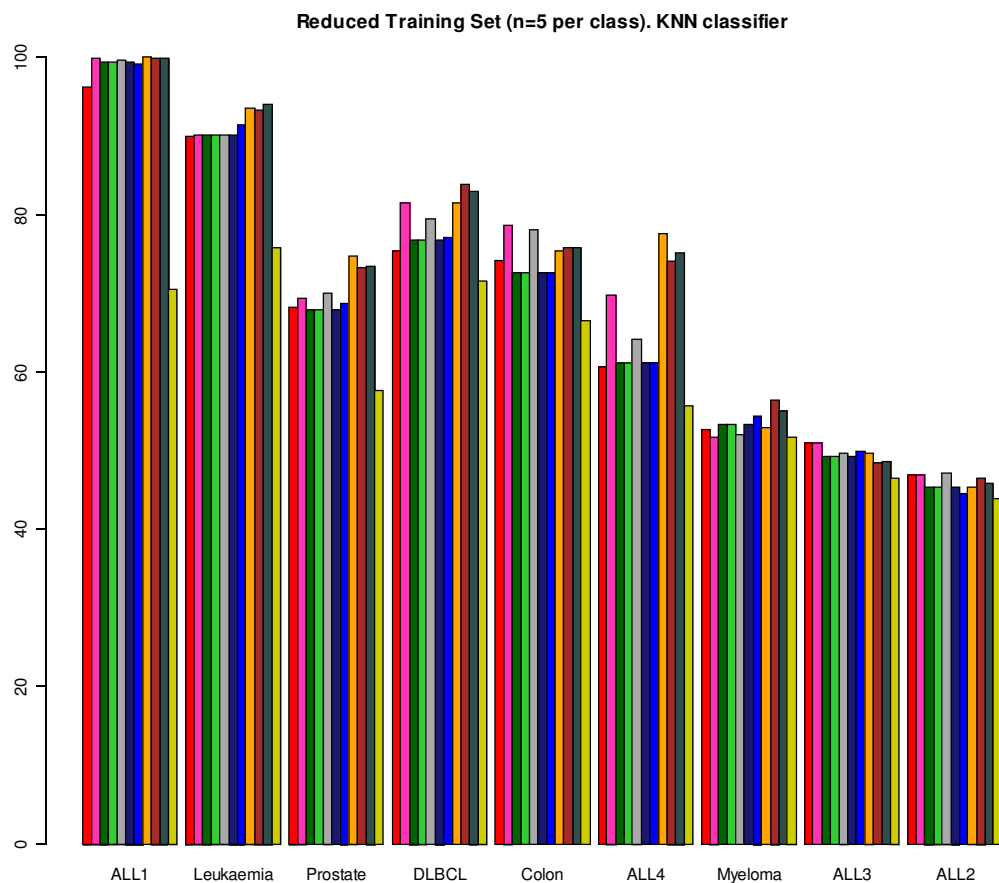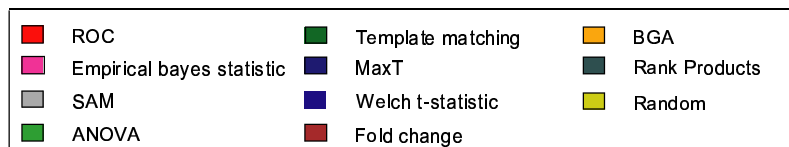

Supplement: Additional File 17 — The percentage accuracy scores for each of the individual datasets and individual classification methods where the top 40 genes are used and n = 5 samples per class. The percentage accuracy of the top 40 genes, selected by the feature selection methods, to form classifiers which can predict the class of blind test data for each of the 9 datasets. These figures show the results for each of the classification methods when a reduced training set of 10 (5 from each class) is used. [file 1471-2105-7-359-S17.pdf]

Reduced Training Set (n=10 per class). Naive Bayes classifier

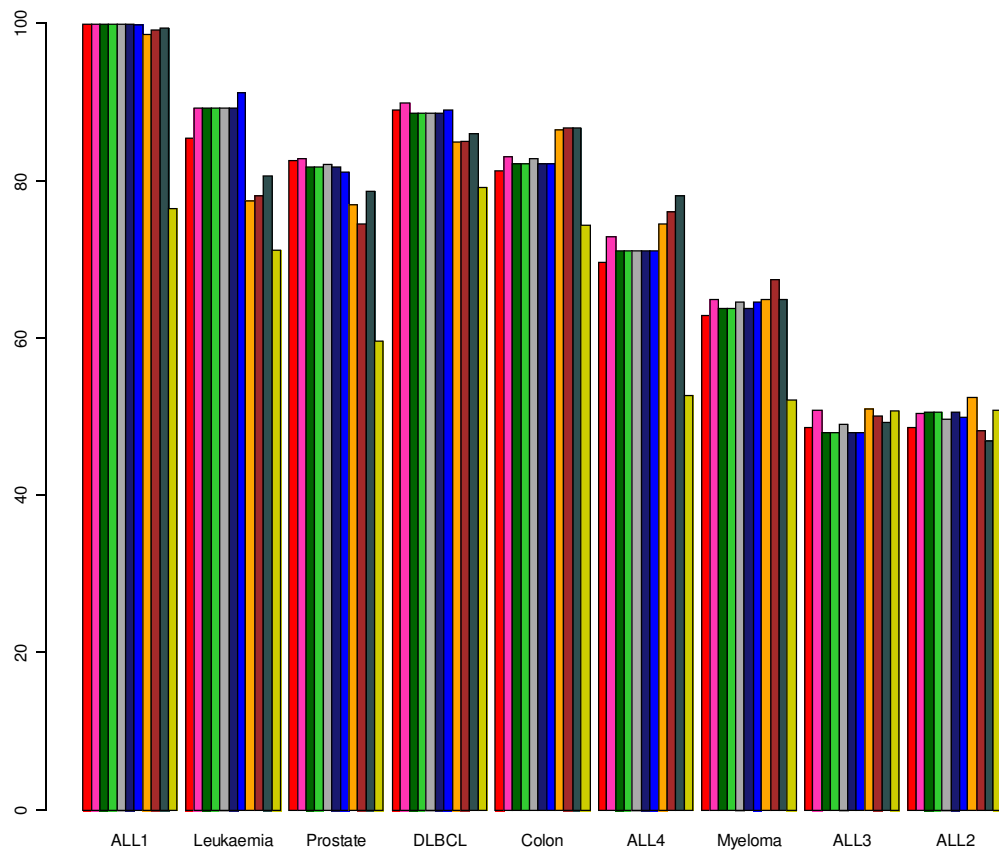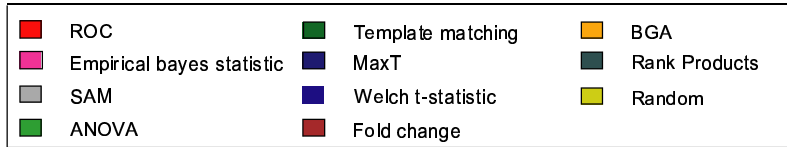

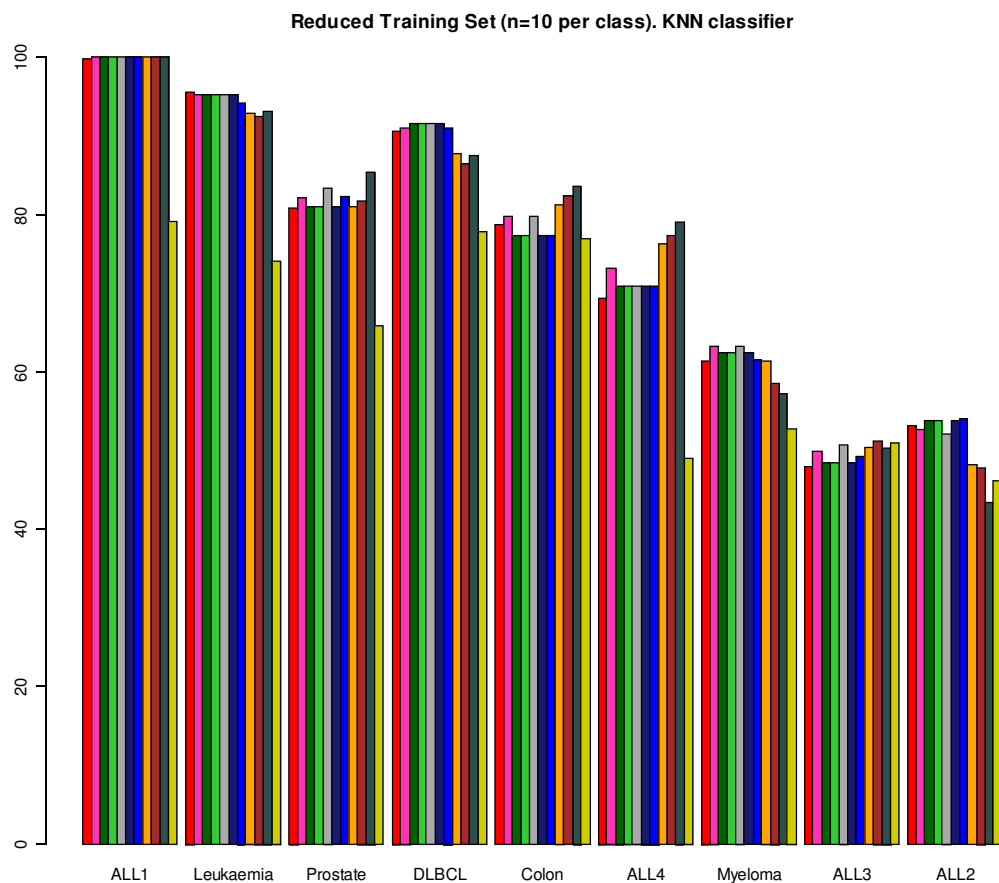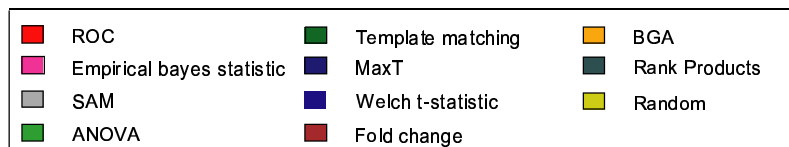

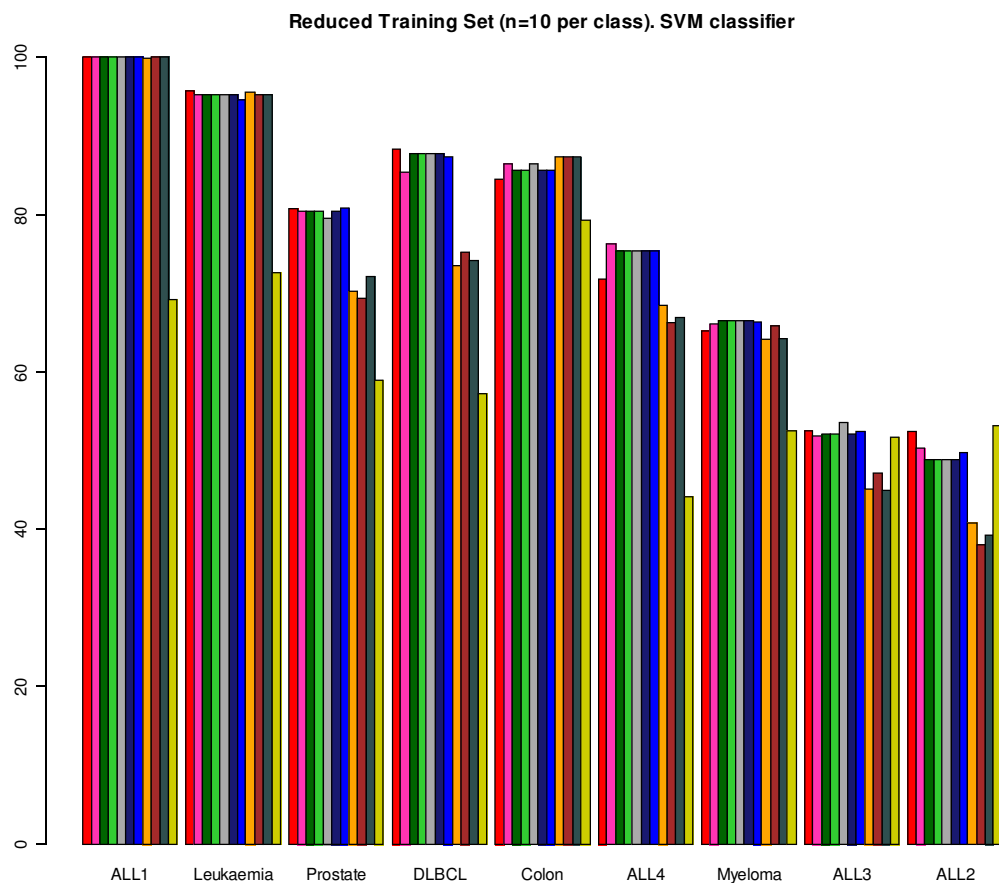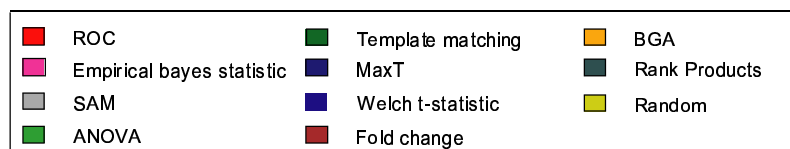

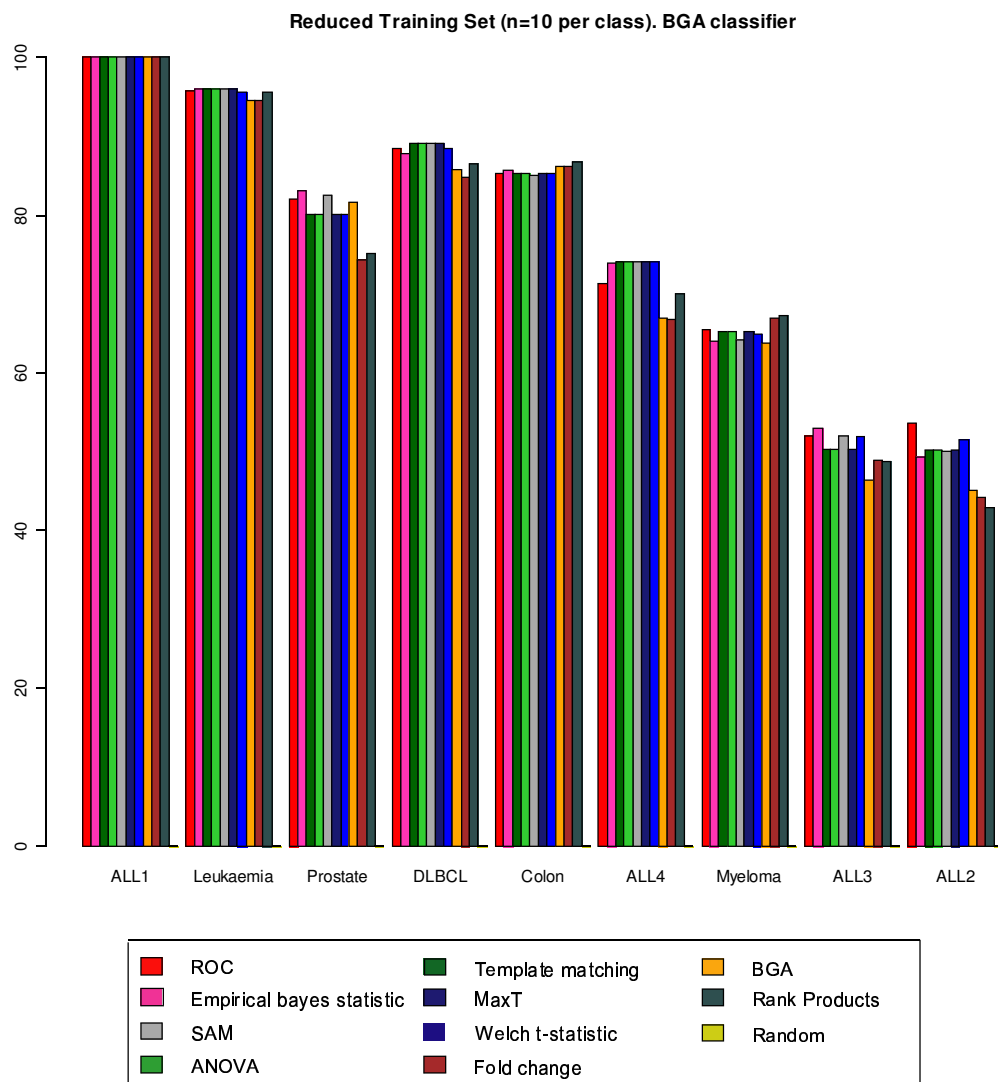

Supplement: Additional File 18 — The percentage accuracy scores for each of the individual datasets and individual classification methods where the top 40 genes are used and n = 10 samples per class. The percentage accuracy of the top 40 genes, selected by the feature selection methods, to form classifiers which can predict the class of blind test data for each of the 9 datasets. These figures show the results for each of the classification methods when a reduced training set of 20 (10 from each class) is used. [file 1471-2105-7-359-S18.pdf]

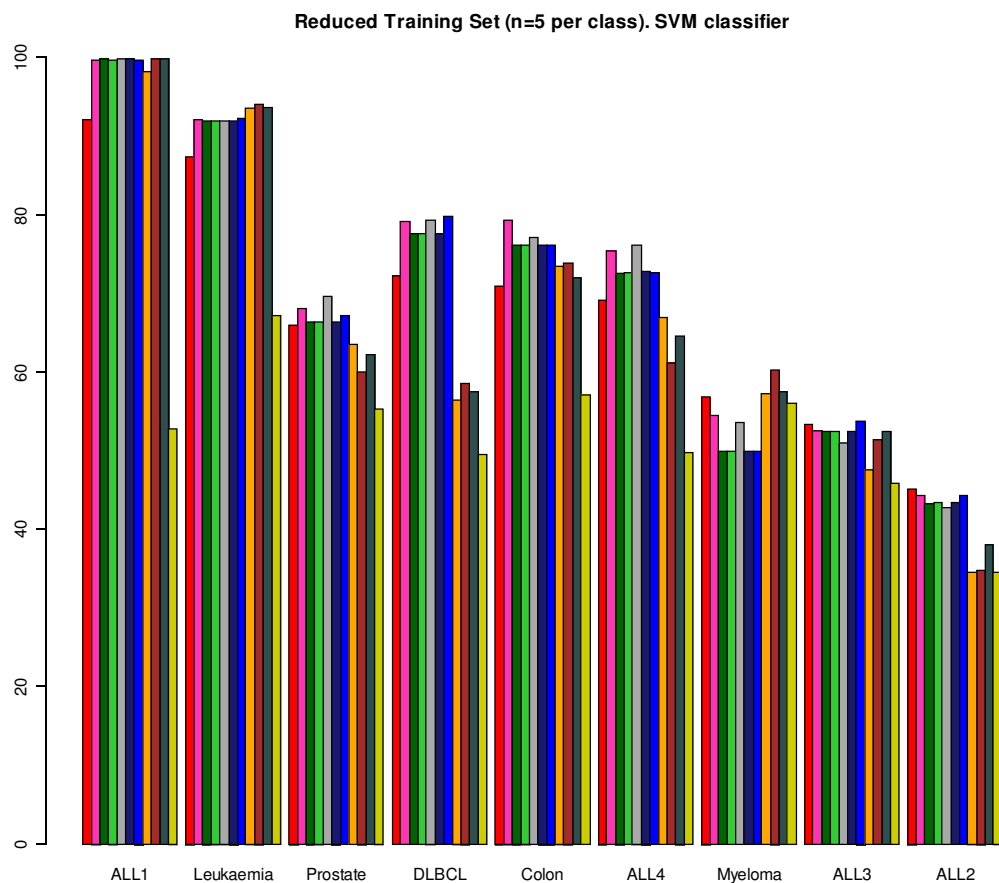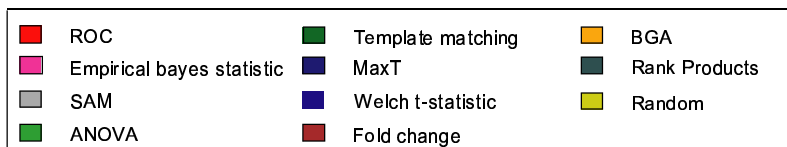

Reduced Training Set (n=5 per class). Naive Bayes classifier

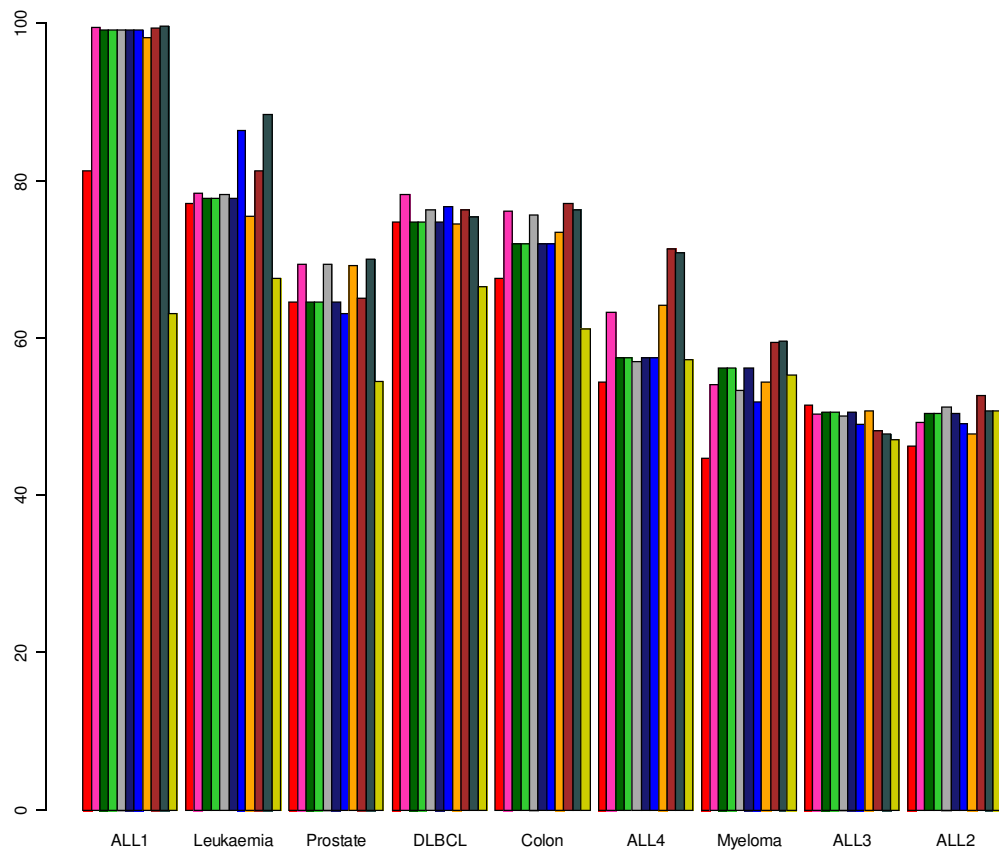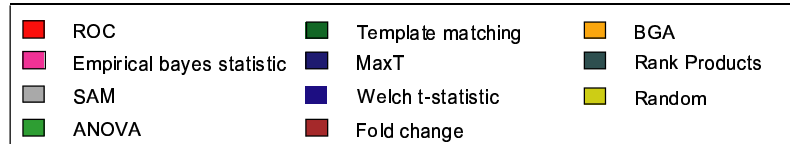

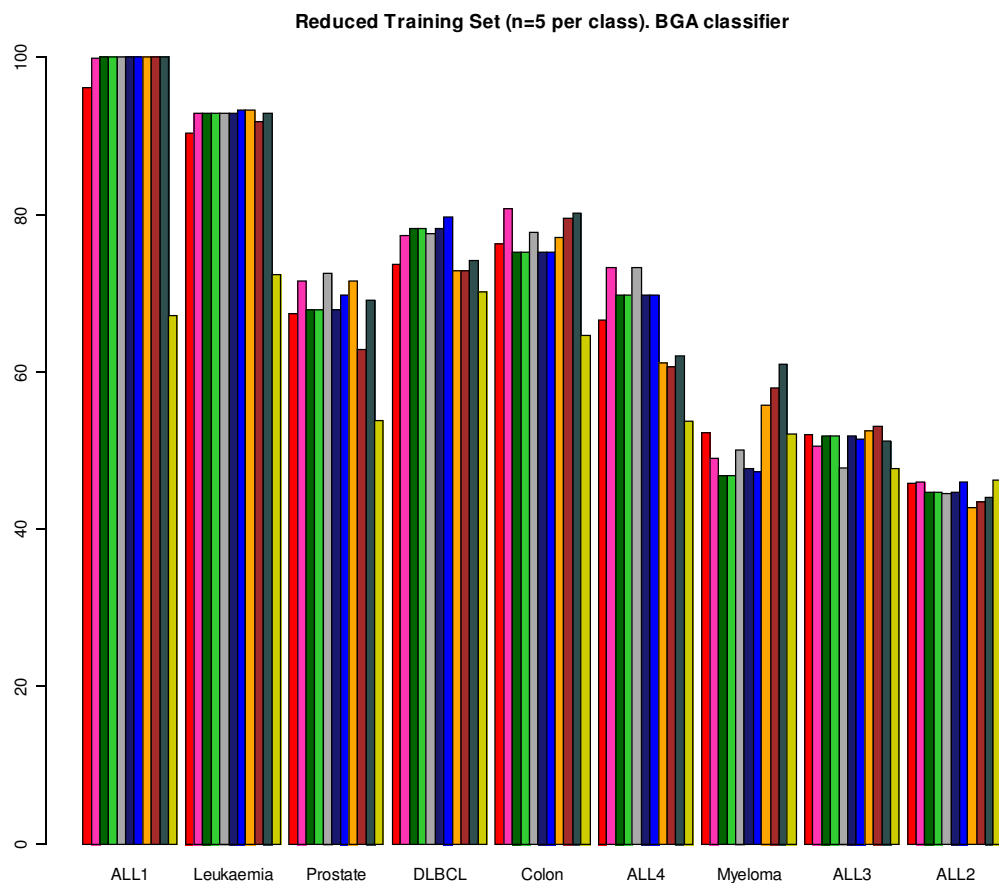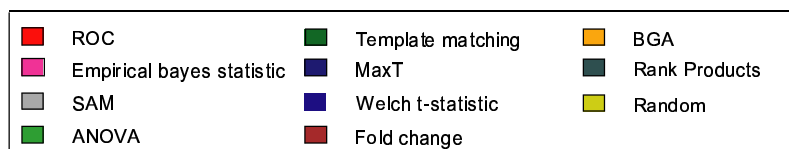

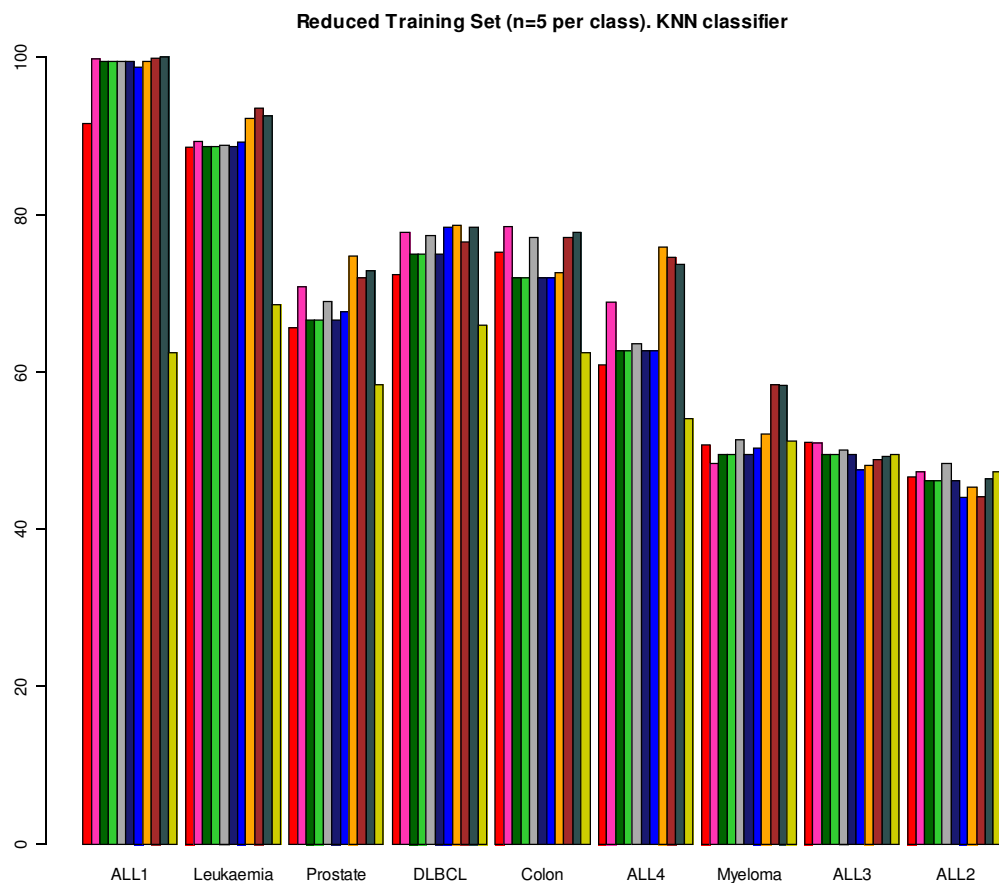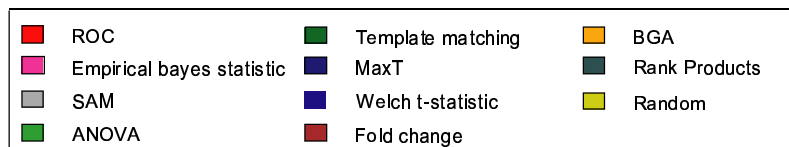

Supplement: Additional File 20 — The percentage accuracy scores for each of the individual datasets and individual classification methods where the top 20 genes are used and n = 5 samples per class. The percentage accuracy of the top 20 genes, selected by the feature selection methods, to form classifiers which can predict the class of blind test data for each of the 9 datasets. These figures show the results for each of the classification methods when a reduced training set of 10 (5 from each class) is used. [file 1471-2105-7-359-S20.pdf]

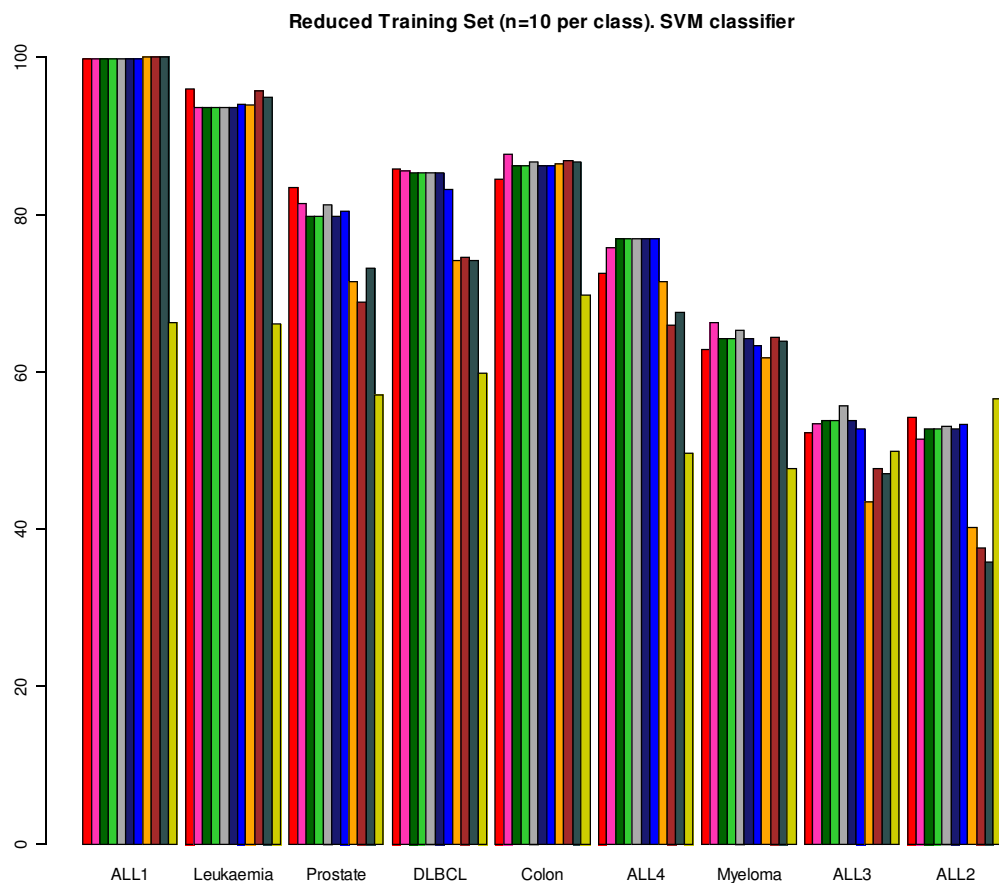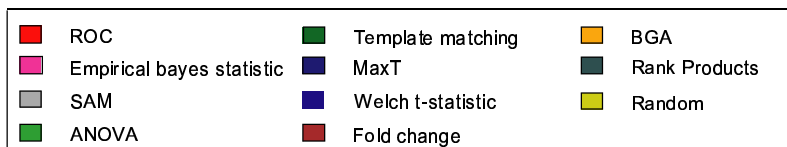

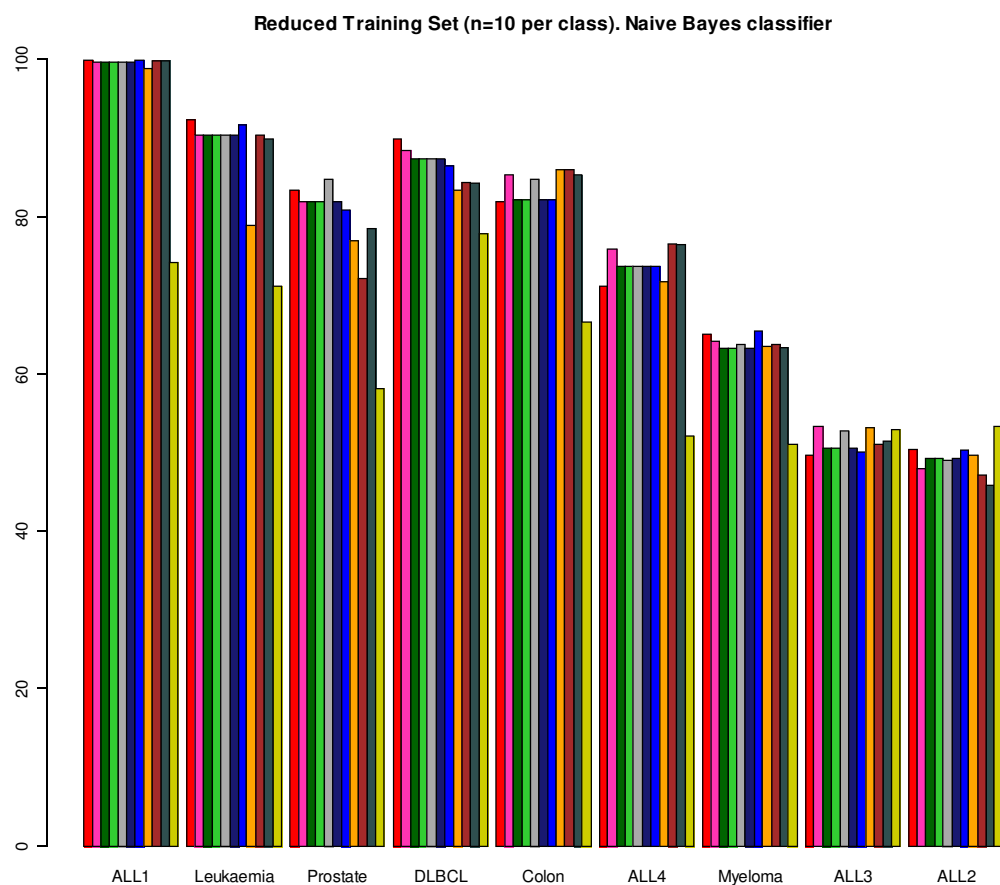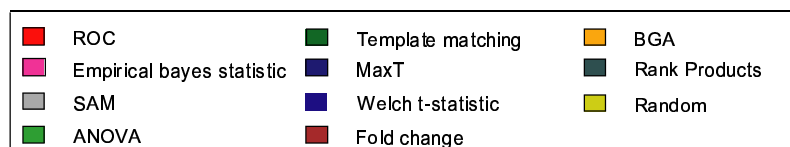

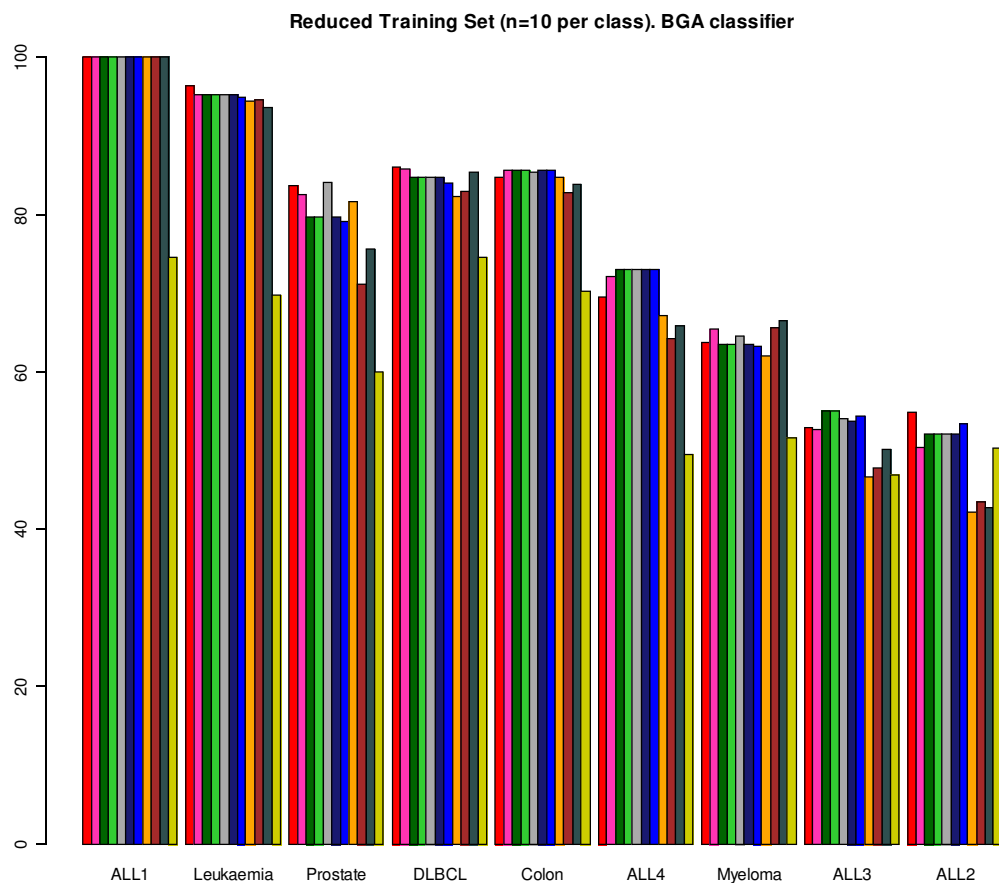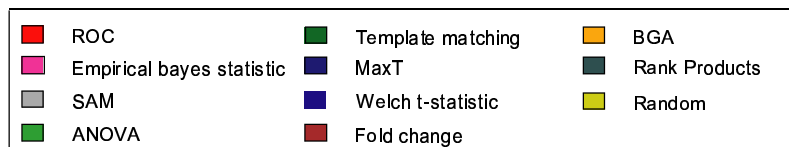

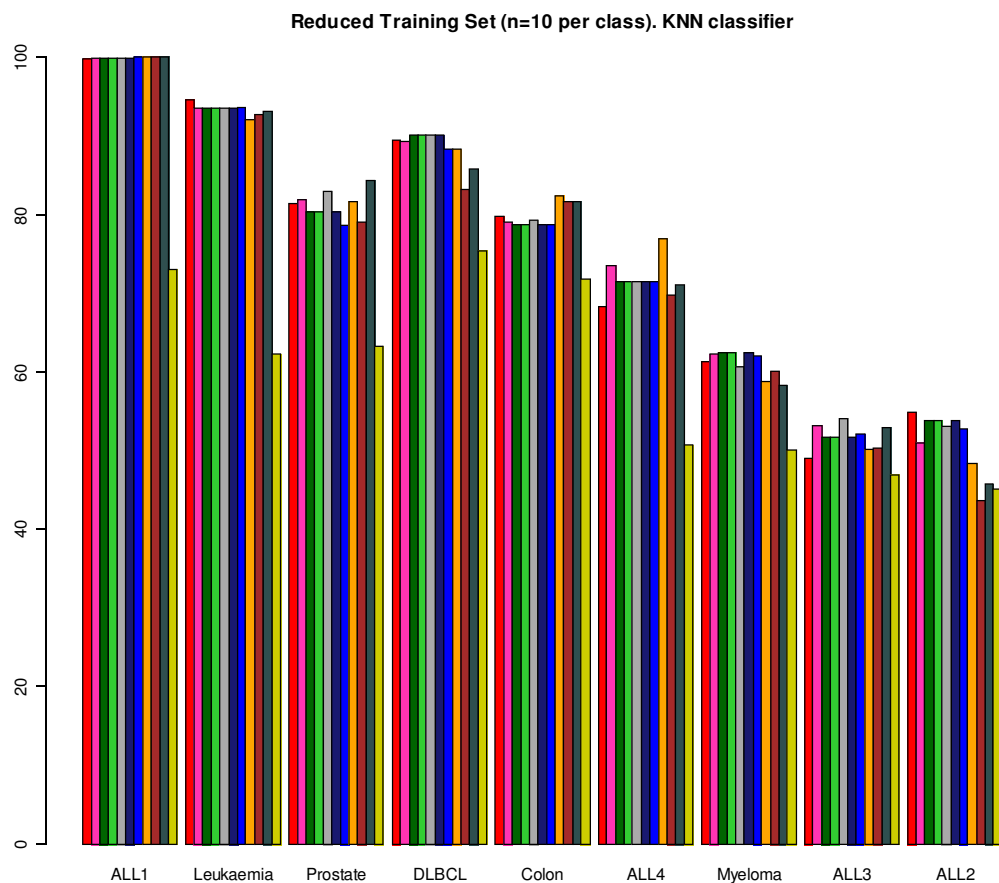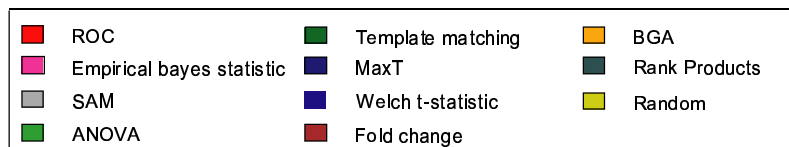

Supplement: Additional File 21 — The percentage accuracy scores for each of the individual datasets and individual classification methods where the top 20 genes are used and n = 10 samples per class. The percentage accuracy of the top 20 genes, selected by the feature selection methods, to form classifiers which can predict the class of blind test data for each of the 9 datasets. These figures show the results for each of the classification methods when a reduced training set of 20 (10 from each class) is used. [file 1471-2105-7-359-S21.pdf]
