# Supplementary material for: Comparison and evaluation of methods for generating differentially expressed gene lists from microarray data
Source: BMC Bioinformatics. 2006 Jul 26;7:359. doi: 10.1186/1471-2105-7-359 (PMC1544358; doi:10.1186/1471-2105-7-359)
Supplement: Additional File 22 — The percentage accuracy scores for each of the individual datasets and individual classification methods where the top 20 genes are used and n = 50% of the samples per class. The percentage accuracy of the top 20 genes, selected by the feature selection methods, to form classifiers which can predict the class of blind test data for each of the 9 datasets. These figures show the results for each of the classification methods when a datasets split equally into training and test sets is used. [file 1471-2105-7-359-S22.pdf]

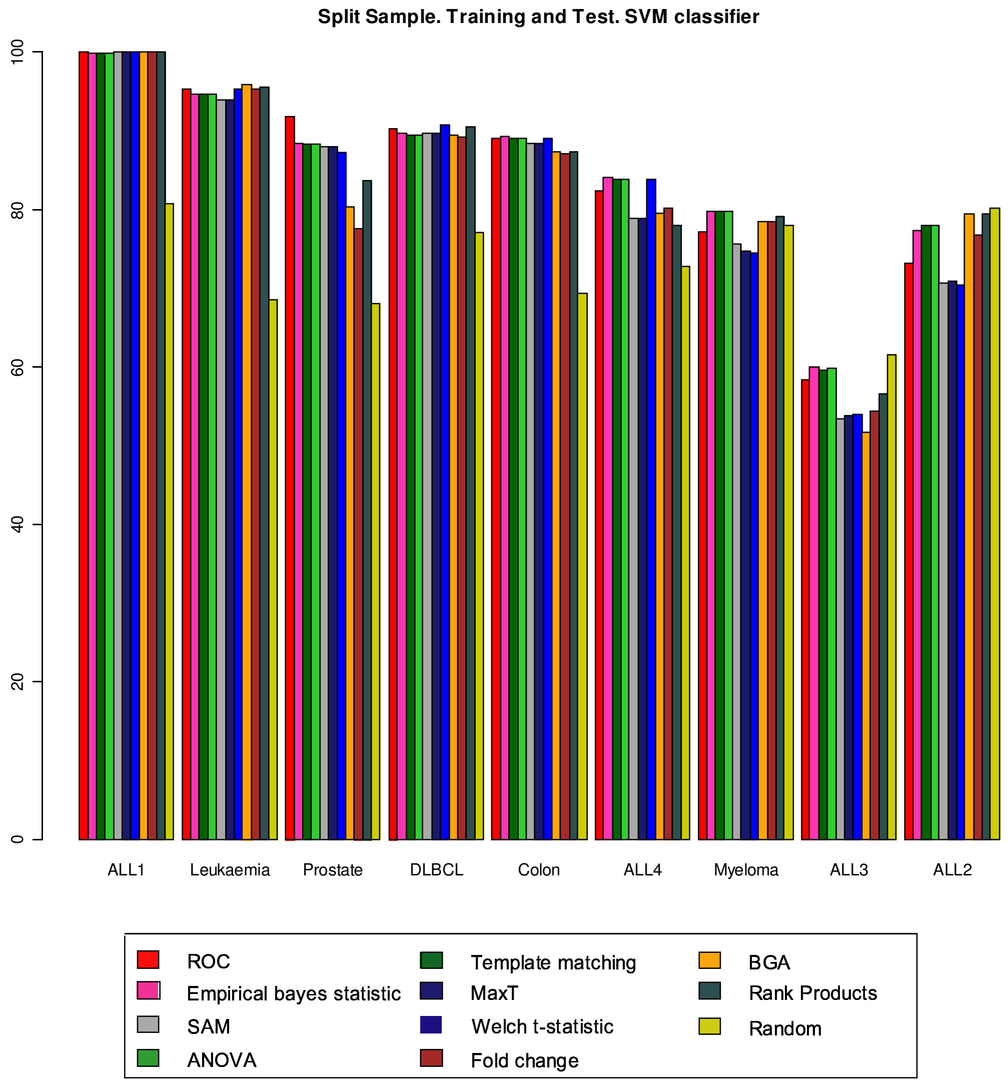

Split Sample. Training and Test. SVM classifier
0
20
40
60
80
100
ALL1
Leukaemia
Prostate
DLBCL
Colon
ALL4
Myeloma
ALL3
ALL2
ROC
Empirical bayes statistic
SAM
ANOVA
Template matching
MaxT
Welch t-statistic
Fold change
BGA
Rank Products
Random

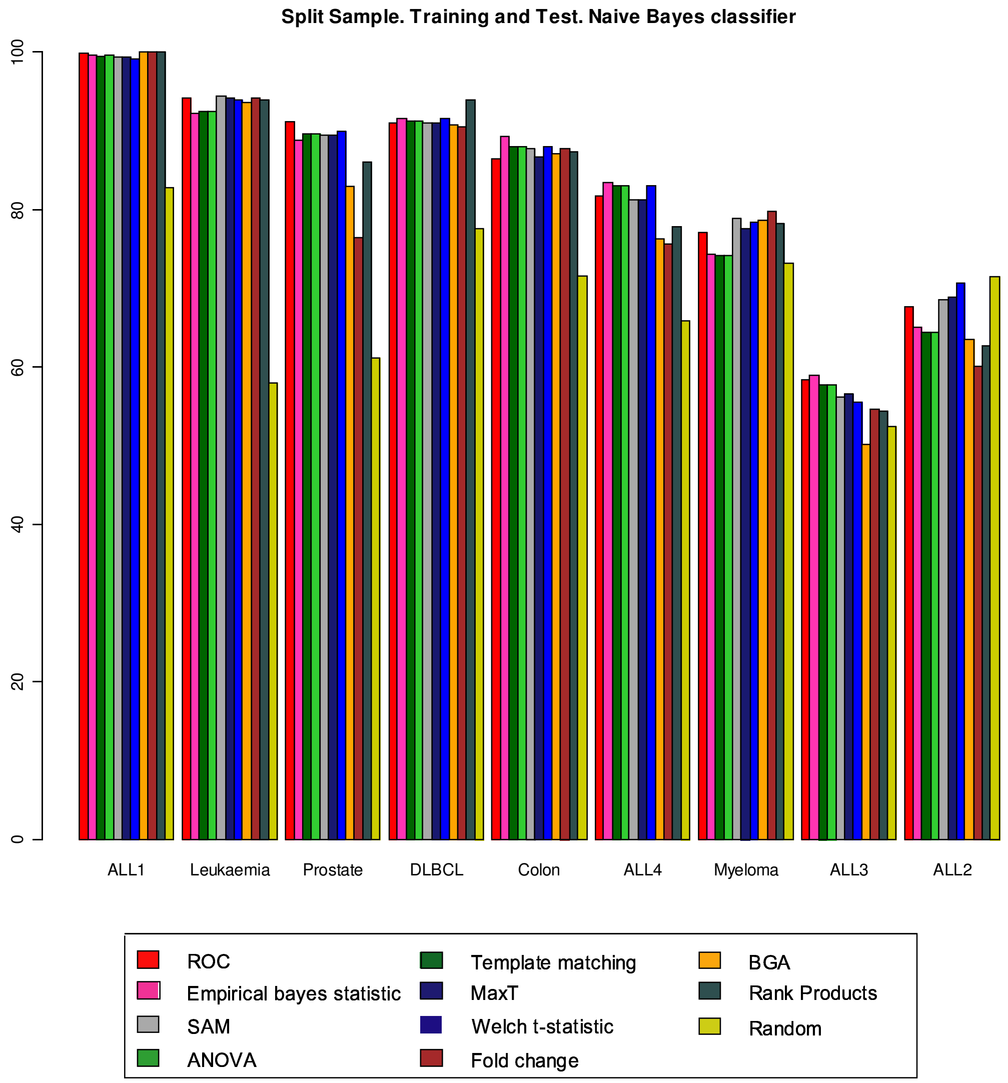

Split Sample. Training and Test. Naive Bayes classifier
0
20
40
60
80
100
ALL1
Leukaemia
Prostate
DLBCL
Colon
ALL4
Myeloma
ALL3
ALL2
ROC
Empirical bayes statistic
SAM
ANOVA
Template matching
MaxT
Welch t-statistic
Fold change
BGA
Rank Products
Random

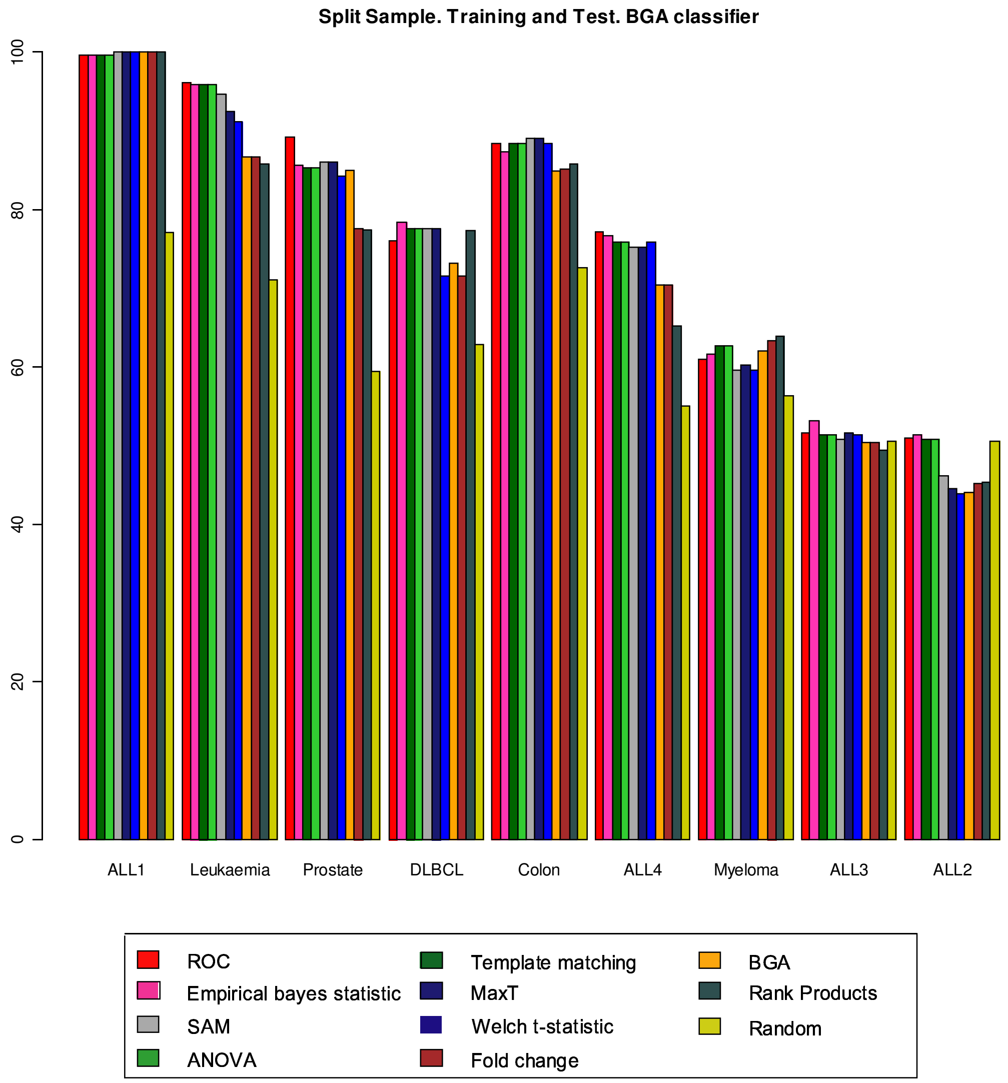

Split Sample. Training and Test. BGA classifier
0
20
40
60
80
100
ALL1
Leukaemia
Prostate
DLBCL
Colon
ALL4
Myeloma
ALL3
ALL2
ROC
Empirical bayes statistic
SAM
ANOVA
Template matching
MaxT
Welch t-statistic
Fold change
BGA
Rank Products
Random

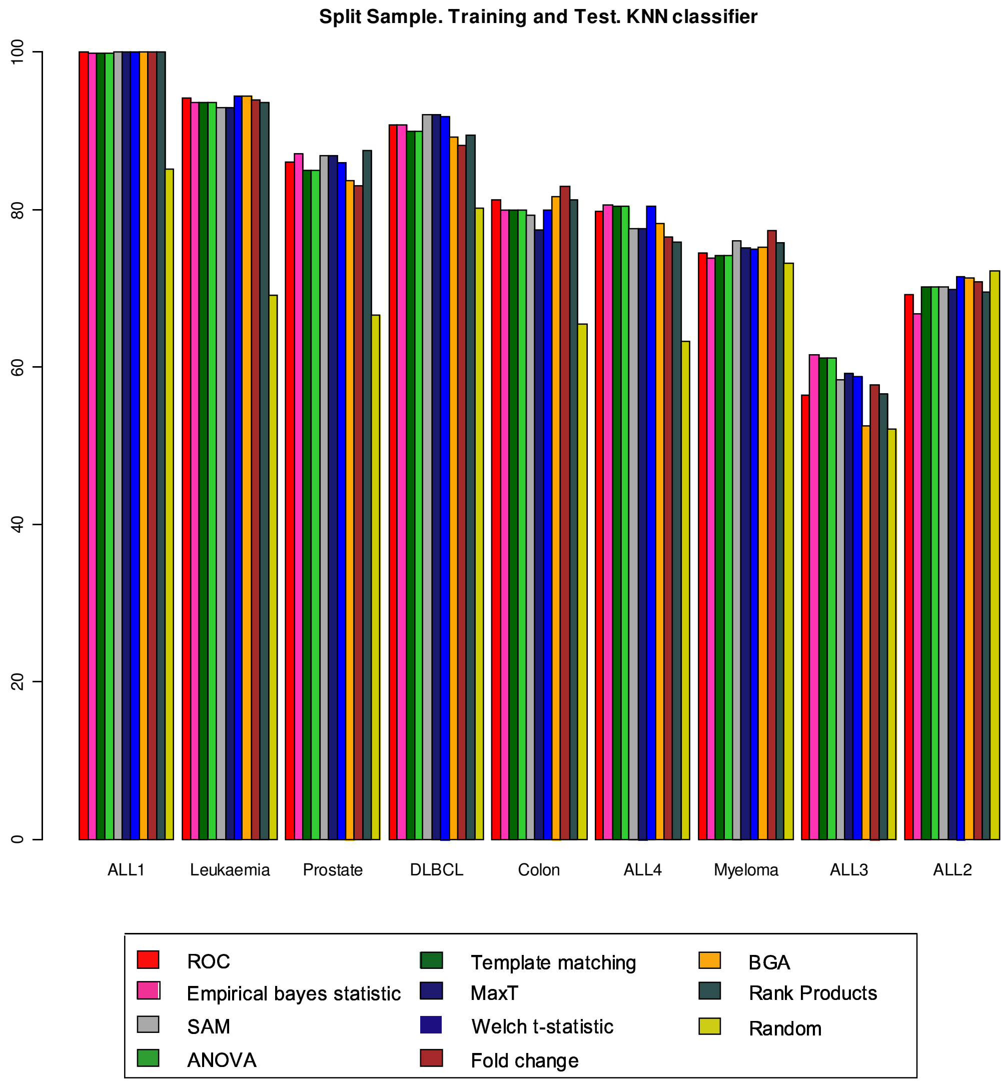
Split Sample. Training and Test. KNN classifier
0
20
40
60
80
100
ALL1
Leukaemia
Prostate
DLBCL
Colon
ALL4
Myeloma
ALL3
ALL2
ROC
Empirical bayes statistic
SAM
ANOVA
Template matching
MaxT
Welch t-statistic
Fold change
BGA
Rank Products
Random
